# Structure and expression of *Rhodnius prolixus* GH18 chitinases and chitinase-like proteins: Characterization of the physiological role of *RpCht7*, a gene from subgroup VIII, in vector fitness and reproduction

**DOI:** 10.3389/fphys.2022.861620

**Published:** 2022-10-03

**Authors:** Maiara do Valle Faria Gama, Caroline Silva Moraes, Bruno Gomes, Hector Manuel Diaz-Albiter, Rafael Dias Mesquita, Eloy Seabra-Junior, Patrícia Azambuja, Eloi de Souza Garcia, Fernando Ariel Genta

**Affiliations:** ^1^ Instituto Oswaldo Cruz, FIOCRUZ, Rio de Janeiro, Brazil; ^2^ El Colegio de la Frontera Sur, ECOSUR, Campeche, Mexico; ^3^ Departamento de Bioquímica, Instituto de Química, Universidade Federal do Rio de Janeiro, Rio de Janeiro, Brazil; ^4^ Instituto Nacional de Ciência e Tecnologia em Entomologia Molecular, Rio de Janeiro, Brazil; ^5^ Universidade Federal Fluminense, UFF, Rio de Janeiro, Brazil

**Keywords:** chitinase, *Rhodnius prolixus*, RNAi, gene expression, oviposition, glycoside hydrolase family 18, chitinase-like protein

## Abstract

Chitinases are enzymes responsible for the hydrolysis of glycosidic linkages within chitin chains. In insects, chitinases are typically members of the multigenic glycoside hydrolase family 18 (GH18). They participate in the relocation of chitin during development and molt, and in digestion in detritivores and predatory insects, and they control the peritrophic membrane thickness. Chitin metabolism is a promising target for developing vector control strategies, and knowledge of the roles of chitinases may reveal new targets and illuminate unique aspects of their physiology and interaction with microorganisms. *Rhodnius prolixus* is an important vector of Chagas disease, which is caused by the parasite *Trypanosoma cruzi*. In this study, we performed annotation and structural characterization of nine chitinase and chitinase-like protein genes in the *R. prolixus* genome. The roles of their corresponding transcripts were studied in more depth; their physiological roles were studied through RNAi silencing. Phylogenetic analysis of coding sequences showed that these genes belong to different subfamilies of GH18 chitinases already described in other insects. The expression patterns of these genes in different tissues and developmental stages were initially characterized using RT-PCR. RNAi screening showed silencing of the gene family members with very different efficiencies. Based on the knockdown results and the general lack of information about subgroup VIII of GH18, the *RpCht7* gene was chosen for phenotype analysis. *RpCht7* knockdown doubled the mortality in starving fifth-instar nymphs compared to dsGFP-injected controls. However, it did not alter blood intake, diuresis, digestion, molting rate, molting defects, sexual ratio, percentage of hatching, or average hatching time. Nevertheless, female oviposition was reduced by 53% in *RpCht7*-silenced insects, and differences in oviposition occurred within 14–20 days after a saturating blood meal. These results suggest that *RpCht7* may be involved in the reproductive physiology and vector fitness of *R. prolixus*.

## 1 Introduction

Chagas disease is a parasitic, systemic, and chronic condition caused by the protozoan parasite *Trypanosoma cruzi*, and its risk factors are strongly associated with low socioeconomic status. *T. cruzi* is mainly transmitted through triatomine feces during blood-feeding in a vertebrate host or by ingesting food contaminated with the parasite (PAHO, 2020; WHO, 2020). *T. cruzi* develops cyclically between invertebrate (triatomine) and vertebrate (mammalian) hosts. This protozoan’s life cycle adopts different morphological types that vary in physical form, metabolism, and membrane components (Garcia and Azambuja, 1991). Chagas disease is considered a neglected tropical disease and is endemic in 21 countries in the Americas, although the migration of infected people can transport the disease to non-endemic countries. Integrated vector control is the prevention method most widely used in Latin America (PAHO, 2020; WHO, 2020).

*Rhodnius prolixus* is a hematophagous Hemiptera belonging to the Triatominae family, Reduviidae subfamily (Schofield and Galvão, 2009). It is the primary vector of Chagas disease in Colombia, Venezuela, and Central America (Coura, 2015; PAHO, 2020). Due to its great importance in Chagas disease transmission and its easy maintenance in the laboratory, *R. prolixus* has been widely used as a study model (Wigglesworth, 1933; Garcia et al., 1975; Azambuja et al., 2017).

*R. prolixus* is a hemimetabolous insect with three stages of development: egg, five nymph instars, and adult (Azambuja et al., 1981). Through ecdysis, *R. prolixus* passes through five nymph instars, reaching the adult stage after the fifth molt. *R. prolixus* molting occurs at regular intervals after blood-feeding, which has a non-continuous character (Lent, 1948; Lent and Wygodzinsky, 1979; Schofield and Galvão, 2009). Thus, a single blood meal can trigger ecdysis, regulating metabolic and hormonal processes (Wigglesworth, 1951).

Chitin is a linear polysaccharide composed of a chain of N-acetyl-β-D-glucosamine monomers linked by β-1,4 glycosidic bonds (Cohen, 1993). Chitin is a crucial constituent of the fungal cell wall and the arthropod exoskeleton cuticle, which is exchanged during molting. Additionally, it is part of the trachea and the peritrophic membrane, which is a structure in the gut of many insects (Merzendorfer and Zimoch, 2003; Arakane and Muthukrishnan, 2010). The *R. prolixus* digestive system comprises the foregut, midgut, and hindgut, with accessory organs such as the salivary glands. The midgut can be divided into anterior and posterior midguts (Wigglesworth, 1933). Unlike the peritrophic membrane found in other insects, in hemipterans such as *R. prolixus*, a perimicrovillar membrane covers the midgut epithelial cells (Billingsley and Downe, 1983; Terra, 1990). Although the perimicrovillar membrane is known for its non-chitinous lipoprotein composition, recent studies suggest that chitin is a component of the midgut epithelium of *R. prolixus* (Alvarenga, 2016). In insects, the chitin chains composing the cuticle and the peritrophic membrane are organized into structures that give the chitinous matrixes their unique properties of rigidity, elasticity, and waterproofing (Kramer and Koga, 1986; Merzendorfer and Zimoch, 2003; Pillai et al., 2009).

Chitinases (E.C 3.2.1.14) are enzymes that hydrolyze the chitin polymer. They are part of the glycoside hydrolase family 18 (GH18) (Coutinho and Henrissat, 1999). Proteins of this family have a modular structure (Arakane et al., 2003) and generally consist of a signal peptide, catalytic domain, connector domain, and chitin-binding domain. The signal peptide is not conserved, being characteristic of the considered taxon. The catalytic domain of insect chitinases has a series of conserved aspartate and tyrosine residues involved directly in catalysis (Aalten et al., 2001). The connector domain is rich in serines and threonines and appears essential for correct protein folding during secretion and for enzyme stability against the action of proteases (Arakane et al., 2003). The chitin-binding domain, rich in cysteines, is vital for chitin recognition and for activity against the insoluble form of the substrate.

In insects, chitinases of the GH18 family are generally members of a multigenic family, and, as observed in other models, there are several chitinase genes in the *R. prolixus* genome (Mesquita et al., 2015). Chitinase gene mapping, phylogenetic analysis of amino acid protein sequences, and study of its functional roles in different model insects—such as *Anopheles gambiae*, *Aedes aegypti*, *Acyrtosiphon pisum*, *Drosophila melanogaster*, *Pediculus humanus*, and *Tribolium castaneum*—have allowed the classification of these proteins into eight homologous functional groups (I–VIII) (Nakabachi et al., 2010; Zhang et al., 2011).

Several functions have been proposed for the insect chitinases. Most are involved in the molting process and participate in exoskeleton turnover (Merzendorfer and Zimoch, 2003; Arakane and Muthukrishnan, 2010). These enzymes might also regulate the synthesis and degradation of the peritrophic membrane (Cohen, 2010). In addition, some insect chitinases are related to the digestion of fungi (in detritivorous insects) or cuticles of other arthropods (in predatory insects) (Kramer and Koga, 1986; Genta et al., 2006). In recent decades, chitin metabolism has been postulated as a promising target for disruption of the insecticide development. Several molecules in the class of insecticide growth regulators (IGRs), which have important impacts on agricultural pest control, are inhibitors of chitin synthesis or turnover (Merzendorfer, 2013).

The RNAi technique has been partially elucidated in studies with organisms such as the nematode *Caenorhabditis elegans* and the fly *D. melanogaster* (Roignant et al., 2003; Bischoff et al., 2006; Miller et al., 2008). As a fast and straightforward technique, RNAi has been widely used in gene function studies (Fjose et al., 2001). The silencing of the gene target makes it possible to determine the function of the cellular protein that a gene encodes (Morris et al., 2004).

Some insect chitinases, such as those in group VIII, remain functionally unclassified. Reverse genetics techniques such as RNAi may provide further evidence of the expression and function of these chitinase genes. Therefore, in the present work, we explored the role of these enzymes by observing the effects of chitinase inhibition in *R. prolixus* physiological parameters. Obtaining new functional data for this enzyme group may result in future targets for vector control and reveal aspects of insects’ basic physiology and interactions with microorganisms.

## 2 Materials and methods

### 2.1 Bioinformatics

Sequences of *R. prolixus* chitinases were obtained using FAT software (Seabra-Junior et al., 2011) or ClustalW and HMMER tools, using the PFAM code 00704 of the GH18 family against the *R. prolixus* genome (conserved domains on *R. prolixus*, RproC3, last updated 13 August 2015) deposited in Vector Base (http://www.vectorbase.org).

Nucleotide sequences were translated with the Expasy Translate tool (http://web.expasy.org/translate/) for subsequent alignment of amino acid sequences. Chitinase sequences were used for similarity searches in BLASTp (https://blast.ncbi.nlm.nih.gov/Blast.cgi) and then aligned with the five most similar sequences using ClustalW Multiple Alignment tool version 1.4 found in BioEdit Sequence Alignment Editor version 7.0.9.0 (Thompson et al., 1994; HALL, 1999). Further information regarding sequences, such as signal peptides (Petersen et al., 2011), N-glycosylations (Blom et al., 2004), O-glycosylations (Steentoft et al., 2013), GPI anchors (Fankhauser and Mäser, 2005), transmembrane domains (Tusnády and Simon, 2001), molecular masses, and isoelectric points (Bjellqvist et al., 1994; Wiederschain, 2006), were obtained using Expasy tools (https://www.expasy.org/proteomics).

The phylogenetic tree was constructed based on the amino acid sequences of GH18 catalytic domains using the PHYLIP package (Felsenstein, 2008). PROTDIST and NEIGHBOR were used for phylogeny calculations. After homologous models were aligned with Clustal Omega (Sievers et al., 2011), the tree was visualized with the FIGTREE program (Suchard and Rambaut, 2009). Bootstrap analysis with a data set of 1,000 replicates was created with SEQBOOT and analyzed as above before support calculation using CONSENSE. All programs were used with default parameters. The tree has been edited with GIMP version 2.8.14 (https://www.gimp.org/).

### 2.2 Insects

*R. prolixus* (Hemiptera: Reduviidae) were obtained from the colony of Laboratório de Bioquímica e Fisiologia de Insetos, IOC/FIOCRUZ. Insects were maintained at 28°C and usually fed defibrinated rabbit blood using an artificial feeder (Azambuja and Garcia, 1997). For quantitative analysis of *GH18* gene expression throughout development, eggs, nymphs from the first to the fifth stage, and male and female adults were used. For tissue expression analysis and silencing experiments, fifth-instar nymphs were used.

### 2.3 Qualitative analysis of gene expression

#### 2.3.1 Sample preparation

Experiments were performed with pools of 5 insect eggs or 5 first-instar nymphs. Pools of 3 insects were used for analysis with second-, third-, and fourth-instar nymphs and 2 insects for fifth-instar nymphs, male and female adults. Samples were collected at 0, 2, 5, 7, 9, 12, 14, 16, 19, 21 and 23 days after feeding (daf). Then, insect samples were placed into polypropylene microtubes, frozen in liquid nitrogen, and stored at −80°C until use.

For tissue expression analysis, *R. prolixus* fifth-instar nymphs were immobilized on ice and subsequently dissected in saline solution 0.9% (w/v) for removal of the salivary gland, anterior midgut, posterior midgut, hindgut, hemolymph, fat body, and carcass. Hemolymph samples were collected carefully from a severed leg with 10 µl calibrated micropipettes (Sigma). Tissues were stored in polypropylene microtubes (pools of 3) containing 200 μl of TRIzol. Samples were immediately frozen in liquid nitrogen and stored at −80°C until total RNA extraction.

#### 2.3.2 Extraction of total RNA from *R. prolixus*

For total RNA extraction, whole insect samples were gathered in a mortar with TRIzol (Sigma-Aldrich, Cat. No. T9424) or RNAzol (MRC, Cat. No. RN 190), following the manufacturer’s recommendations, and homogenized with the pistil in the presence of liquid nitrogen. Tissue samples were pooled in a Potter-Ehveljem apparatus (Sigma-Aldrich) and homogenized in TRIzol or RNAzol. After extraction of ribonucleic acid, the total RNA concentration was quantified using Nanodrop equipment, and the samples were stored at −80°C until use.

#### 2.3.3 Preparation of cDNA

Total RNA obtained was submitted to reverse transcription with the Superscript III First-Strand kit (Invitrogen, Cat. No. 18080-051) following the manufacturer’s recommendations. cDNA samples were stored at −20°C (Sambrook and Russell, 2001).

#### 2.3.4 Polymerase chain reaction

cDNA samples were used in amplification reactions in a thermocycler (Veriti, Applied Biosystems) using primers (Supplementary Table S1) designed for annealing in nonconserved regions of GH18 family genes, using the tools at the IDT website (https://www.idtdna.com/Primerquest/Home/Index) with default parameters, selecting only optimal amplicon size. Each reaction contained 1 μl of 50 ng cDNA, 4 μl of 10X Taq buffer, 3.2 μl of 25 mM MgCl_2_, 0.4 μl of 10 mM dNTP, 1 μl of each primer at 10 mM, 0.1 μl of GoTaq DNA polymerase (Promega, United States), and water to a final volume of 20 μl. Amplifications were performed at 94°C for 2 min, followed by 35 denaturation cycles at 94°C for 15 s, annealing at 55°C for primers *RpCht1*, *2*, *3*, *4*, *5*, *6*, *8*, and *9* and at 60°C for *RpCht7* for 30 s, and extension at 72°C for 1 min, with a final extension at 72°C for 5 min. The amplicons were stored at −20°C until analysis by agarose gel electrophoresis at 1.5% (w/v) and stained with ethidium bromide (Sigma, Cat. No. 1239-45-8). The gel was photographed, and the image was analyzed with the ImageJ program (https://imagej.nih.gov/ij/). The intensity of each band, as well as the background and the band of the constitutive gene, were quantified. The amplification product was excised from the gel, purified with the NucleoSpin Gel and PCR Clean-up kit (Macherey-Nagel) following the manufacturer’s recommendations, and sent to the DNA Sanger sequencing service of Fiocruz/IOC. To produce dsRNA, we used specific primers conjugated to 23 bases of T7 RNA polymerase promoter (Supplementary Table S1) to obtain 2 independent PCR reactions with a single T7 promoter. Primers were designed for annealing to nonconserved regions of each chitinase gene (data not shown). The annealing temperature was 60°C for primers *RpCht1*, *2*, *3*, *4*, *6*, *7*, *8*, and *9*. It was not possible to amplify the *RpCht5* gene. The amplicons were stored at −20°C until use.

### 2.4 RNAi silencing

#### 2.4.1 Synthesis of dsRNA

PCR products were purified with NucleoSpin Gel and PCR Clean-up (Macherey-Nagel) and used as templates for dsRNA synthesis using the RiboMAX express T7 RNAi system (Promega, United States) following the manufacturer’s recommendations. The concentration of dsRNA was measured with the Nanodrop equipment, and samples were stored at −20°C.

#### 2.4.2 Injection of dsRNA

The injections were performed using a microinjector (Nanoinjector, Drummond, United States) in the thoracic hemolymph (Paim et al., 2013) with a capillary of 0.05 mm diameter, which avoided mortality and considerable damage to the insect cuticle. The chitinase dsRNAs and the control of exogenous dsRNA (dsGFP, green fluorescent protein) were injected into separate groups of insects. Initially, we injected different amounts of dsRNA (1 μg, 3 μg, 6 μg, and 2 × 3 μg). We decided to use the amount of 1 μg in subsequent experiments based on the results. The dsRNA was injected into three groups of 5 insects for each amount: 1 µg, 3 µg, 6 µg, and 2 × 3 µg of dsRNA per insect. In the group that received 2 × 3 µg, the second injection of 3 µg was performed 48 h after the first (Paim et al., 2013). The insects used for the silencing experiments were synchronized. The injection was done in the fifth-instar nymphs 1 week after ecdysis.

To check the persistence of *RpCht7* knockdown, three groups with 20 *R. prolixus* fifth-instar nymphs were injected with 1 µg ds*RpCht7*. We used dsGFP as a control for exogenous dsRNA to see if the effects were the result of a generic exposure to dsRNA. We also injected water as a control to observe effects related to injury caused by microinjection. We extracted total RNA from 3 insects per experimental group to confirm the silencing. Extractions were performed at 2, 16, and 51 days after injections.

### 2.5 Phenotypic analysis

To observe the phenotypes, insects were synchronized by separating a group of 200 fully-engorged fourth-instar nymphs of *R. prolixus* immediately after blood feeding and accompanying the ecdysis. For dsRNA injections, only fifth-instar nymphs collected 7 days after ecdysis were used. After inoculation with dsRNA, different insect developmental parameters, such as mortality, ecdysis, ecdysis defects, sex ratio, oviposition, egg hatching, blood-feeding, diuresis, and digestion, were monitored as described below.

#### 2.5.1 Mortality

Insect mortality was followed in 3 replicates, each containing 20 *R. prolixus* fifth-instar nymphs, totaling 60 insects per condition and 120 insects per experiment. These insects were observed every 2 days for approximately 15 days, until the blood-feeding of the fifth-instar nymphs.

#### 2.5.2 Blood ingestion and diuresis

The insects were individually weighed and fed with defibrinated rabbit blood in an artificial feeder (Azambuja and Garcia, 1997); treatment groups were kept separate. The triatomines were weighed again immediately after the blood meal. The individual weighing procedure was repeated at 24 h, 48 h, and 7 daf.

#### 2.5.3 Ecdysis

After the end of the molting of the insects of item 2.5.2, from the fifth to the adult stage, we registered the number of insects that failed ecdysis. We counted separately how many insects remained as fifth-instar nymphs, how many were trapped in the exuvia, how many died during the molting, and the total number of male and female adults obtained in each group.

#### 2.5.4 Adults’ blood intake

*R. prolixus* adults were individually weighed 2 weeks after the molt and fed with defibrinated rabbit blood in an artificial feeder (Azambuja and Garcia, 1997). Immediately after the blood meal, the triatomines were weighed individually again.

#### 2.5.5 Oviposition and egg hatching

After feeding, insects were separated into groups with one female and two males each. All were injected with the same dsRNA. Insects were checked every 3 days until their death. During that period, the numbers of dead and hatched eggs and the mortality of adults were verified.

### 2.6 Statistical analysis

Graphs and data analyses were performed using GraphPad Prism version 5.01, unpaired Student’s *t*-test, or one-way ANOVA with post-hoc Tukey’s multiple comparisons test. All results with a *p*-value < 0.05 were classified as statistically significant and specified in the graphs.

## 3 Results

### 3.1 Sequence analysis of GH18 genes in *R. prolixus*

During the annotation of the *R. prolixus* genome, nine genes encoding proteins of glycoside hydrolase family 18 (GH18) were found. To confirm if those genes have homologs in other insect genomes, their sequences were named *RpCht1* to *RpCht9* and were used as queries in BLASTp searches against databases from different insect orders (Supplementary Figures S1A–I). *R. prolixus* chitinase and chitinase-like protein coding sequences showed high identity values compared to other GH18 sequences. The identity between *R. prolixus* chitinases and their homologs varied considerably when considering different subgroups of chitinases. For example, *RpCht1* had between 48% and 52% identity with *MaCht1*, *TcCht6*, *TcCht10*, *TmCht1*, and *MaCht2* genes, while *RpCht2* showed values from 19% to 36% when compared to *AcypiCht1*, *DpCht1*, *TcCht1*, *DgCht1*, and *DaCht1*. The identity of *RpCht3* with other GH18 sequences varied between 27% and 30% (*BmCht3*, *BmCht1*, *DpCht1*, *CcCht1*, and *AgCht1*), and for *RpCht4* this value fluctuated between 32% and 35% (*TcCht3*, *PhCht1*, *DpCht3*, *DpCht4*, and *MrCht4*). For *RpCht5*, we observed values ranging from 35% to 39% (*BmCht2*, *TcCht3*, *PhCht1*, *DpCht3*, and *DpCht4*), for *RpCht6* from 55% to 65% (*PcCht1*, *TcCht2*, *TcCht5*, *NvCht1*, and *DpCht2*), for *RpCht7* from 26% to 35% (*AmCht1*, *AdCht1*, *BiCht1*, *CbCht1*, and *BtCht1*), for *RpCht8* from 38% to 59% (*OnCht1*, *MbCht1*, *MrCht1*, *AmCht2*, and *AmCht3*), and for *RpCht9* from 68% to 69% (*PcCht1*, *TcCht2*, *TcCht5*, *NvCht1*, and *DpCht2*).

To corroborate their roles in chitin hydrolysis, the best hits and *RpCht1-9* were used for amino acid sequence alignments (Supplementary Figures S1A–I), which showed a series of conserved residues, especially catalytic residues. These included the consensus sequence FDGXDLDWEYP, which is characteristic of chitinase proteins. In 8 of the 9 chitinase sequences of the *R. prolixus* genome, we found conserved catalytic residues responsible for the hydrolysis of glycosidic bonds. The only sequence that did not show the conserved catalytic residues was *RpCht8*.

We analyzed the peptide sequences of RpCht1-9 in search of structural signatures that might indicate the functional properties of these proteins. Considering all nine sequences, the theoretical isoelectric point ranged from 5.25 to 8.84, and the predicted molecular mass ranged from 38 to 297 kDa (Supplementary Table S2). In addition, some protein sequences had a prediction for signal peptides being putatively exported to the extracellular medium. The Signal IP 3.0 program identified signal peptides in 6 sequences of *R. prolixus* chitinases, while version 4.1 located signal peptides in 4 sequences only. Given this disagreement, we decided to use one more program for this prediction, Phobius IP, which also identifies transmembrane domains. Phobius IP identified 5 sequences with a signal peptide. Analyzing the three programs in parallel, we could observe that sequences RpCht1, RpCht3, RpCht4, and RpCht8 were marked by the three programs, while sequence RpCht2 was marked by Signal IP 3.0 and Phobius IP. Sequence RpCht7 was marked only by Signal IP3.0. Based on these results, we decided to use the Phobius IP program. *R. prolixus* chitinase sequences that we consider likely to be secreted into extracellular medium are RpCht1, RpCht2, RpCht3, RpCht4, and RpCht8. None of the *R. prolixus* chitinase sequences exhibited a signature for GPI anchoring. However, 8 of the 9 protein sequences had predictions for one transmembrane helix (Supplementary Table S2).

Glycosylation plays a significant role in the structure and function of extracellular chitinases. To confirm if sequences RpCht1-9 have the glycosylation patterns typical of other GH18 proteins, we analyzed this feature using the online platforms NetNGlyc and NetOGlyc. All predicted proteins exhibited putative glycosylation sites on the linker domain between the catalytic domain and chitin-binding domain (when this was present), a common characteristic of GH18 proteins. We found in these sequences from 1 to 8 N-linked glycosylation sites and from 0 to 185 O-linked glycosylation sites.

**FIGURE 1:**
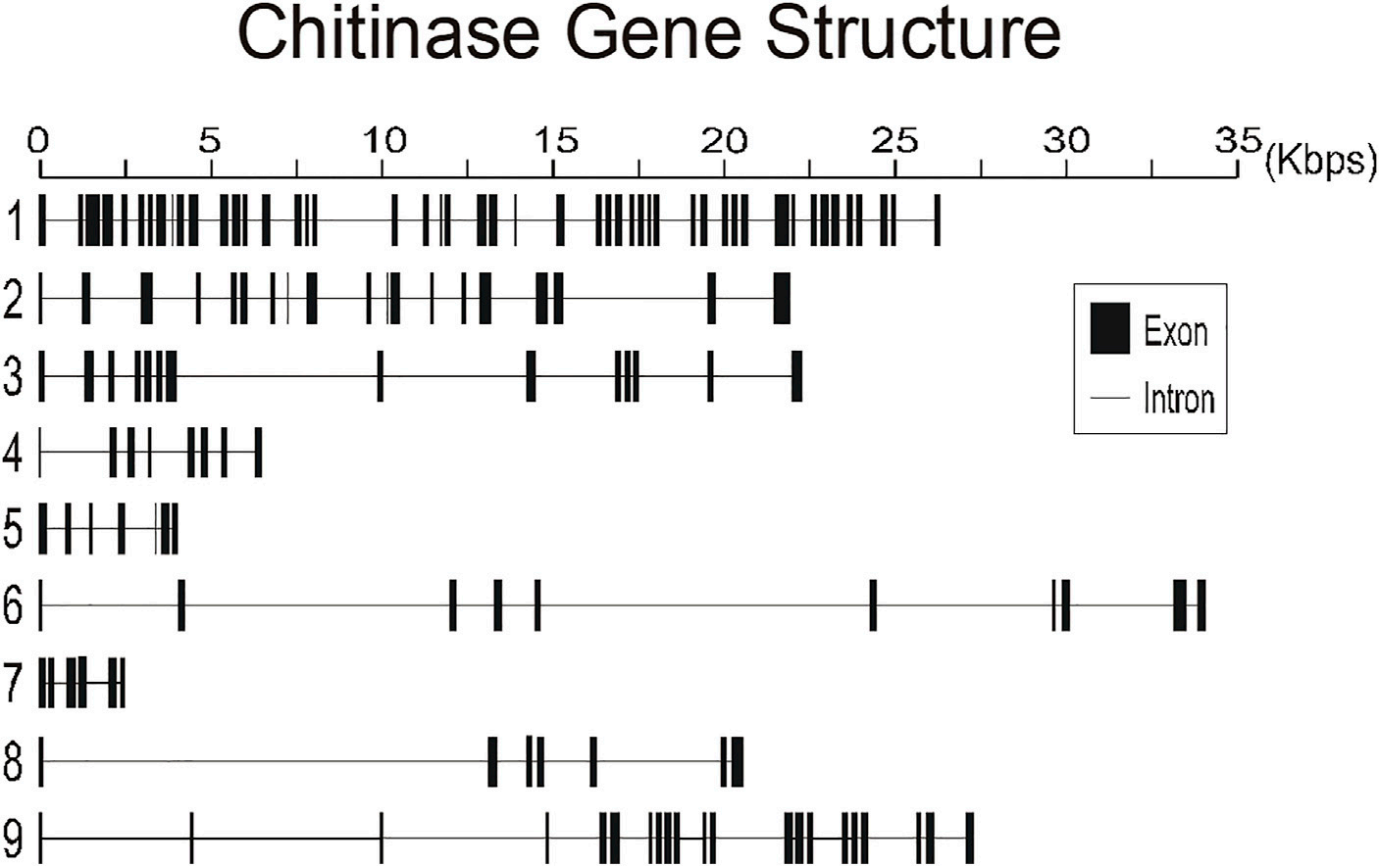
Schematic diagram of exon and intron organization of chitinase genes *RpCht1-9* from *R. prolixus*. Black boxes: exons. Gray lines: introns. Numbers 1–9 represent genes *RpCht1-9*, respectively.

**FIGURE 2:**
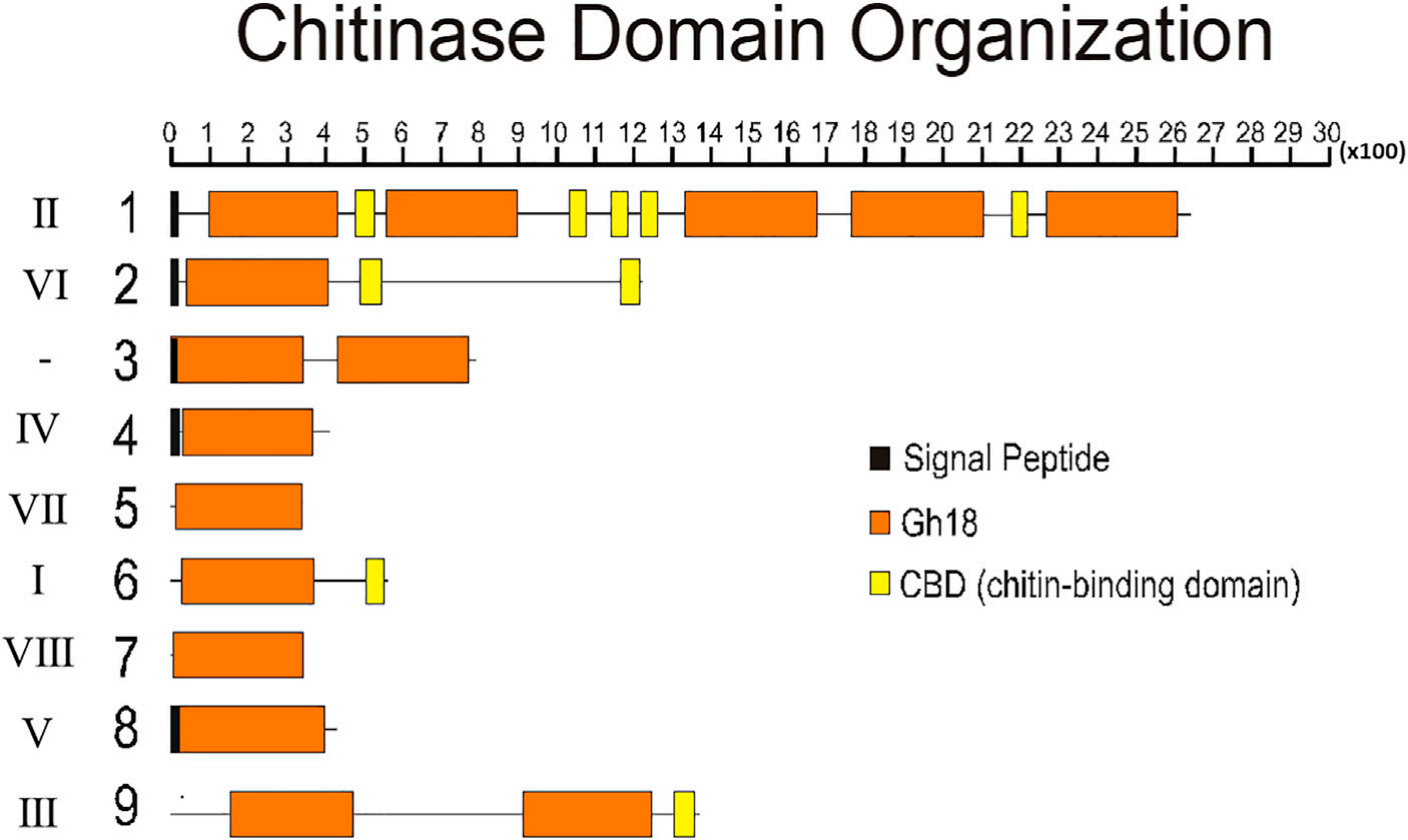
Schematic diagram of the domain architecture of chitinases from *R. prolixus*. Signal peptides are boxed in black, catalytic domains boxed in orange, and chitin-binding domains boxed in yellow. Numbers 1–9 represent proteins RpCht1-9, respectively. Roman numbers represent chitinase families. x100 = lenght of chain, in aminoacid residues.

To better understand the structure of *R. prolixus* chitinases and to allow the analysis of relationships between members of the chitinase family, we performed a detailed annotation of their modular elements. *RpCht1-9* genes showed from 6 to 46 exons, which differ structurally from each other (Figure 1). The coded proteins presented different modular organizations containing 1 to 5 catalytic domains. All these domains had the residues involved in catalysis (breakage of the glycosidic bonds), and we observed from 0 (absence) to 5 chitin-binding domains (Figure 2).

**FIGURE 3:**
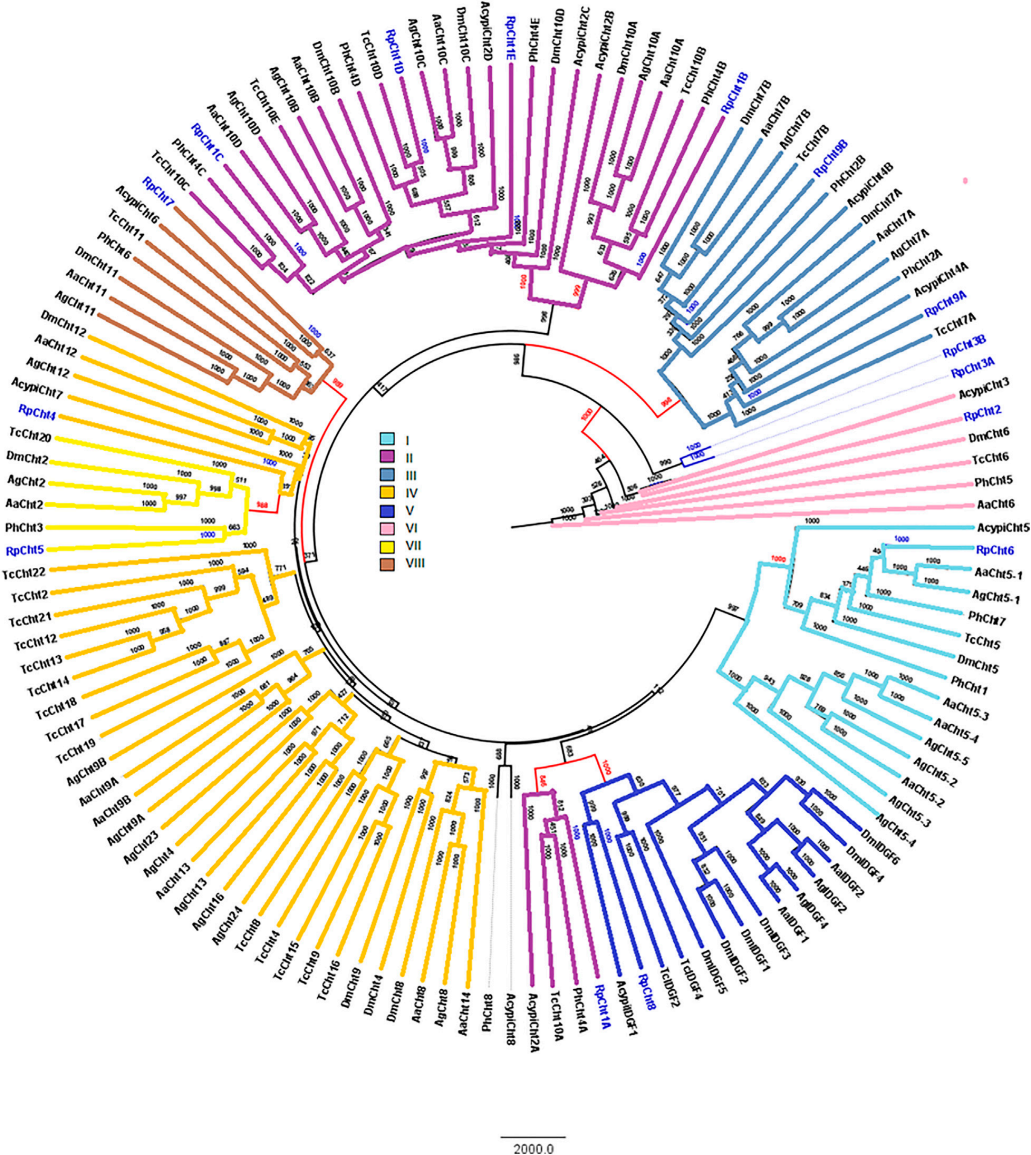
Phylogenetic analysis of chitinases catalytic domains from seven insect species based on protein sequences. Rp: *Rhodnius prolixus*; Ag, *Anopheles gambiae*; Aa, *Aedes aegypti*; Tc, *Tribolium castaneum*; Dm, *Drosophila melanogaster*; Ph, *Pediculus humanus*; Acypi, *Acyrthosiphon pisum*. Phylogenetic tree of insect chitinases generated by FigTree software (http://tree.bio.ed.ac.uk/software/figtree/) after alignment using ClustalW (www.ebi.ac.uk/clustalW). Bootstrap values were obtained by the neighbor-joining method using 10,000 replications. Colors and Roman numbers represent chitinase families.

RpCht1-9 protein sequences were compared to the GH18 proteins of other insects. Through phylogenetic analyses of coding sequences, we observed that these genes belong to different subgroups of chitinases already described in other insects. The *RpCht6* gene grouped with chitinases of family I, *RpCht1* with family II, *RpCht9* with family III, *RpCht4* with family IV, *RpCht8* with family V, *RpCht2* with family VI, *RpCht5* with family VII, and *RpCht7* with family VIII. *RpCht3* did not group with any of the chitinase families described (Figure 3).

We compared the exon numbers of *R. prolixus* sequences with *A. gambiae*, *A. aegypti*, and *P. humanus* sequences for each group of the phylogenetic tree (Supplementary Table S3). For example, we observed that *RpCht1* had 46 exons, *AaCht10* and *AgCht10* had 9 exons, and the *PhCht4* sequence was closer to *RpCht1* with 34 exons. *RpCht2* had 18 exons, the same amount as *PhCht5*, although *AaCht6* had only 9 exons. The same pattern was repeated for the other sequences. Based on these analyses, we concluded that the number of exons in *R. prolixus* sequences resembled the quantities observed in *P. humanus* more than those in *A. gambiae* or *A. aegypti*.

The molecular masses of *R. prolixus* chitinases predicted proteins are close to the molecular masses observed in the corresponding chitinase groups of other insects (Supplementary Table S4 **)**. RpCht1, RpCht4, RpCht5, RpCht6, RpCht7, RpCht8, and RpCht9 have molecular masses very similar to those observed in the homologous proteins of the correspondent chitinase groups. However, the predicted molecular mass of RpCht2 is 138 kDa, slightly lower than that of the other members of the group (VI), which range from 200 to 500 kDa.

Regarding the number of catalytic domains of GH18 proteins, we compared all the sequences that fit into one of the chitinase groups. *RpCht1* gene has 5 catalytic domains, which was consistent with other sequences of group II, which have 4 or 5 catalytic domains. *RpCht9* has two catalytic domains, the same amount as other group III sequences. *RpCht4* has only one catalytic domain and was included in group IV, which has 1 or 2 catalytic domains. *RpCht2*, *RpCht5*, *RpCht6*, *RpCht7*, and *RpCht8* grouped to clades VI, VII, I, VIII, and V, respectively, all sequences with only one catalytic domain as well.

The chitinase sequences RpCht4, RpCht5, RpCht7, and RpCht8 had no chitin-binding domain (CBD), nor did the homologous sequences of *A. gambiae*, *A. aegypti*, and *P. humanus*. RpCht6 and RpCht9 sequences had 1 CBD, the same amount as the other sequences in their respective groups. RpCht2 had 2 CBDs and belonged to a group of sequences with 1 or 2 CBDs. Likewise, RpCht1 had 5 CBDs, being a member of a group of sequences with a range of 4–5 CBDs. In general, we observed that the number of CBDs in *R. prolixus* chitinase sequences was compatible with the numbers observed in their homologs.

### 3.2 Semi-quantitative analysis of the expression of GH18 genes in *R. prolixus*

**FIGURE 4:**
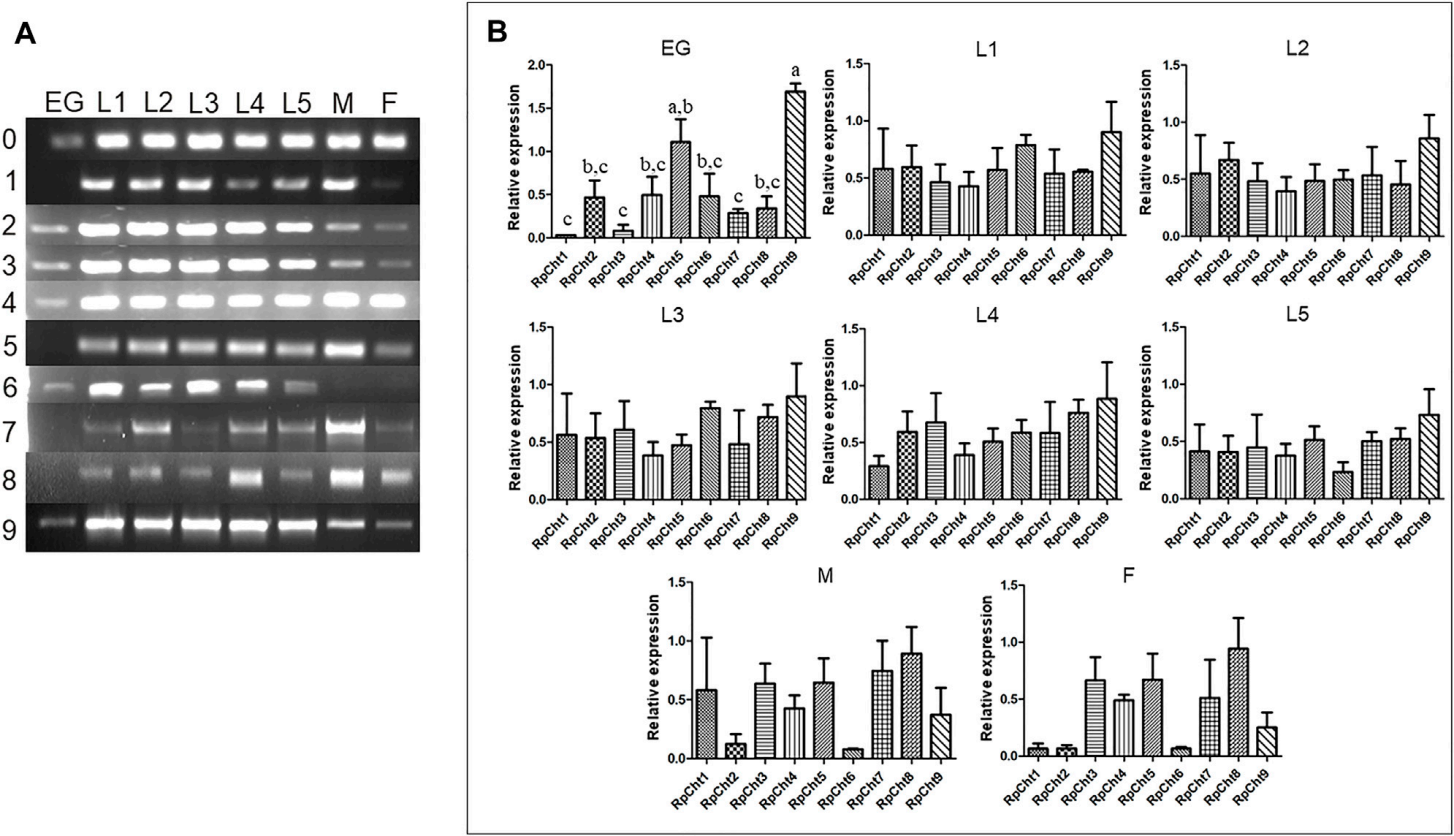
Expression profiling of chitinase genes in different developmental stages of *R. prolixus* as evaluated by RT-PCR. **(A)** Columns correspond to the result from entire insects, including nymphs (first to fifth instars: L1–L5), male (M) and female (F) adults, and eggs (EG). Lane 0 is the constitutive actin, and 1–9 represent genes *RpCht1-9*, respectively. **(B)** Relative expression with the Actin expression used as a constitutive reference gene, obtained with the ImageJ program (by band densitometry). Values marked with a, b and c correspond to significantly different groups (*p* < 0.05; one-way ANOVA–Tukey). The experiment was performed three times with independent biological replicates.

The expression of *R. prolixus* chitinases throughout insect development was analyzed to understand their function better. The chitinase genes demonstrated different expression patterns, depending on the insect’s developmental stage (Figure 4A).

We calculated relative expression using band densitometry to better describe the gene expression patterns observed during development. In the egg, *RpCht9* was expressed more than the other genes (*p* < 0.05; one-way ANOVA–Tukey), and *RpCht5* expression was significantly different when compared to *RpCht1*, *RpCht3*, and *RpCht7* (*p* < 0.05; one-way ANOVA–Tukey). However, the constitutive gene in the egg showed a band weaker than that observed in the other stages (Figure 4A), hampering comparison between eggs and other stages. In the other stages, no significant differences were observed between genes (Figure 4B).

**FIGURE 5:**
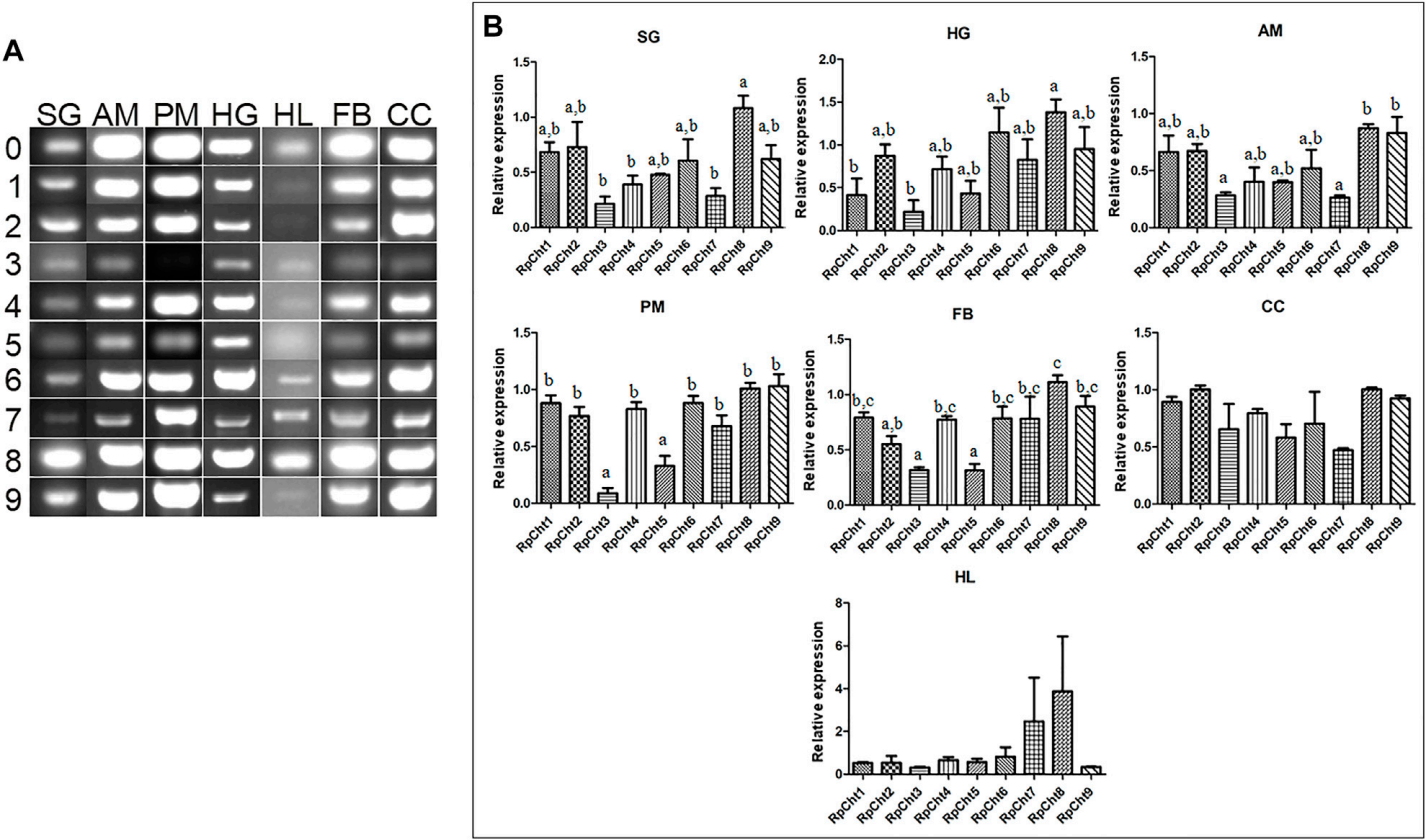
Expression profiling of chitinase genes in different tissues of *R. prolixus* fifth-instar nymphs as evaluated by RT-PCR. **(A)** Columns correspond to different tissues; SG, salivary glands; AM, anterior midgut; PM, posterior midgut; HG, hindgut; HL, hemolymph; FB, fat body; CC, carcass. Lane 0 is the constitutive reference gene (actin), and 1–9 represent genes *RpCht1-9*, respectively. **(B)** Relative expression using Actin as constitutive reference gene, obtained with the ImageJ program (by band densitometry). Values marked with a, b and c correspond to significantly different groups (*p* < 0.05; one-way ANOVA–Tukey). The experiment was performed three times with independent biological replicates.

Then we determined the expression of *RpCht* genes in different tissues of fifth-instar nymphs. This stage was chosen because it showed the expression of all the chitinase genes studied. RT-PCR reactions were performed for transcripts of each gene using cDNAs from the salivary glands (SG), anterior midgut (AM), posterior midgut (PM), and hindgut (HG) at 12 daf, and hemolymph (HL), fatty body (FB), and carcass (CC) collected 16 daf. These time points were chosen according to the chitinolytic activity peaks previously described (Faria, 2013). We observed that each chitinase gene of *R. prolixus* was expressed in all tissues tested, without an obvious pattern of expression in each organ (Figure 5A).

Relative gene expression was calculated through band densitometry to visualize better the tissue expression patterns of these genes. The salivary glands expressed the *RpCht8* gene more highly than *RpCht3*, *RpCht4*, and *RpCht7* (*p* < 0.05; one-way ANOVA–Tukey). In the posterior midgut, *RpCht3* and *RpCht5* expression (*p* < 0.05; one-way ANOVA–Tukey) were significantly different as compared to the other genes. In the hindgut, *RpCht8* expression was higher than *RpCht1* and *RpCht3* (*p* < 0.05; one-way ANOVA–Tukey). In the fat body, *RpCht8* expression was higher than *RpCht2*, *RpCht3*, *RpCht5*, and *RpCht3*, and *RpCht5* expression was lower than the other genes (*p* < 0.05; One-way ANOVA–Tukey). In the anterior midgut, *RpCht8* and *RpCht9* expression were higher than *RpCht3* and *RpCht7* (*p* < 0.05; one-way ANOVA–Tukey). In the hemolymph and carcass, we did not find significant differences between any genes (one-way ANOVA–Tukey) (Figure 5B). The patterns of tissue and temporal expression above were used to choose conditions for silencing and physiological function experiments.

### 3.3 Screening of GH18 transcripts by RNAi silencing

To define the lowest concentration of dsRNA that resulted in a significant percentage of silencing, we performed a concentration curve by injecting different amounts of dsRNA per insect for the 8 chitinase genes for which we were able to synthesize dsRNA.

**FIGURE 6:**
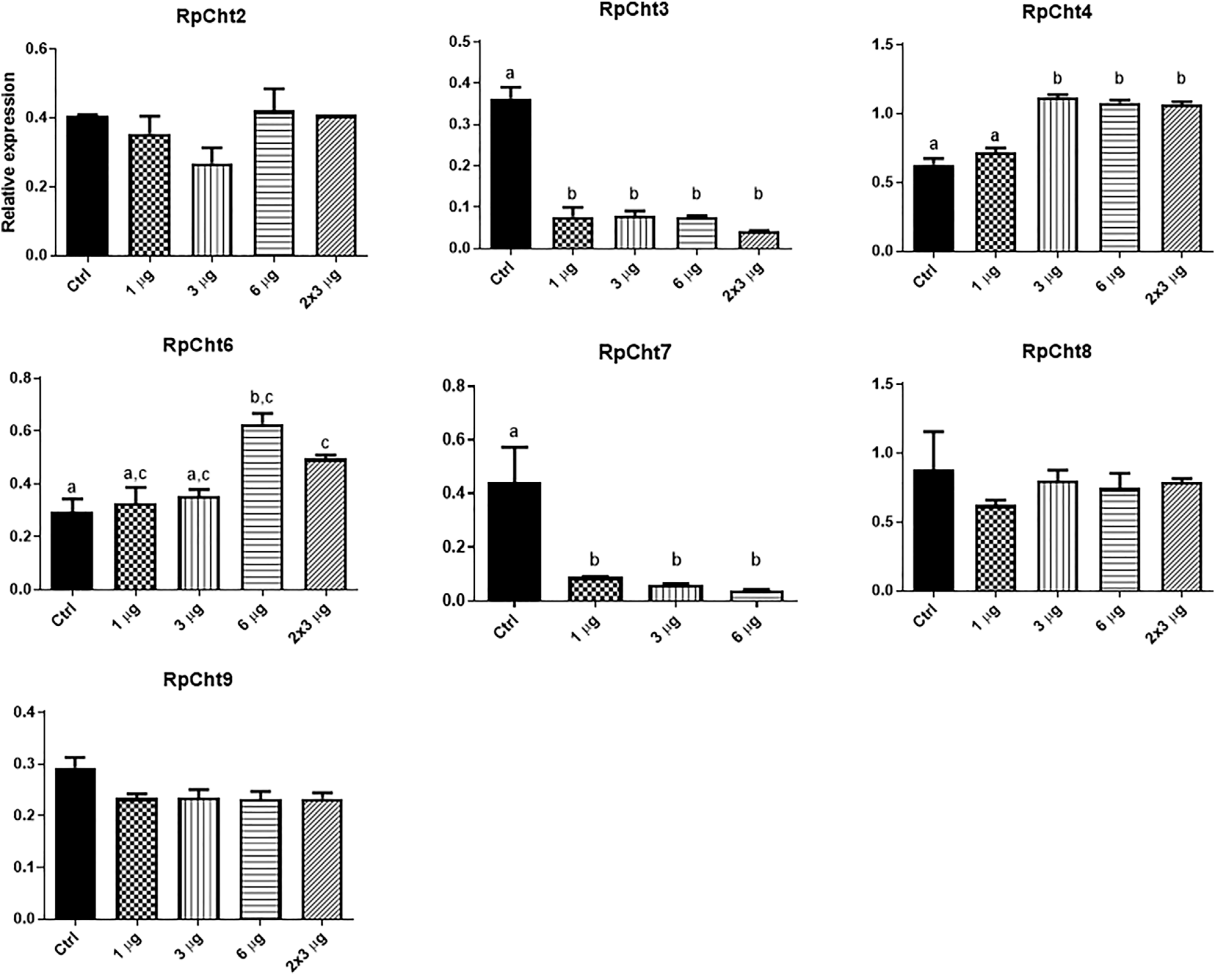
Knockdown screening of chitinase genes. Fifth instar nymphs of *R. prolixus* were injected into the hemolymph with dsRNA for *GFP* as control, and *RpCht2*, *RpCht3*, *RpCht4*, *RpCht6*, *RpCht7*, *RpCht8* and *RpCht9* with 1 µg, 3 µg, 6 µg and two injections of 3 µg with a 24 h interval between them. Relative expression using Actin as reference constitutive gene, obtained with the ImageJ program (by band densitometry). Values marked with a, b and c correspond to significantly different groups *p < 0.0001 (one-way ANOVA–Tukey), *N* = 3.

The *RpCht5* gene was not screened because the primers with the T7 sequence did not result in any product after numerous RT-PCR reactions using different conditions and cDNA templates (data not shown). After the final injections, we extracted the total RNA for *RpCht8* and *RpCht4* from a pool of 3 insects. For *RpCht1*, *RpCht2*, *RpCht3*, *RpCht6*, *RpCht7*, and *RpCht9* genes, we decided to extract the messenger RNA (mRNA) from 3 insects separately, in order to work with clean samples and observe silencing individually. However, we did not obtain results for the *RpCht1* gene because its mRNA proved unstable and easily degradable (data not shown); therefore, we had to repeat the experiment using total RNA for this gene. The total or messenger RNA was subjected to reverse transcription, and the cDNA obtained was used in PCR reactions to confirm silencing. Densitometry was measured for bands obtained on agarose gel, and we observed no silencing for the *RpCht2*, *RpCh8*, and *RpCht9* genes as compared to GFP controls (one-way ANOVA–Tukey). *RpCht3* and *RpCht7* genes had significant silencing with an approximate 80% reduction (*p* < 0.0001and *p* = 0.008; one-way ANOVA–Tukey). *RpCht4* and *RpCht6* genes showed a significant increase in their expression in some groups (*p* = 0.0008; one-way ANOVA–Tukey) (Figure 6).

### 3.4 Persistence of silencing and phenotype analysis

**FIGURE 7:**
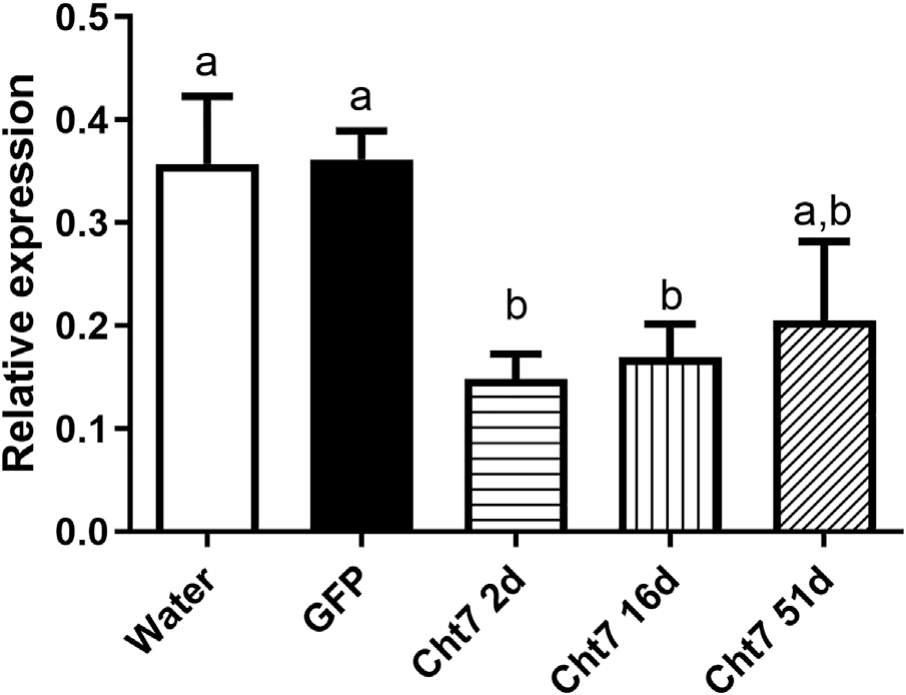
Persistence of silencing of *RpCht7* chitinase gene evaluated by RT-PCR. Fifth-instar nymphs of *R. prolixus* were injected into hemolymph with water or *GFP* dsRNA as control and 1 µg for *RpCht7* dsRNA and assessed in 2, 16, and 51 days after injection. Relative expression using Actin as constitutive reference gene, obtained with the ImageJ program (by band densitometry). Values marked with a or b correspond to significantly different groups (*p* < 0.05; T-test), *N* = 3.

We chose the *RpCht7* gene for subsequent studies because it presented an interesting preliminary mortality phenotype beyond the high silencing rate (data not shown). To reduce the chance of side effects and non-specific responses, we used injections of 1 µg per insect since that was the lowest amount to result in a significant silencing. Silencing decreased over the days; in other words, the expression levels of *RpCht7* increased over time (Figure 7). However, the expression levels of *RpCht7* at 2, 16, and 51 days after injections were significantly lower than the controls (*p* < 0.05, T-test), and silencing was still considered sufficient for phenotype analysis.

**FIGURE 8:**
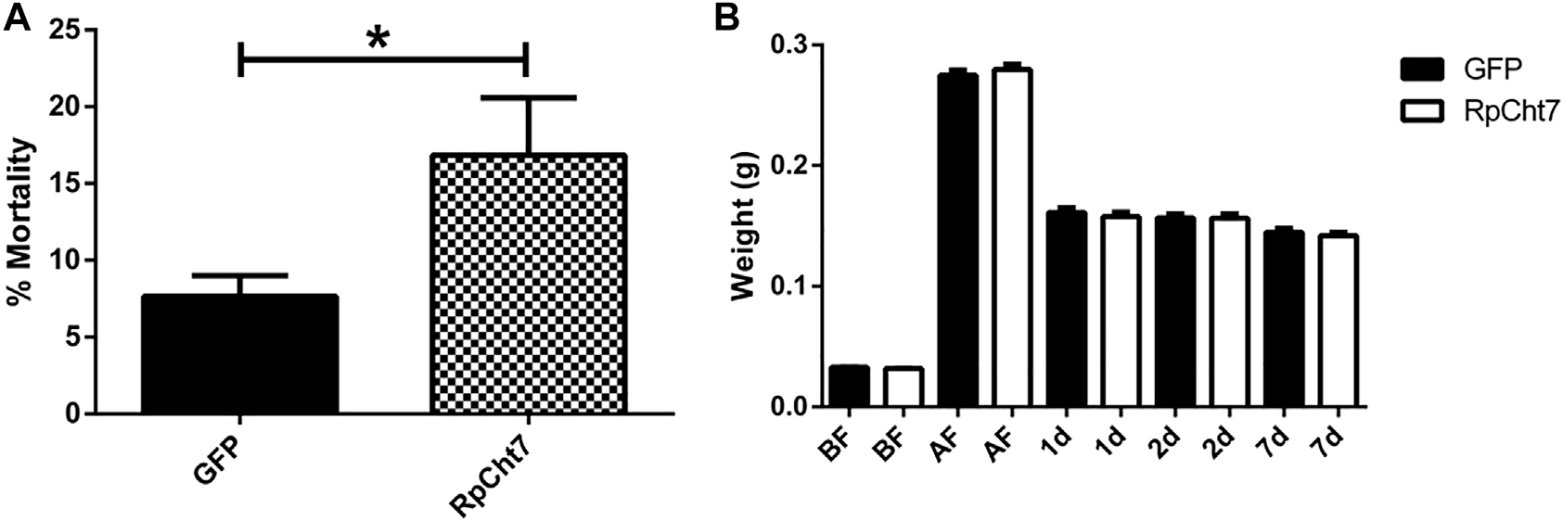
**(A)** Mortality in fifth-instar nymphs of *R. prolixus*. Percentage of dead insects 2 weeks after injection with ds*GFP* or ds*RpCht7*. The experiment was performed six times, independently with 20–30 insects each time (biological replicas, *n* = 6), *p < 0.001 (T-test). **(B)** Weight of fifth-instar nymphs of *R. prolixus*. BF, Before feeding; AF, After feeding, 1, 2, and 7 days after feeding. ds*GFP*: Black columns; ds*RpCht7*: White columns. *N* = 9. No significant differences were observed between ds*RpCht7* and ds*GFP* groups (*p* > 0.05; one-way ANOVA–Tukey).

Fasting *R. prolixus* fifth-instar nymphs injected with dsRNA for the *RpCht7* gene showed mortality rates significantly higher than controls injected with dsGFP (*p* = 0.0048; T-test). Nymph mortality was monitored for approximately 15 days, with observations made every 2 days. During this period, nymphs injected with ds*RpCht7* reached a total mortality rate of around 20% (Figure 8A).

To verify whether *knockdown* of the *RpCht7* gene affected blood intake levels, diuresis, or digestion, silenced fifth-instar nymphs were weighed individually before and after a blood meal. Insects were weighed before and immediately after a feed to see if the blood intake was affected, 24 and 48 h after a feed to observe changes in diuresis, and 7 daf for general analysis of digestion and development. There were no significant differences (one-way ANOVA–Tukey) between control groups (injected with ds*GFP*) and experimental groups (injected with ds*RpCht7*) in all those parameters. In general, silencing the *RpCht7* gene did not affect ingestion, diuresis, or digestion (Figure 8B).

**FIGURE 9:**
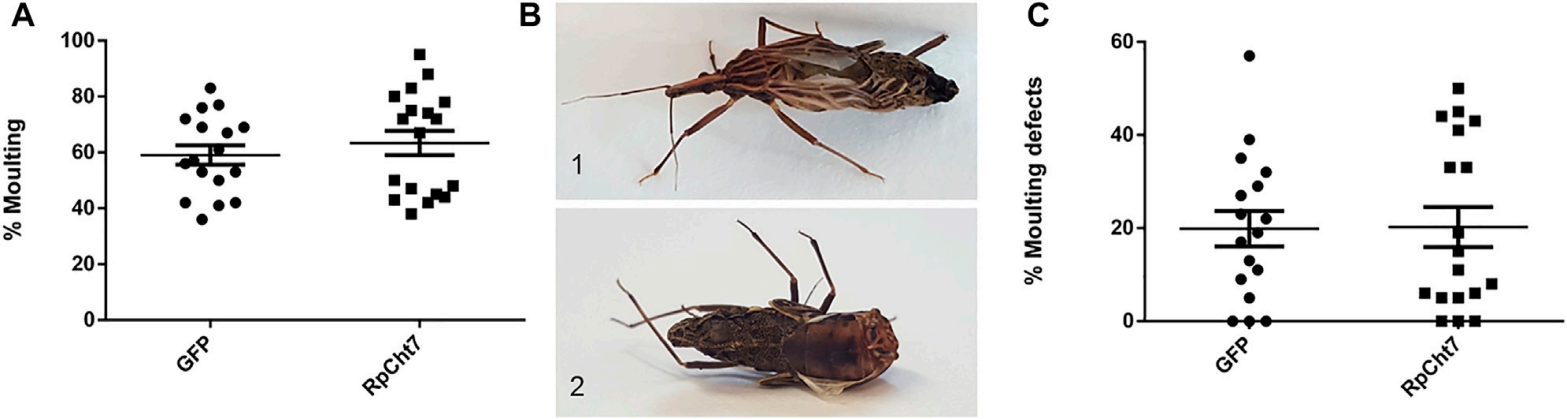
Molting phenotype. **(A)** Percentage of fifth-instar nymphs that molted to adults after injection with dsRNA and blood meal. **(B)** Image of defective phenotype; 1: Insect trapped in exuvia 2: Deformed insect. **(C)** Percentage of all molting defects. GFP: injected with ds*GFP*; RpCht7: injected with ds*RpCht7*. The experiment was performed 8 times with groups of 20–30 insects each. No significant differences were found between GFP and RpCht7 groups (*p* > 0.05; T-test).

Another phenotype evaluated was the percentage of insects that carried out ecdysis to reach adulthood. There was no significant difference in the percentage of molted insects between the groups injected with ds*GFP* and ds*RpCht7* (*p* > 0.05; T-test). Both groups had a proportion of approximately 60% ecdysis (Figure 9A). At the end of the observation, we noticed that some insects were trapped inside the exuvia (Figure 9B1) and others died during the molt (Figure 9B2). We therefore decided to account for defects in molt and identify any differences between both groups. The percentage of molt defects was approximately 20% in the ds*GFP* group and approximately 25% in the group injected with ds*RpCht7*. There was no significant difference between the two groups (*p* > 0.05; T-test). Thus, silencing the *RpCht7* gene did not affect the percentage of molt defects (Figure 9C).

**FIGURE 10:**
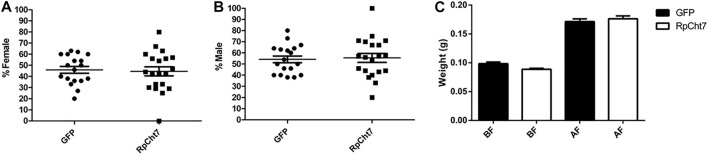
Sex ratio in *R. prolixus* adults after dsRNA injection in fifth-instar nymphs. **(A)** Percentage of females. **(B)** Percentage of males. GFP: injected with ds*GFP*; RpCht7: injected with ds*RpCht7*. The experiment was performed 8 times with groups of 20–30 insects each. No significant differences were found between GFP and RpCht7 groups (*p* > 0.05; *t*-test). **(C)** Adult insect weight of *R. prolixus* injected with dsRNAs in fifth-instar nymphs. BF, before feeding; AF, after feeding. ds*GFP*: black; ds*RpCht7*: White. The experiment was performed 8 times with groups of 15–25 insects each. No significant differences were found between *GFP* and *RpCht7* groups (*p* > 0.05; one-way ANOVA–Tukey).

We also analyzed the sex ratio in adult insects to verify if *RpCht7* gene silencing had any effect on the development or molt of males or females. There was no significant difference between the groups injected with ds*GFP* or ds*RpCht7* (*p* > 0.05; T-test). Both groups had approximately 45% females (Figure 10A) and 55% males (Figure 10B).

Blood intake in adult insects was evaluated to ensure that phenotypes observed in adults after the meal, such as oviposition, were not reflections of a poor diet. We observed no significant difference in this aspect between insects injected with ds*GFP* or ds*RpCht7* (*p* > 0.05; one-way ANOVA–Tukey; Figure 10C).

**FIGURE 11:**
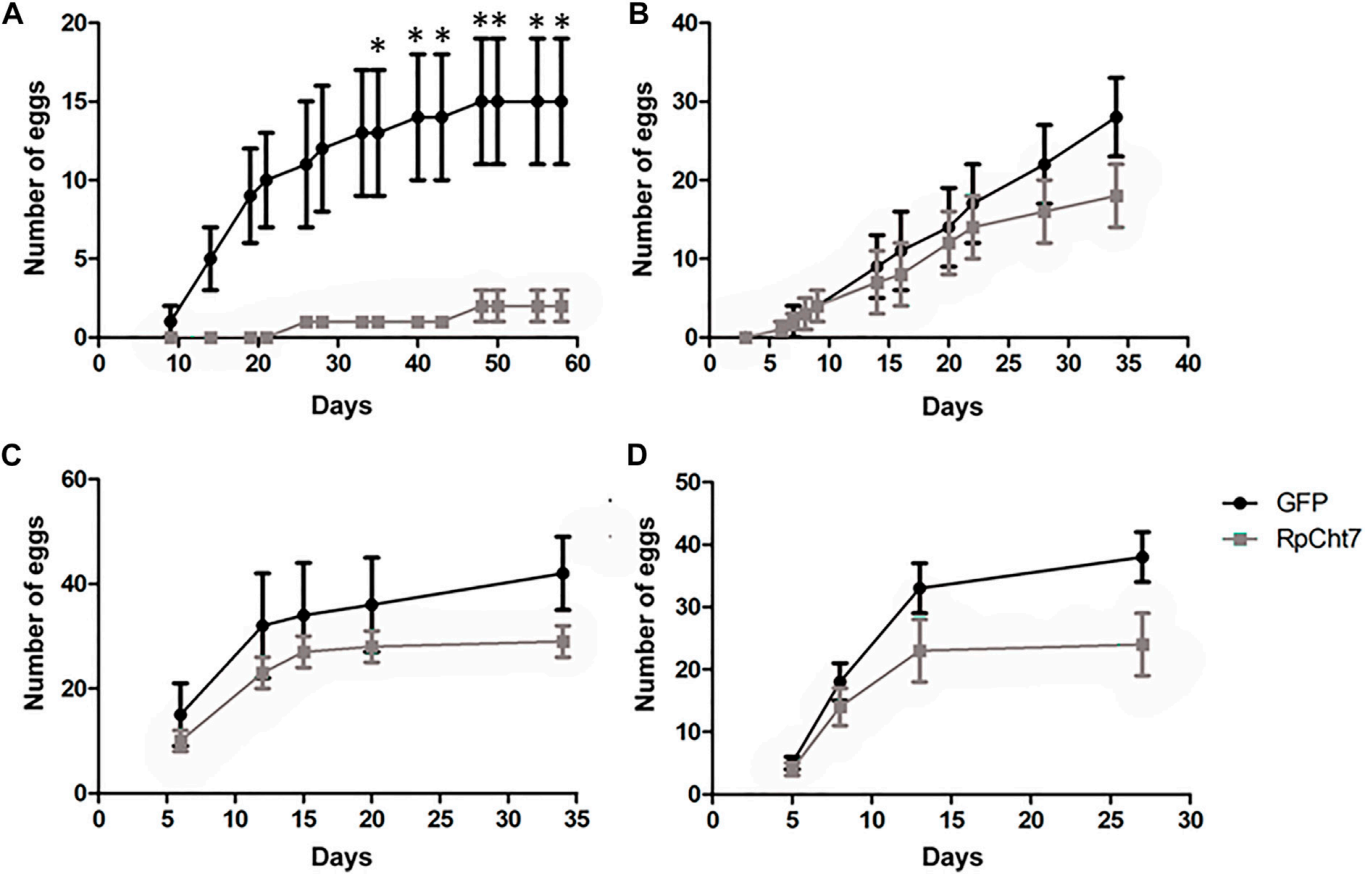
Average eggs laid per *R. prolixus* female at different times after blood feeding. In black, insects injected with ds*GFP*. In gray, insects injected with ds*RpCht7*. **(A–D)** are different experiment replicates. **(A)** 9 ds*GFP* females and 4 ds*RpCht*7 females; **(B)** 16 females in each group; **(C)** 7 ds*GFP* females and 13 ds*RpCht7* females; **(D)** 12 females ds*GFP* and 13 females ds*RpCht7*. *p < 0.05, unpaired T-test.

The first observed parameter related to oviposition was the average number of eggs laid per female. We verified oviposition kinetics by observing eggs laid per female across the days to analyze whether any group took longer to lay a certain number of eggs. However, the behavior of independent biological replicas varied considerably (Figures 11A–D). Despite that, in all experiments, females treated with ds*RpCht7* laid fewer eggs than controls treated with ds*GFP*. In the first experiment, differences were apparent beginning in the first week of observation. However, we observed significant differences only after the first month of observation due to the high variability between individuals (Figure 11A; *p* < 0.05; unpaired T-test). In the second and third experiments, differences were apparent only after 30 days but with no statistical significance (Figures 11B,C; *p* > 0.05, unpaired T-test). In the fourth experiment, the curves diverged after 13 days, but again with no statistical significance (Figure 11D; *p* > 0.05, unpaired T-test).

**FIGURE 12:**
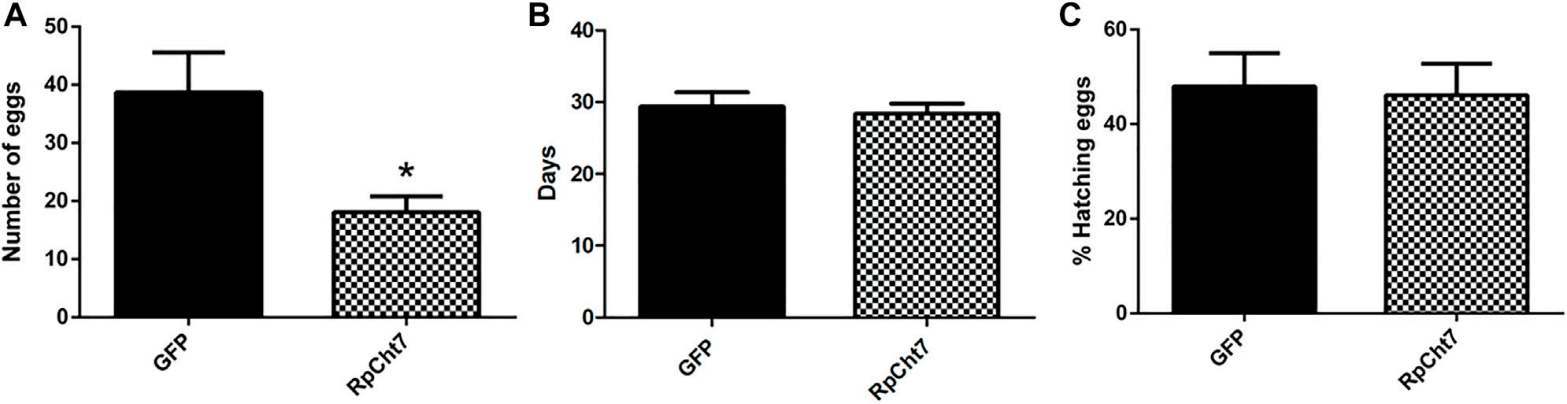
**(A)** Number of eggs per female after injection of dsRNA into fifth-instar nymphs of *R. prolixus*. The experiment was performed 5 times with 10–15 insects each. *p < 0.0001 (unpaired T-test). **(B)** Average hatching time of *R. prolixus* eggs. **(C)** Hatching percentage of *R. prolixus* eggs. The experiment was performed 4 times with 200–250 eggs each. No significant differences were found between *GFP* and *RpCht7* groups (*p* > 0.05; unpaired T-test).

Nevertheless, in an independent series of experiments, registering only the total numbers of eggs produced by each female after their death, we observed a highly significant and consistent decrease (*p* < 0.0005; unpaired T-test) in the number of eggs laid by females injected with ds*RpCht7* (average 39) when compared to females injected with ds*GFP* (average 18). We thus concluded that the knockdown of the *RpCht7* gene negatively affects oviposition (Figure 12A).

We also registered the average hatching time of eggs collected from females injected with ds*GFP* or ds*RpCht7*. The eggs took an average of 30 days after feeding to hatch in both the control and experimental groups. Thus, knockdown of the *RpCht7* gene did not influence egg hatching time (Figure 12B; *p* > 0.05; unpaired T-test).

We also observed the proportion of eggs that hatched successfully. From the total number of eggs laid in each group, we calculated the percentage of eggs hatched. Approximately 54% of eggs laid by the control group, and 58% of eggs laid by the experimental group, hatched. Therefore, there were no significant differences in the percentage of eggs hatched after silencing *RpCht7* (Figure 12C; *p* > 0.05; unpaired T-test).

## 4 Discussion

Analysis of the *R. prolixus* digestive tract transcriptome has identified 4 active chitinase genes (Ribeiro et al., 2014). The present work confirmed the existence of 9 predicted chitinase genes of the GH18 family in the *R. prolixus* genome (Mesquita et al., 2015). All transcripts found here showed high levels of similarity to other insect chitinases, with identities ranging from 19% to 69% in the amino acid sequences of the predicted proteins, suggesting that *R. prolixus* chitinase genes have homologs in other insect and invertebrate species.

Chitinase genes of the GH18 family present a modular structure comprising catalytic and chitin-binding domains joined by a highly glycosylated linker. These enzymes are classified from I to VIII based on similarities between their sequences and domain organization. In all insects studied so far, it has been observed that GH18 is a multigene family (ZHANG et al., 2011).

In five of the nine *R. prolixus* chitinase protein sequences (RpCht1, RpCht2, RpCht3, RpCht4, and RpCht8), it was possible to predict the presence of a signal peptide, indicating that these proteins may be secreted into the extracellular medium. Consistent with the Phobius IP data, the phylogenetic tree showed that RpCht1 belongs to group II of chitinases. Other genes in this group also have signal peptides, such as AgCht10, AaCht10, and TcCht10. RpCht2 and AaCht6 belong to group VI, with both presenting a putative signal peptide. RpCht4 belongs to group IV, and sequences AaCht12 and DmCht12 of this same group also have a signal peptide. RpCht8 did not show the conserved catalytic residues involved in hydrolysis and belongs to group V, as do AgIDGF2, AaIDGF1, and TcIDGF4, which also have signal peptides; it is probably an imaginal disc growth factor (IDGF), with a growth-promoting function, like the other members of this group.

*R. prolixus* chitinases have higher numbers of predicted O-linked glycosylations than N-linked glycosylations. While the number of N-linked glycosylations varies from 1 to 8, O-linked glycosylations range from 0 to 185; sequences that have at least one CBD (RpCht1, RpCht2, RpCht6, and RpCht9) are the ones with a greater number of O-glycosylations (185, 181, 30, and 37, respectively). These O-glycosylations are in a region between domains called a linker, which is a mucin-like domain (Steen et al., 1998). This mucin character gives elasticity to this region, being also responsible for the stability of chitinases against proteases and the correct folding of the whole proteins (Steen et al., 1998).

Gene expression analysis allowed us to observe the absence of an exclusive egg chitinase. Although *RpCht9* was significantly more expressed than the other genes in eggs, it is also highly expressed in the nymphal stages. *RpCht6* seemed exclusive to the nymphal stages, as this gene was not expressed in adult insects. As ecdysis does not occur in adults, this gene may be related to the ecdysone hormone responsible for molting (Wigglesworth, 1934). This is consistent with the fact that *RpCht6* belongs to group I of chitinases, whose function is associated with chitin hydrolysis during preparation for molting. The expression of this gene may be altered by insect growth regulator-type compounds such as azadiractin, triflubenzuron, and others (Henriques et al., 2016). No chitinase exclusive to adult males or females was observed. However, although not significantly different, we observed a higher expression of *RpCht1*, *RpCht5*, and *RpCht7* in males compared to females.

It was impossible to see the predominance of expression of any individual chitinase gene in most *R. prolixus* tissues. Ribeiro et al. (2014) found significant expression in the *R. prolixus* intestine of a group V chitinase, which corresponds to our *RpCht8*. Even though we did not obtain a significant difference in the *RpCht8* gene expression when compared to the other 8 chitinase sequences, we observed that the *RpCht8* gene is one of the most expressed genes, and we found significant differences (*p* < 0.05) when compared the expression of *RpCht8* with other genes that are less expressed in different tissues.

A study of chitinases in *Lutzomyia longipalpis* (Moraes et al., 2014) found different expression patterns throughout the developmental stages and in different tissues for some chitinase genes. The *LlChti2* gene belongs to the group VIII of chitinases, whose functional role is unclear, as does the *RpCht7* gene studied in this work. *LlChit2* did not show significant differences between the stages of development or tissues, which is consistent with the results found for *RpCht7*.

To confirm the function of chitinase genes, we chose the RNA interference technique, which is an essential tool for this kind of study. The RNAi technique has already been applied to different experimental models, such as *D. melanogaster* (Bellés, 2010), *Tribolium sp.* (Tomoyasu et al., 2008), *T. castaneum* (Tomoyasu and Denell, 2004; Arakane et al., 2008; Konopova and Jindra, 2008; Minakuchi et al., 2009), lepidopterans (Terenius et al., 2011; Mingbo, 2020), *R. prolixus* (Araujo et al., 2006), and others. As the inserting method of dsRNA, we chose microinjection, which afforded better control of the injected volume and caused smaller injuries (around 0.05 mm in diameter) than a conventional injection. Moreover, in *R. prolixus*, the effectiveness of silencing after injection (∼75%) is usually higher than with the feeding technique (∼42%) (Paim et al., 2013). RNAi effectiveness depends on the enzymatic degradation of dsRNA in the hemolymph or intestine (Wang et al., 2016). In *R. prolixus*, prolonged persistence of silencing effect and transmission to the next generation has already been proven when the injection is in the fifth-instar nymphs (Paim et al., 2013).

The knockdown success was highest against *RpCht3* and *RpCht7*, with a reduction of expression of approximately 80%. *RpCht2* gene was not silenced with the dsRNA fragment used, requiring targeting to another gene region. The reduction in gene expression for *RpCht9* and *RpCht8* was around 20% and 15%, respectively, which is not enough for phenotype analysis. For these genes, it would be interesting to confirm the silencing percentage by qPCR and, if it remains low, to redesign dsRNA primers as suggested for *RpCht2*. For *RpCht4* and *RpCht6*, we observed overexpression of 55% and 69%, respectively, 48 h after dsRNA injection. It is possible that after 24 h, these genes have been silenced and suffered regulation in response to silencing, resulting in overexpression. A similar response has already been observed in *Caenorhabditis elegans* (Liu et al., 2014).

We chose *RpCht7* for phenotype analysis due to the high mortality rate observed 48 h after injection in *RpCh7* knockdown insects. Furthermore, *RpCht7* belongs to the chitinase group VIII, which does not have a clear functional role described in the literature. So far, only one study has silenced a chitinase gene belonging to group VIII, the *NlCht2* gene in the hemipteran *Nilaparvata lugens* (Xi et al., 2015), finding that it was not possible to determine a clear functional role. *NlCht2* is expressed in all development stages, with slight changes, and is mainly expressed in adult female reproductive organs; its inhibition does not alter the insect’s morphology or survival.

Silencing of *RpCht7* was effective even at prolonged periods after injection, as observed for other *R. prolixus* genes (Paim et al., 2013). The first phenotype parameter evaluated was mortality in the fifth-instar nymphs of *R. prolixus* after injection with ds*RpCht7* or ds*GFP* for 15 days. In this period, we noticed a mortality rate about 2 times higher in insects injected with ds*RpCht7* when compared to control insects. A similar lethality phenotype has already been observed in lepidopterans *Mythimna separata* for the *MseCht1* and *MseCht2* genes (Cao et al., 2017), *Helicoverpa armigera* for the *HaChi* gene (Mamta et al., 2016), and in orthoptera *Locusta migratoria* for the *LmCht5-1* gene (Li et al., 2015). However, these phenotypes are related to reduced body weight, developmental deformities, and molt failure, respectively. None of these phenotypes were observed after *RpCht7* silencing. Thus, further studies are needed to understand how the suppression of the *RpCht7* gene affects the insect, leading to death.

Blood intake, diuresis, and digestion were evaluated using insect body weight; we did not observe differences between the weights of control and experimental insects. Thus, we can conclude that *RpCht7* gene function is not generally related to these functions. In any case, it would be interesting to quantify carbohydrates, proteins, and lipids in the intestinal contents throughout the digestion process in order to have a more detailed picture of these physiological functions. It would also be interesting to follow the production of perimicrovillar membranes and the morphology and histology of the midgut and its associated tracheas.

During the molt of fifth-instar nymphs to adults, we noticed that some insects were trapped in the exuvia and others were deformed, so we accounted for these defects in molting to determine if their cause was *RpCht7* silencing. Although the percentage of deformities was higher in insects treated with ds*RpCht7* (25%) than with ds*GFP* (20%), the difference was not significant. In addition, there was no delay in ecdysis time, and it was impossible to observe differences in the percentages of insects that changed from fifth nymphs to adults. In both groups injected with ds*RpCht7* or ds*GFP*, approximately 60% of insects became adults. Considering these three factors—time, percentage, and defects in ecdysis—we may deduce that the *RpCht7* function, at least in *R. prolixus*, is probably not related to ecdysis.

The sexual ratio was evaluated to check if *RpCht7* knockdown preferentially affected males or females. We obtained 45% females and 55% males in both groups, injected with ds*RpCht7* or ds*GFP*; we infer that the *RpCh7* gene does not affect the sex ratio. In addition, in observing oviposition kinetics, we noticed that egg number differences were significant only at 30 daf but with high variability between experimental replicas. Because of this low reproducibility in the temporal pattern of oviposition, we decided to analyze the total number of eggs laid per female to check if there was an effect in this parameter in the ds*RpCht7*-injected group as compared to ds*GFP* controls. The amount of blood ingested in both groups was the same, eliminating this factor as the cause of the difference in the number of eggs. We observed a significant 53% reduction in the number of eggs laid by ds*RpCht7*-injected insects compared to controls. This same phenotype of reduced number of laid eggs was observed in *Bx-chi-7* chitinase silencing in the nematode *Bursaphelenchus xylophilus* (Ju et al., 2016) and in *RpCHS* chitin synthase silencing in *R. prolixus* (Mansur et al., 2014). This reduction strongly suggests that *RpCht7* function is important for oviposition. However, its function is not related to egg hatching since the average hatching time was 30 days for both groups, and there was no difference in egg hatching percentage between groups.

Our results suggest that *RpCht7* plays an important role in *R. prolixus* reproductive physiology. However, additional studies are needed to further clarify the function of the *RpCht7* gene in *R. prolixus*. For example, it is crucial to assess in detail the effects of *RpCht7* gene silencing on egg formation, to observe if there are any morphological changes using electron microscopy, to count and measure oocytes, and to perform qPCR and enzymatic chitinase assays using ovaries and other organs to check if *RpCht7* gene activity is reduced differentially in any tissue. In this context, it would also be essential to verify the interaction of RpCht7 with vitellogenin synthesis, vitellogenin receptor production, endocytotic elements, or JH/ecdysone synthesis. Nevertheless, the knockdown of *RpCht7* also increases insect mortality before blood-feeding. In this respect, it would be interesting to assess the expression and physiological role of *RpCht7* in the posterior midgut and male reproductive organs, considering the high expression observed in this tissue and in male samples.

It is important to consider that, similarly to chitinases from family IV, RpCht7 has no chitin binding domain. This raises the possibility that this protein may not have hydrolytic activity, acting as a decoy for physiological or external chitinase inhibitors. However, chitinases from family IV, which are equally devoid of CBDs, have low activity against insoluble chitin but have high activity against oligo- or disaccharides, besides synthetic substrates. Some of them, specifically members of family IV, seem to be involved in the digestion of microorganisms. The absence of CBD may be related to avoiding the degradation of the insect’s peritrophic membrane (Genta et al., 2006). It is possible that RpCht7, despite not having a clear role in digestion, might work as well on chitin fragments or non-crystalline forms of the polysaccharide because it has all conserved regions and catalytic residues that are conserved in family 18 chitinases (Supplementary Figure S1G).

Chitin metabolism is considered a strategic target for the control of insects due to its physiological importance and the absence of this polymer in vertebrates (Wang et al., 2020). This perspective has been explored in the last decades, especially using chemical insecticides of the IGR class (Merzendorfer, 2013) (Ganguly et al., 2020). The inhibition of chitin synthesis, caused by compounds like triflumuron and lufenuron, results in dramatic mortality, especially during molt, in insects of different orders (Junquera et al., 2019). The effects of inhibitors of chitin synthesis were documented against *R. prolixus*, and, as expected, treatment of insects with these compounds resulted in an important reduction of viability of fifth-instar nymphs (Mello et al., 2008). Notably, the toxicity of triflumuron was recently studied in *R. prolixus* adults, showing that impairment of chitin metabolism profoundly affects oviposition and egg hatching (Henriques et al., 2016). Targeting chitin metabolism using specific tools like RNAi tends to diminish the impact of vector control on off-target insect species; in this context, it is essential to improve knowledge about chitinases in model insect vectors such as *R. prolixus*. In this work, we described and characterized the multigenic group of chitinases of GH18 in this insect and performed knockdown studies for most of them, focusing on a member (*RpCht7*) of the less-known subgroup VIII. These studies may create the requisites needed for a better understanding of the role of chitinases in the physiology of triatomine vectors.

## Data Availability

The datasets presented in this study can be found in online repositories. The names of the repository/repositories and accession number(s) can be found in the article/Supplementary Material.

## Supplementary material

SUPPLEMENTARY FIGURE S1
**(A)**: Amino acid sequence alignment of *R. prolixus* protein RpCht1 and selected homologous chitinases from GH18. The signal peptide is boxed in black. Conserved residues at the active site are detached in the red box. Sequences used in alignment come from *Monochamus alternatus* (MaCht1: BAG13448.1 and MaCht2: BAG13449.1), *Tribolium castaneum* (TcCht6: EFA10488.1 and TcCht10: NP_001036067.1), and *Tenebrio molitor* (TmCht1: CAD31740.4). **(B)**: Amino acid sequence alignment of *R. prolixus* protein RpCht2 and selected homologous chitinases from GH18. The signal peptide is boxed in black. Conserved residues at the active site are detached in the red box. Sequences used in alignment come from *Acyrthosiphon pisum* (AcypiCht1: XP_001942596.2), *Dendroctonus ponderosae* (DpCht1: ENN78373.1), *T. castaneum* (TcCht1: EFA00965.1), *Drosophila grimshawi* (DgCht1: XP_001991892.1), and *Drosophila ananassae* (DaCht1: XP_001966685.1). **(C)**: Amino acid sequence alignment of *R. prolixus* protein RpCht3 and selected homologous chitinases from GH18. The signal peptide is boxed in black. Conserved residues at the active site are detached in the red box. Sequences used in alignment come from *Bombyx mori* (BmCht3: XP_004922005.1 and BmCht1: XP_004922004.1), *Daphnia pulex* (DpCht1: EFX88295.1), *Ceratitis capitata* (CcCht1: XP_004524466.1), and *Anopheles gambiae* (AgCht1: XP_308858.4 - EAA04006.4). **(D)**: Amino acid sequence alignment of *R. prolixus* protein RpCht4 and selected homologous chitinases from GH18. The signal peptide is boxed in black. Conserved residues at the active site are detached in the red box. Sequences used in alignment come from *Dendroctonus ponderosae* (DpCht3: ENN72974.1 and DpCht4: ERL92170.1), *Tribolium castaneum* (TcCht3: XP_970191.2 - EFA06923.1), *Pediculus humanus corporis* (PhCht1: XP_002423089.1–EEB10351.1), and *Megachile rotundata* (MrCht2: XP_003706336.1). **(E)**: Amino acid sequence alignment of *R. prolixus* protein RpCht5 and selected homologous chitinases from GH18. The signal peptide is boxed in black. Conserved residues at the active site are detached in the red box. Sequences used in alignment come from *Dendroctonus ponderosae* (DpCht3: ENN72974.1 and DpCht4: ERL92170.1), *T. castaneum* (TcCht3: XP_970191.2 - EFA06923.1), *Bombyx mori* (BmCht2: XP_004933352.1), and *P. humanus corporis* (PhCht1: XP_002423089.1–EEB10351.1). **(F)**: Amino acid sequence alignment of *R. prolixus* protein RpCht6 and selected homologous chitinases from GH18. The signal peptide is boxed in black. Conserved residues at the active site are detached in the red box. Sequences used in alignment come from *Dendroctonus ponderosae* (DpCht2: ENN79462.1), *Tribolium castaneum* (TcCht2: EFA10939.1 and TcCht5: NP_001034524.1), *Poophilus costalis* (PcCht1: AFW03959.1), and *Nasonia vitripennis* (NvCht1: NP_001155084.1). **(G)**: Amino acid sequence alignment of *R. prolixus* protein RpCht7 and selected homologous chitinases from GH18. The signal peptide is boxed in black. Conserved residues at the active site are detached in the red box. Sequences used in alignment come from *Apis melífera* (AmCht1: XP_006572082.1), *Apis dorsata* (AdCht1: XP_006624796.1), *Bombus impatiens* (BiCht1: XP_003488707.1), *Cerapachys biroi* (CbCht1: EZA54975.1), and *Bombus terrestres* (BtCht1: XP_003395312.1). **(H)**: Amino acid sequence alignment of *R. prolixus* protein RpCht8 and selected homologous chitinases from GH18. The signal peptide is boxed in black. Conserved residues at the active site are detached in the red box. Sequences used in alignment come from *Oncometopia nigricans* (OnCht1: AAU95190.1), *Mamestra brassicae* (MbCht1: ABC79625.1), *Megachile rotundata* (MrCht1: XP_003702512.1), and *Apis melífera* (AmCht2: XP_006566882.1, and AmCht3: XP_006566881.1). **(I)**: Amino acid alignment of *R. prolixus* protein RpCht9 and selected homologous chitinases from GH18. The signal peptide is boxed in black. Conserved residues at the active site are detached in the red box. Sequences used in alignment come from *Plutella xylostella* (PxCht1: AFI55112.1), *Acyrthosiphon pisum* (AcypiCht2: XP_001950380.1), *Ostrinia furnacalis* (OfCht1: AGX32025.1) e *Bombyx mori* (BmCht1: XP_004922004.1 and BmCht3: XP_004922005.1).

SUPPLEMENTARY TABLE S1 Oligonucleotide primers sequences used for semi-quantitative RT-PCR and synthesis of dsRNA of transcripts encoding the GH18 family in *R. prolixus* and Actin, the constitutive gene used. For the synthesis of dsRNA, each specific sequence was coupled to the T7 promoter sequence (23bp) according to manufacturer instructions (RiboMAX express T7 RNAi system, Promega, USA). Forward: Sense initiator. Reverse: Antisense initiator. Bp: base pairs.

SUPPLEMENTARY TABLE S2 Summary of the characteristics of genes coding proteins belonging to the family 18 of glycoside hydrolases in the genome of *R. prolixus*. Vector base: code of vector base from chitinase genes. Signal IP 4.1 e Signal IP 3.0: signal peptides. Phobius: transmembrane topology and signal peptides pI: Isoelectric point. MW: Molecular weight. N-Glyc: number of N-linked glycosylations. O-Glyc: number of O-linked glycosylations. GPI: Identification of GPI anchor signals. Hmmtop: location of helical transmembrane segments.

SUPPLEMENTARY TABLE S3 Comparison of exon number of *R. prolixus* sequences with *Anopheles gambiae*, *Aedes aegypti*, and *Pediculus humanus* sequences of each group of the phylogenetic tree. RpCht: *Rhodnius prolixus.* PhCht: *Pediculus humanus.* AgCht: *Anopheles gambiae.* AaCht: *Aedes aegypti.*

SUPPLEMENTARY TABLE S4 Comparison of chitinase predicted proteins’ molecular masses with the average/range of chitinase molecular masses in the groups from phylogenetic trees. RpCht: Name of *R. prolixus* chitinases. MW (Kda) RpCht: Molecular mass of *R. prolixus* chitinases. Group: Number of chitinase groups in the phylogenetic tree. MW (Kda) Group: Molecular mass average of chitinases group in the phylogenetic tree.
