# Supplementary material for: Structure and expression of Rhodnius prolixus GH18 chitinases and chitinase-like proteins: Characterization of the physiological role of RpCht7, a gene from subgroup VIII, in vector fitness and reproduction
Source: Front Physiol. 2022 Oct 3;13:861620. doi: 10.3389/fphys.2022.861620 (PMC9574080; doi:10.3389/fphys.2022.861620)
Supplement: Supplementary file 1 [file Table1.DOCX]

|  | **RT-PCR** | | | **dsRNA** | | |
| --- | --- | --- | --- | --- | --- | --- |
| **Gene** | **Forward (5’-3’)** | **Reverse (5’-3’)** | **Amplicon size, pb** | **Forward (5’-3’)** | **Reverse (5’-3’)** | **Amplicon size, pb** |
| **Actin** | CCAGGTATTGCTGACAGGATGCAA | ATGCCGGTCCAGATTTCGTCGTAT | 200 | - | - | - |
| **RpCht1** | TCAGCCCAAGAATCGTGTG | CGCGGGTTGTAAAACTGATC | 300 | GAAGGAAAGTAGTTTTGGGAATGCC | CTTCATCTTTAACTACTGTCCATCCT | 195 |
| **RpCht2** | AAGGACAAGTGAACTGATGGG | GAGGCATTATGATTTGGTCGATTG | 400 | CAAACAAACTTGTGTTGGGCATACC | TGTTCTTTGGATGTACTACAGTCCAG | 195 |
| **RpCht3** | TGCTAACGATATGTCTGCCATC | GCGCTAATTTCTTGTTGTAGAGG | 200 | GTGATAAATTAAATCTTGGTATTCCAAC | TTTCATTTACATGTACTACAGTCCAGT | 201 |
| **RpCht4** | AAGAACAAGACTGAACGAGGAC | TGTAAAGCTGTGTCCGAACG | 300 | ATACCAACGTTCGGACACAGCTTTA | CGTCATCCCACATTTCTTTCCAACC | 181 |
| **RpCht5** | AAACGTTCACCCGCAGCGTAATGA | ATGGTGTGGGTTTGGTACCTCGTT | 700 | GTGCCCATGTATGGACGTACCTTC | CTAATATGGAAATCTATACAAATGGTGT | 198 |
| **RpCht6** | AGCGACCGCATGACCAATATGCTT | TTGGCGTTGTAGTCACAGTTGGCA | 600 | CTGACAAGCTGGTTGTTGGAGTTC | CATCATCCCACTTTTGTACCCATTG | 213 |
| **RpCht7** | AACGCAGGGAGGCCTTTAAGGATGTT | TAAAGAGGCGCGTTTGGTCCAGTT | 600 | GGACTAAAATCAATGTTGGCATCCC | CTTCATCAAATACATGGGTAGTACTTG | 186 |
| **RpCht8** | ACACAAAGACCAGTTTGTGGCGCT | TGCCAAGATTCAGTTTCTGGCCGT | 500 | GCCAGAAACTGAATCTTGGCATTGC | TACCAGCTGGTGCGCTAGGGTTG | 201 |
| **RpCht9** | AGCTGAAAGCGGCTTCATGGCATA | TGCGTGTGTTGCGAACATCCTTCT | 500 | AAGAGAAACTTATGATTGGTATGCCAA | CATTATCCCAAACCAAAGTAGTGTTAT | 195 |
