# Supplementary material for: Structure and expression of Rhodnius prolixus GH18 chitinases and chitinase-like proteins: Characterization of the physiological role of RpCht7, a gene from subgroup VIII, in vector fitness and reproduction
Source: Front Physiol. 2022 Oct 3;13:861620. doi: 10.3389/fphys.2022.861620 (PMC9574080; doi:10.3389/fphys.2022.861620)
Supplement: Supplementary file 2 [file Table2.DOCX]

| **NAME** | **Vector Base** | **Signal IP 4.1** | **Signal IP 3.0** | **Phobius IP** | **pI** | **MW** | **N-GLYC** | **O-GLYC** | **GPI** | **Hmmtop** |
| --- | --- | --- | --- | --- | --- | --- | --- | --- | --- | --- |
| **RpCht1** | RPRC015248 | YES 16/17 | YES 16/17 | YES 1/19 | 6.53 | 297148.57 | 6 (230/ 476/ 1129/ 1540/ 1973/ 2474) | 185 | NO | 1 (50-70) |
| **RpCht2** | RPRC005523 | NO | YES 24/25 ou 16/17 | YES 1/19 | 8.84 | 137811.19 | 2 (46/925) | 181 | NO | 1(532-550) |
| **RpCht3** | RPRC011446 | YES 19/20 | YES 19/20 | YES 1/21 | 5.60 | 44678.27 | 8 (151/227 /298/305/ 384/586/ 728/735) | 12 | NO | 1 (16-32) |
| **RpCht4** | RPRC014089 | YES 22/23 | YES 16/17 ou 22/23 | YES 1/22 | 6.84 | 48748.75 | 1 (66) | 2 | NO | 1 (92-109) |
| **RpCht5** | RPRC014092 | NO | NO | non cytoplasmic  (without signal peptides) | 5.84 | 38110.09 | 3 (51/103/ 309) | 5 | NO | 1 (22-46) |
| **RpCht6** | RPRC012296 | NO | NO | non cytoplasmic  (without signal peptides) | 5.25 | 64484.51 | 2 (69/90) | 30 | NO | 1 (36-53) |
| **RpCht7** | RPRC012021 | NO | YES 24/25 | Transmembrane domain -  extracellular protein | 5.83 | 50239.03 | 6 (16/45/ 109/135/ 226/291) | 0 | NO | 1 (19-43) |
| **RpCht8** | RPRC003338 | YES 19-20 | YES  19-20 | Yes 1/19 | 6.77 | 48159.91 | 1 (215) | 6 | NO | 0 |
| **RpCht9** | RPRC014976 | NO | NO | Transmembrane domain - extracellular protein | 6.35 | 117743.31 | 5 (54/59/165/832/85) | 37 | NO | 1 (93-110) |
