# Supplementary material for: Structure and expression of Rhodnius prolixus GH18 chitinases and chitinase-like proteins: Characterization of the physiological role of RpCht7, a gene from subgroup VIII, in vector fitness and reproduction
Source: Front Physiol. 2022 Oct 3;13:861620. doi: 10.3389/fphys.2022.861620 (PMC9574080; doi:10.3389/fphys.2022.861620)
Supplement: Supplementary file 3 [file DataSheet1.PDF]

A

```
RpCht1     1 -----MWLLALFVILLTLAHG---------------------------FRRDSVESPP---TLLTRSSAYNLP--FGWKSLPLRTAVEKVPTFRERSQEKS 64
MaCht1     1 MELRLTICAVLTFYASLTNEKVLRVKRSE---------HVPPLDAPTFRRDSVEHPPIFDRYTDRSSYYTVLEEYGSRSLPLRAAVESIPDVIKTSK-RL 90
TcCht6     1 MELLHITLCAFLILTPTDQRVVTR-------------------TIPSFLRDDVERYPSGPELSDRSAAYTNRELYGTRALPLRSAVEHIPDLIADSR-RL 80
TcCht10    0 ---------------------------------------------------------------------------------------------------- 0
TmCht1     1 MELLHLTLCALLVLLTPTEERVTRRHVPEEGSTRTKRAHIPPLSSPSFRRDAVESPPEVSEISDRHAAYTNRDQYGTRSLPLRTAVERVPDLIEDSI-RV 99
MaCht2     1 MELRLTICAVLTFYASLTNEKVLRVKRSE---------HVPPLDAPTFRRDSVEHPPIFDRYTDRSSYYTVLEEYGSRSLPLRAAVESIPDVIKTSK-RL 90
Clustal

RpCht1    65 PLRAAVERRP------------------------------------------------------------------------------------------ 74
MaCht1    91 PLRDAVEKRPPPDEPLDAVEDLDETNYNVLSYQKINPSGKLDFSSTSENAPHHDIPKTKPNFNSYSIPQYQHVPPQYVPNQYLYTPHNFVYSGPQPHFYQ 190
TcCht6    81 PLRDAVEKRPPPDEP---IDDYEPS------------------------------------------------------FDLFYYPS------------- 110
TcCht10    1 PLRDAVEKRPPPDEP---IDDYEPS------------------------------------------------------FDLFYYPS------------- 30
TmCht1   100 PLRDAVEKRPPPDEPD-DVDDFELDNN---------------------------------------------------EYQFYYYPP------------- 134
MaCht2   134 PLRDAVEKRPPPDEPLDAVEDLDETNYNVLSYQKINPSGKLDFSSTSENAPHHDIPKTKPNFNSYSIPQYQHVPPQYVPNQYLYTPHNFVYSGPQPHFYQ 190
Clustal   91 *** ***:**

RpCht1    75 --TPYFRGPWWRLVPGTKQSD----------------------------------------------HTGKRLFCFVESWASYRRSPAAYTADNIDPFAC 126
MaCht1   191 SQTLYSPLQFFRNVIGDYRSATPYKIDNSFQQLLVSPAVPRGRSLEPEHPFLRRGS----EQAGNNDAANKKIVCYVEGKATYRKEPLNFSPEELDAFAC 286
TcCht6   111 RQTLYTPVQYFKDLVAEAYVS-PFRIDNESQELLKYPSFARGRSLYDS----RGRHSEIVETVYDPRED-DRVVCYVQAAARYRKEPLAFSPEDLDPFAC 204
TcCht10   31 RQTLYTPVQYFKDLVAEAYVS-PFRIDNESQELLKYPSFARGRSLYDS----RGRHSEIVETVYDPRED-DRVVCYVQAAARYRKEPLAFSPEDLDPFAC 124
TmCht1   135 RVSLYTPIQYFRDLISEQYVSVPYRIDNVSQQLIRYP--ARGRSLFGDNPFLRGRHSEIVETVYDPREEQDRVVCYVQAAATYRKEPLSFSPEDLDPYAC 232
MaCht2   191 SQTLYSPLQFFRNVIGDYRSATPYKIDNSFQQLLVSPAVPRGRSLEPEHPFLRRGS----EQAGNNDAANKKIVCYVEGKATYRKEPLNFSPEELDAFAC 286
Clustal        : *    ::: : .                                                      .::.*:*:. * **:.*  ::.:::*.:**

RpCht1   127 THLIYAFVTIDQASSKIMPLDEEYDVVKGGYRSVVGLKRVNSNLKVLVSL-------SGRLSKMTSTASNRRKFILSTVKFLSENGFDGIDIHWEYPGAE 219
MaCht1   287 THVIYAYASIDPHTFEMISNDEEFDIVQGGYRAITGLKRLNPNLKVLISLGGGREDGSHRFSSLISSARHRPDFIRSAISFLKQYDFDGMDVHWQYPGAE 386
TcCht6   205 THVIYAFATIDPHNFNMISNDDESDIIQGGYISVTGLKRVNPKLKVLISVGEGR-DGSHRFSTMVSSANRRREFIRSAITFIKQYNFDGMDIHWEYPGAE 303
TcCht10  125 THVIYAFATIDPHNFNMISNDDESDIIQGGYISVTGLKRVNPKLKVLISVGEGR-DGSHRFSTMVSSANRRREFIRSAITFIKQYNFDGMDIHWEYPGAE 223
TmCht1   233 THVIYAFASIDPHTFNMISNDDEFDIIQGGYRSVTGLKRLNPKLKVLISVGEGRQDGSHRFSSMVSSANRRREFIRSAIGLIKQYNFDGMDIHWEYPGAE 332
MaCht2   287 THVIYAYASIDPHTFEMISNDEEFDIVQGGYRAITGLKRLNPNLKVLISLGGGREDGSHRFSSLISSARHRPDFIRSAISFLKQYDFDGMDVHWQYPGAE 386
Clustal      **:***:.:**  . :::. *:* *:::*** ::.****:*.:****:*:       * *:*.: *:* .* .** *:: ::.: .***:*:**:*****

RpCht1   220 ELGGKENDKENLSKLIEEMSGVLDSRGWLLTASVSPSRFRLEDGYDVPRIAPYLHFILLKSFDFQLERDNAANHPSPLIMSAQEDPLSLYYNVDYAVKFW 319
MaCht1   387 ELGGHVTDKEFLTLFLEEVSEIFKERGWLLVVSAPASRFRIEDGFNPTSLGSVVDFVNVQAYNFHRDREPVADHHSNLYARPQDNGLDLFLSVDYAVKFW 486
TcCht6   304 KMGGQLSDKEYLNLFLEELSEIFKPRGWVLTIAVPASRFRVEDGFNPQRLGSLVDFINLQAFDFHREREPVADHHANLYSRPGDSGLDLFLSVDYAVKFW 403
TcCht10  224 KMGGQLSDKEYLNLFLEELSEIFKPRGWVLTIAVPASRFRVEDGFNPQRLGSLVDFINLQAFDFHREREPVADHHANLYSRPGDSGLDLFLSVDYAVKFW 323
TmCht1   333 ELGGQLSDKEYLNLFLEELSEIFKPRGWLLSIAVPASRFRVEDGFNPQRLGSLVDFVNLQAFDFHRERDPVADHHSNLYPRPHDNGLDLFLSVDYAVKFW 432
MaCht2   387 ELGGHVTDKEFLTLFLEEVSEIFKERGWLLVVSAPASRFRIEDGFNPTSLGSVVDFVNVQAYNFHRDREPVADHHSNLYARPQDNGLDLFLSVDYAVKFW 486
Clustal      ::**: .*** *. ::**:* ::. ***:*  :...****:***::   :.. :.*: :::::*: :*: .*:* : *   . :. *.*: .********

RpCht1   320 LKRGVHRSQLILGIPFFGRSYTLRDEKKWTPGSPIKSVGEEARFTQQPGFMAYYEVCTRMKRKEWLKHRDASGSAYMVYKDQWVGFEDVYSIQLKMQYLK 419
MaCht1   487 LKKGLPRSKLVLGIPFFGRSFTLQYSNDTNLGSPIKGPGREGFYTQNPGFLGYFEICELELNEGWYRYEDAVGSPYLVNGNQWVGFDDERSIRKKIEYVK 586
TcCht6   404 TKKGFPKSKIVLGVPFFGRSFTLQFTNETQVGAPIKGPGREGFYTQNPGFLAYFEICDMLLNEGWHKGMDESGSPYMVYGDQWVGYDDSESVEKKVNYVQ 503
TcCht10  324 TKKGFPKSKIVLGVPFFGRSFTLQFTNETQVGAPIKGPGREGFYTQNPGFLAYFEICDMLLNEGWHKGMDESGSPYMVYGDQWVGYDDSESVEKKVNYVQ 423
TmCht1   433 IKKGLPKSKIIVGIPFFGRSFTLQYSNETQEGAAIKGPGREGFYTQNPGYLAYFEICDMLLNEGWHKGVDEAGAPYMVYGDQWVGYDDAESVERKVNYVQ 532
MaCht2   487 LKKGLPRSKLVLGIPFFGRSFTLQYSNDTNLGSPIKGPGREGFYTQNPGFLGYFEICELELNEGWYRYEDAVGSPYLVNGNQWVGFDDERSIRKKIEYVK 586
Clustal       *:*. :*::::*:******:**:  :.   *:.**. *.*. :**:**::.*:*:*    .: * :  *  *:.*:*  :****::*  *:. *::*::

RpCht1   420 QEQLGGVMVWSLDLDDFQGTCGTKYPLLTTIKNSLAPEIPAVISPGVGVTVLASSSNCTGEGYVGDTLDCSVYYRCQWSMKHTYICPHGLFYDPVLNLCN 519
MaCht1   587 VQDLAGVYVFAADLDDFKGLCGEKWPLLTALKKDLRGGEQSSINFDHNPPTQPFG-TCQSTGFYSDSKNCAAYYVCKNGLSYHLSCGENLMFNSNTAKCE 685
TcCht6   504 EMNLGGVFIWATDLDDFKGVCGMKWPLLSTINRHLRGNELLPMQQSQNPPTKPYG-TCRSEGIFSDPKNCAAYYVCRSGLSYHLSCAENMMFDPMSGKCE 602
TcCht10  424 EMNLGGVFIWATDLDDFKGVCGMKWPLLSTINRHLRGNELLPMQQSQNPPTKPYG-TCRSEGIFSDPKNCAAYYVCRSGLSYHLSCAENMMFDPMSGKCE 522
TmCht1   533 EMNLGGVFVWAADLDDFKGVCGVKWPLLSTINRHLRGNELLPLQQHTDAPTKPYG-TCRSEGLFTDPRNCAAYYICRSGLSYHLSCADNMMFDPANGRCE 631
MaCht2   587 VQDLAGVYVFAADLDDFKGLCGEKWPLLTALKKDLRGGEQSSINFDHNPPTQPFG-TCQSTGFYSDSKNCAAYYVCKNGLSYHLSCGENLMFNSNTAKCE 685
Clustal        :*.** ::: *****:* ** *:***::::. *       :.   . .. . . .* . *   *. :*:.** *: .:.:   * ..::::.      *:
```

```
RpCht1    520 WPTLVTC-PVQSKMMYVWEFYFKCILYSESDKDGK-KVVCYYTNWAWYRKGEAKFFPEHINPDLCTHVIYAFASLEPNSLTINAFDPWADLENSMYTRVT 617
MaCht1    686 HFSPERCKPGQTVYIPNSLKEIDPLLRKDQIKDSKPKVVCYVTNWAFYRKAEGKFVPEHIDQRLCTHVVYAFASLDPEKLLMKEFDPWADLDNSLYERIT 785
TcCht6    603 YSLGEKCRPGQIIQVANSLRQYEDLLEYEA-KEEGPKVVCYMTNWAFYRKAEGKFVPEHIDQRLCTHVVYAFASLDPEKLLLKEFDPWADLDNNLYERVT 701
TcCht10   523 YSLGEKCRPGQIIQVANSLRQYEDLLEYEA-KEEGPKVVCYMTNWAFYRKAEGKFVPEHIDQRLCTHVVYAFASLDPEKLLLKEFDPWADLDNNLYERVT 621
MaCht2    686 HFSPERCKPGQTVYIPNSLKEIDPLLRKDQIKDSKPKVVCYVTNWAFYRKAEGKFVPEHIDQRLCTHVVYAFASLDPEKLLMKEFDPWADLDNSLYERIT 785
TmCht1    632 FSLGEKCRPGQIMQVPNSLRLFDHLLKAEAPKDDRPKVVCYMTNWAFYRKGEGKFVPEHIDQRLCTHVIYAFASLDPDKLLLKEFDPWADLDNNLYERVT 731
Clustal             * * *   :       . :*  :  *:   ***** ****:***.*.**.****:  *****:******:*:.* :: *******:*.:* *:*

RpCht1    618 NLRLKGVAVLLAIGGWTDSTGDKYSKLVSSGSNRRVFVGSVVAFLRKYKESGLSLEWNYPVCWQSDCKKGPSSDKANFAKLVE-----ELRDAFDKETPT 712
MaCht1    786 --SLPDTTTLLSLGGWTDSSGDKYSRLVSDGSARRRFVIGAVGFLRRYGFKGLHFDWNYPVCWQSNCKRGPSSDKTNFPKLLQ-----ELRKEFDKENPP 878
TcCht6    702 --SLKDTKAILSLGGWTDSAGDKYSRLVGDGSARRRFVVAVVGFLRRHNFKGLHLDWNYPVCWQSNCKKGASSDKPNFTKLIQ-----ELRREFDKQKPP 794
TcCht10   622 --SLKDTKAILSLGGWTDSAGDKYSRLVGDGSARRRFVVAVVGFLRRHNFKGLHLDWNYPVCWQSNCKKGASSDKPNFTKLIQVVPTMELRREFDKQKPP 719
TmCht1    732 --SFQDTKVLLSLGGWTDSAGDKYSRLVGDGSARRRFVIGVVSFLRRHHFRGLHFDWNYPVCWQSNCKKGASSDKPNFTKLIQ-----ELRREFDKQKPP 824
MaCht2    786 --SLPDTTTLLSLGGWTDSSGDKYSRLVSDGSARRRFVIGAVGFLRRYGFKGLHFDWNYPVCWQSNCKRGPSSDKTNFPKLLQ-----ELRKEFDKENPP 878
Clustal               : .. .:*::******:*****:**..** ** **  ..*.***:: * ** ::********:**:*.****.**.**::     ***  ***:.*.

RpCht1    713 LMLSVSISGYSEIIDASYDLPAISKAVDFMTFMSYDYHGAWEGVTGHVSPLRGKSSDKFALYTTSAATEYLISKGADKKKIILGIPFYGQTFTLKSPKEN 812
MaCht1    879 LVLAASISGYKEVIDVAYDLTTLGQTLDFISVMTYDYHGAWEHQTGHVSPLYQRPSDKYPQYNSNFTMEYLVSRGAPREKLLMGVPFYGQSFTLTKDTNY 978
TcCht6    795 LILAAAISGYKEVIDVAYDLPALGTVLDFMSVMTYDYHGAWERQTGHVSPLFHRPGDKYPQYNTNFTMEYLVSRGAPRGKLLVGVPFYGQSFTLTKGNNH 894
TcCht10   720 LILAAAISGYKEVIDVAYDLPALGTVLDFMSVMTYDYHGAWERQTGHN-----------------FTMEYLVSRGAPRGKLLVGVPFYGQSFTLTKGNNH 802
TmCht1    825 MILAAAISGYKEVIDVAYDLQSLGGALDFMSVMTYDYHGAWERQTGHVSPLYHRQGDKYPQYNTNYTMEYLVAKGAPREKLLVGIPFYGQSFTLSKTNNH 924
MaCht2    879 LVLAASISGYKEVIDVAYDLTTLGQTLDFISVMTYDYHGAWEHQTGHVSPLYQRPSDKYPQYNSNFTMEYLVSRGAPREKLLMGVPFYGQSFTLTKDTNY 978
Clustal           ::*:.:****.*:**.:*** ::. .:**::.*:********  ***                          : ***:::** : *:::*:*****:***.. .:

RpCht1    813 TIGSDVTGPGNPGPYTKQPGMAAYYEICYRIKKHLWR---KENSPYGPYAYYGDQWVSFDDQKSIKEKADYVKTNELGGIMAWTTDLDDFQNMCCEGQFP 909
MaCht1    979 GENAPSSGPGEPGEYTKQPGMLAYYEVCNRIRNQRWISNSNSASG-GPYAYNRNQWVGYEDVNSIKEKANYIKTKGFGGAVAWTIDLDDFNNRCCEGTFP 1077
TcCht6    895 DQGVPSAGPGEAGEYTKQPGMLAYYEICNRIRNQRWIVNRDTSGATGPFAYHGDQWVGFEDIKSVRDKAEYIKTKGFGGAVAWTIDLDDFSNRCCGGTFP 994
TcCht10   803 DQGVPSAGPGEAGEYTKQPGMLAYYEICNRIRNQRWIVNRDTSGATGPFAYHGDQWVGFEDIKSVRDKAEYIKTKGFGGAVAWTIDLDDFSNRCCGGSFP 902
TmCht1    925 GQGVPSAGPGEAGEYTKQPGMLAYYEICARIRNQRWIVTPDSTGATGPFAYHGEQWVGFEDVTSVRKKASYIKSSGFGGAVAWTIDLDDFSNRCCGGSFP 1024
MaCht2    979 GENAPSSGPGEPGEYTKQPGMLAYYEVCNRIRNQRWISNSNSASG-GPYAYNRNQWVGYEDVNSIKEKANYIKTKGFGGAVAWTIDLDDFNNRCCEGTFP 1077
Clustal             .    :***:.* ******* ****:* **::: *     .  .  **:**  :***.::* .*::.**.*:*:. :** :***  *****.* ** * **

RpCht1    910 LLFAINSALGRDVKQTVSG-CNKPPTPVTPPPVITTTLAPDSGPTT--TEHEHVHWTEPTTLKTTTTRRPTTT--------------------------- 979
MaCht1  10778 LLRSLNYELGLITDKPTKGDCTKPPAPSTPAPPETTTGVDSGAASSSTTEHIHGEWTSSTSKKPTTSAWWTTTKKPTSSKPTTSPWWTNKPSSSSSSKPST 1177
TcCht6    995 LLRSLNRGLGLIPDNPSREDCTKPPEPVTPAPPQVTTGVDTGASS--TEHMHPDWT----TKPST-WWSSTTT--------------------------- 1060
TcCht10   903 LLRSLNRGLGLIPDNPSREDCTKPPEPVTPAPPQVTTGVDTGASS--TEHMHPDWT----TKPST-WWSSTTT--------------------------- 968
TmCht1   1025 LLRSLNRGLGLIPDTPSREDCTKPPEPVTPAPPQTTTGVDTGASS--TEHMHPEWTKPT-TKPTTSSWWSTTT--------------------------- 1094
MaCht2   1077 LLRSLNYELGLITDKPTKGDCTKPPAPSTPAPPETTTGVDSGAASSSTTEHIHGEWTSSTSKKPTTSAWWTTTKKPTSSKPTTSPWWTNKPSSSSSSKPST 1177
Clustal           ** ::*  **   . .     *.*** * **.*   .* . *:*.::  *** * .**      *.:*     :**.

RpCht1    980 ------TTTTTRRPT------------------TTTTTTTTRRPTTTRKP-----TTTTTTEQSNVG--------ETTDLPSTCQTGEYRPVKGNCNAYY 1042
MaCht1   1178 TWSPWWSTSSSSKPTTKPSTPVVWWTEKPSTIPTTRPTTTTVAWWATKPSTTAKPTTETTPDGSTIVPPPAVVMPEVDTPSKPCEPGQHLPDATNCNAYY 1277
TcCht6   1061 --SPWWTTTTTRRTT------------------TTRPTTTS---TTTRP------TTTNWPTQGTTIPPPAVVMPEVDKPSQPCEPGQYVPDPHNCNAYY 1134
TcCht10   969 --SPWWTTTTTRRTT------------------TTRPTTTS---TTTRP------TTTNWPTQGTTIPPPAVVMPEVDKPSQPCEPGQYVPDPHNCNAYY 1039
TmCht1   1095 --SPWWTTTTTRRTT------------------TTRRTTTTRRTTTTRPST----TTTSWPTHATTIPPPAVVMPEVDKPPQPCEPGQYLPDPQNCNAYY 1170
MaCht2   1178 TWSPWWSTSSSSKPTTKPSTPVVWWTEKPSTIPTTRPTTTTVAWWATKPSTTAKPTTETTPDGSTIVPPPAVVMPEVDTPSKPCEPGQHLPDATNCNAYY 1277
Clustal                 :*::: :.*                  **  ***:    :*:       ** . .  ..         *.   ...*:.*:: *   ******
```

```
RpCht1    1043 RCILGEYHKQNCAGGLHWNNENKICDWPNEAKCSSEPTTLKTTTT--RRPTTTT-----------------------------------TTTTTTTRRPT 1105
MaCht1    1278 RCILGELKKQYCAGGLHWNKRKNICDWPKEAKCREEKPYLATTT---RKPVRK------------------------------------PQTTVLWQTRT 1338
TcCht6    1135 RCVLGELRKQYCAGGLHWNKERKICDWPKSAKCEEKKPGHKPSTSSWQKPTKPSYRP--------------------------------PSTTNHWQTKT 1202
TcCht10   1040 RCVLGELRKQYCAGGLHWNKERKICDWPKSAKCEEKKPGHKPSTSSWQKPTKPSYRP--------------------------------PSTTNHWQTKT 1107
TmCht1    1171 RCVLGELRKQYCAGGLHWNKERKVCDWPKEAKCQEHKPGHKPTTPSWQKPTTTSWQKPTTTSWQRPTTTSWQKPTTTSWQRPTTRPPYRPTTTSHWQTKT 1270
MaCht2    1278 RCILGELKKQYCAGGLHWNKRKNICDWPKEAKCREEKPYLATTT---RKPVRK------------------------------------PQTTVLWQTRT 1331
Clustal        **:*** :** ********:..::****:.*** .. .   .:*   ::*.                                      . **   :  *

RpCht1    1106 TTTTTTTTRRPTTTRKPTTTTTGN----CTEGTYYPVPGKCSSFYICANGMLVEQQCAPGLNWNVESGFCDWAFSVKCKSEYNLNLRTEIRQYVG----T 1197
MaCht1    1339 TPAKVTRTTQAPQTTRPTSPETTTSKKDCVTGTYYPHE-SCSQFYVCVNGHLVEQSCAPGLSWNAQDGMCDWNFKVKCLPGNKIAQKFNLLNNQYIGDRP 1437
TcCht6    1203 TTS--TTTRPTTTVSQLIDDKCDS-------GQYYPHE-SCSSFYVCVNGHLVPQNCAPGLHYNPEEHMCDWKYKVKCVGRKQLAQKYQLPKMG----GP 1288
TcCht10   1108 TTS--TTTRPTTTVSQLIDDKCDS-------GQYYPHE-SCSSFYVCVNGHLVPQNCAPGLHYNPEEHMCDWKYKVKCVGRKQLAQKYQLPKMG----GP 1193
TmCht1    1271 TSTPRPTTTSGTTSSQLVNDKCSP-------GQYYPHE-SCSSFYVCVNGHLVPQNCAPGLHYNTQEHMCDWKYKVKCVGRKELAQMYQLPKMSF--DHP 1360
MaCht2    1332 TPAKVTRTTQAPQTTRPTSPETTT--------------------------------------------------------------------------RP 1357
Clustal        *.:  . *   .   :                                                                                   .

RpCht1    1198 RTQDSCN-KGAFAAHPNSCSKYLQCLWDKYEVHTCAPGLHWNQQQKICDWPKSAGCSKGDGETDVTIVP----VDTDVTIG------------TIKPPTS 1280
MaCht1    1438 QPYSACT-ENTFAPLAGDCTQYLHCLWGKYEVFQCAPGLHWNDQKKICDWPRGAHCSQDTDNSIIDLDT---TAKPAVTTPSRPSSKKPTTPSQWKPPTT 1533
TcCht6    1289 QPYSACS-ENAFAAYPGDCTRYLHCLWGKYEVFNCAPGLHWDNNKNICDWPEKATCDGTSNVNVVDIVT---TAKPAQSTTS--VSTT----TSWNPGST 1378
TcCht10   1194 QPYSACS-ENAFAAYPGDCTRYLHCLWGKYEVFNCAPGLHWDNNKNICDWPEKATCDGTSNVNVVDIVT---TAKPAQSTTS--VSTT----TSWNPGST 1293
TmCht1    1361 QPYSACGGENAFAAYPKDCTRYLHCLWGKYEVFNCAPGLHWSNERQICDWPEKAKCDGTSSISVAEIATMKPTEAPVHSSTE--SGSTPPSSTSRKPPTS 1458
MaCht2    1258 QPYSACT-ENTFAPLAGDCTQYLHCLWGKYEVFQCAPGLHWNDQKKICDWPRGAHCSQDTDNSIIDLDT---TAKPAVTTPSRPSSKKPTTPSQWKPPTT 1453
Clustal        :. .:*  :.:**. . .*::**:***.****. *******.::::*****. * *.   . .   : .      .  :                :* ::

RpCht1    1281 TTQWTWKPEN-----------EWSWTSTTTTEEPWPETPYKHPLSGYFKVVCYFTNWAWYRQGKGKYLPEDIDSDLCTHIIYGFAVLDFSNLIIKAHDSW 1369
MaCht1    1534 TPS-QWKPPSTTTTESSGNEWEWHPPIPPTSEKPPLSEPLK-PFSGYYKIVCYFTNWAWYRKGSGKYLPEDIDENLCTHIVYGFAVLDYSNHIIKAHDSW 1631
TcCht6    1379 TNYPEWQPT------------EWHPPIPPTSEKPPLPEELK-PQSGYFKIVCYFTNWAWYRKGSGKYLPEDIDPDLCTHIVYGFAVLDFENLIVKAHDSW 1465
TcCht10   1294 TNYPEWQPT------------EWHPPIPPTSEKPPLPEELK-PQSGYFKIVCYFTNWAWYRKGLGKYLPEDIDPDLCTHIVYGFAVLDFENLIVKAHDSW 1380
TmCht1    1459 TSYPEWQPT------------EWHPPIPPTSEKPPLPEPLK-PQSGYFKIVCYFTNWAWYRKGIGRYLPEDIDPDLCTHIVYGFAVLDFEKLIVKAHDSW 1545
MaCht2    1454 TPS-QWKPPSTTTTESSGNEWEWHPPIPPTSEKPPLSEPLK-PFSGYYKIVCYFTNWAWYRKGSGKYLPEDIDENLCTHIVYGFAVLDYSNHIIKAHDSW 1551
Clustal        *    *:*             **  . ..*:*:*      * * ***:*:***********:* *:******* :*****:*******:.: *:******

RpCht1    1370 ADFDNRYYQIRVVKFRRKGVKVLLALGGWNDSAGDKYSRLVNNPSARRKFIEHAVPFLLKYKFDGLDLDWEYPKCWQTNCNQGKDSDKEAFGQWVTEIKQ 1469
MaCht1    1632 ADFDNQFYKR-VTAYKEKGVKVSIAIGGWNDSLGDKYSKLVNNPAARRRFIEHILKFLEKYNFDGLDLDWEYPKCWQVDCKKGPDSDKEAFAAFVTELKQ 1730
TcCht6    1466 ADFDNQFYKR-VTGYKAKGIKVSLALGGWNDSQGDKYSRLVNNPAARARFIKHVLQFLEKWNFDGLDLDWEYPKCWQVDCKKGPDSDKQAFAAWVTELKQ 1564
TcCht10   1381 ADFDNQFYKR-VTGYKAKGIKVSLALGGWNDSQGDKYSRLVNNPAARARFIKHVLQFLEKWNFDGLDLDWEYPKCWQVDCKKGPDSDKQAFAAWVTELKQ 1479
TmCht1    1546 ADFDNQFYKR-VTGYKAKGVKVSLALGGWNDSQGDKYSRLVNNPAARARFIKHVLEFLEKWDFDGLDLDWEYPKCWQVDCKKGPDSDKQAFADWVTEIKA 1644
MaCht2    1552 ADFDNQFYKR-VTAYKEKGVKVSIAIGGWNDSLGDKYSKLVNNPAARRRFIEHILKFLEKYNFDGLDLDWEYPKCWQVDCKKGPDSDKEAFAAFVTELKQ 1650
Clustal        *****::*:  *. :: **:** :*:****** *****:*****:** :**:* : ** *:.***************.:*::* ****:**. :***:*

RpCht1    1470 EFRKHNLLLSAAVTPSKTIIDAGYDIKALTDNLDWISVMTYDYHGQWDKKTGHVAPMFEHPDDDYYYFNMNYSINYWIDGGVPRRKVVLGMPMYGQGFTL 1569
MaCht1    1731 AFKPKGYLLSAAVSPSKTVIDAGYNVPVLAENLDWVAVMTYDFHGQWDKQTGHVAPLFYHPEDEVAFYNSNFSLNYWISEGVPRRKIVMGMPLYGQSFRL 1830
TcCht6    1565 AFKPKGYLLSAAVSPSKTVIDAGYDVPVLAQNLDWVAVMTYDFHGQWDKQTGHVAPLYYHPEDEVVFYNANFSINYWISEGVPRRKIVMGMPLYGQSFRL 1664
TcCht10   1480 AFKPKGYLLSAAVSPSKTVIDAGYDVPVLAQNLDWVAVMTYDFHGQWDKQTGHVAPLYYHPEDEVVFYNANFSINYWISEGVPRRKIVMGMPLYGQSFRL 1579
TmCht1    1645 AFKPKGYLLSAAVSPSKTVIDAGYDVPVLAQNLDWVAVMTYDFHGQWDKQTGHVAPLYYHPEDEVAFYNANFSINYWISEGVPRRKIVMGMPLYGQSFRL 1744
MaCht2    1651 AFKPKGYLLSAAVSPSKTVIDAGYNVPVLAENLDWVAVMTYDFHGQWDKQTGHVAPLFYHPEDEVAFYNSNFSLNYWISEGVPRRKIVMGMPLYGQSFRL 1750
Clustal         *: :. ******:****:*****:: .*::****::*****:******:******:: **:*:  ::* *:*:****. ******:*:***:***.* *
```

```
RpCht1   1570 ADPKNHGLNAPAPRPAQAGEFTRAAGFLAYYEICDKIQNRGWTVVKDEEGRMGPYAYHDDQWVGFDDQETIKRKSQYIRDMDLGGGMIWALDLDDFRNTC 1669
MaCht1   1831 EKESEHGLNAKAPGPGEAGPYTRAAGFLAYYEICDNIQNKGWTVVQDPKRRMGPYAYKGNQWVSFDDREMIRIKSEYIRKMDIGGGMIWALDLDDFKNKC 1930
TcCht6   1665 EKPENHGLNAKAPGPGQAGEYTRAAGFLAYYEICHNIKTQGWTVVQDPNRRMGPYAYKGDQWVSFDDREMIKRKSEFIRKMDLGGGMIWALDLDDFKNRC 1764
TcCht10  1580 EKPENHGLNAKAPGPGQAGEYTRAAGFLAYYEICHNIKTQGWTVVQDPNRRMGPYAYKGDQWVSFDDREMIKRKSEFIRKMDLGGGMIWALDLDDFKNRC 1679
TmCht1   1745 EKESNHGLNAKAPGPGQAGEHTRAAGFLAYYEICHNIKSQGWTVVQDPKRRMGPYAYRGDQWVSFDDREMIRRKSEFIRKMDLGGGMIWARDLDDFKNRC 1844
MaCht2   1751 EKESEHGLNAKAPGPGEAGPYTRAAGFLAYYEICDNIQNKGWTVVQDPKRRMGPYAYKGNQWVSFDDREMIRIKSEYIRKMDIGGGMIWALDLDDFKNKC 1850
Clustal         . .:***** ** *.:** .*************.:*:.:*****:* : *******:.:***.***:* *: **::**.**:******* *****:* *

RpCht1   1670 GQGKHPLLSTIARTLADPRYGD-DSVTTMKPRPPKPPSTKPTPSRPTKPPSKPETTSQTMAPDKPSTTTESSMKPMPGPDGEFKVVCYFTNWAWYRQGTA 1768
MaCht1   1931 GEGRHPLLTTIRNVLADPGNGEQEPIPPIEEERPAVG--EPEPVETEEEPLPPRID-QTIAP---PPSQTTTQALVDVNSEFKVVCYFTNWAWYRQGVG 2024
TcCht6   1765 GEGRHPLLTTIRNVLADAGTGQQETIPPIDEEPPS-----VEAVEEEREEAPPSVHSSTVVP---PPQH-STTQSLVDPKSEFKVVCYFTNWAWYRQGVG 1855
TcCht10  1680 GEGRHPLLTTIRNVLADAGTGQQETIPPIDEEPPS-----VEAVEEEREEAPPSVHSSTVVP---PPQH-STTQSLVDPKSEFKVVCYFTNWAWYRQGVG 1770
TmCht1   1845 GEGRHPLLTTIRNVLADAGTGQQEPIPPIDTEEPSKPPYKPEPVEAEHTEPPPSVHSSTLEP---SPTHGTTQQALVNPNSEFKVVCYFTNWAWYRQGPG 1941
MaCht2   1851 GEGRHPLLTTIRNVLADPGNGEQEPIPPIEEERPAVG--EPEPVETEEEPLPPRID-QTIAP---PPSQTTTQALVDVNSEFKVVCYFTNWAWYRQGVG 1944
Clustal       *:*:****:** ..***.  *: :.:..:. . *         . .  .     *     .*: *    ..    :: :.: . ..****************

RpCht1   1769 KYLPSDIDPELCTHIVYGFAVLDGDKLVIKPHDSWADIDNKFYEKVTSFKEKGVKVTVAIGGWNDSAGGKYSRLVNDPGARARFTTHVVEFILKYNFDGL 1868
MaCht1   2025 KYLPSDIDPDLCTHIVYGFAVLNGDQLIIKPHDTWADFDNKFYEKVTSLKAKGIKVLIAIGGWNDSAGDKYSKLVNNPSARRRFVSHVVDFIEENNFDGL 2124
TcCht6   1856 KYLPSDIDPDLCTHIVYGFAVLNGDQLIIKPHDTWADFDNKFYEKVTAYKSKGIKVLVAIGGWNDSAGDKYSRLVNNPSARRRFIAHVVDFIETNNFDGL 1955
TcCht10  1771 KYLPSDIDPDLCTHIVYGFAVLNGDQLIIKPHDTWADFDNKFYEKVTAYKSKGIKVLVAIGGWNDSAGDKYSRLVNNPSARRRFIAHVVDFIETNNFDGL 1870
TmCht1   1942 KYLPSDIDPDLCTHIVYGFAVLNGDQLIIKPHDTWADFDNKFYEKVTAFKAKGIKVLIAIGGWNDSAGDKYSRLVNNPSSRRRFIAHVVDFIENNNFDGL 2041
MaCht2   1945 KYLPSDIDPDLCTHIVYGFAVLNGDQLIIKPHDTWADFDNKFYEKVTSLKAKGIKVLIAIGGWNDSAGDKYSKLVNNPSARRRFVSHVVDFIEENNFDGL 2044
Clustal       *********:***********:**:*:*****:***:*********: * **:** :**********.***:***:*.:* ** :***:**   *****

RpCht1   1869 DLDWEYPKCWQVDCNKGPDSDKRAFADWVSELKEAFTPHGLLLSAAVSPSKTVIDHGYDVKVLSEKLDWIAVMTYDYHGQWDKRTGHVAPMYEHEEDLDA 1968
MaCht1   2125 DLDWEYPKCWQVDCNKGPSSDKPAFAELVKELHDAFQPKGWLLSSAVSPSKRVIDAGYDVPALSKYLDWIAVMCYDYHGQWDKVTGHVAPMYAHPEDIDD 2224
TcCht6   1956 DLDWEYPKCWQVDCNKGPASDKSAFADFVKELHEAFKPKGWLLSAAVSPSRRVVDAGYDVPTLSKYLDWIAVMCYDYHGQWDKITGHVAPMYAHPEDADA 2055
TcCht10  1871 DLDWEYPKCWQVDCNKGPASDKSAFADFVKELHEAFKPKGWLLSAAVSPSRRVVDAGYDVPTLSKYLDWIAVMCYDYHGQWDKITGHVAPMYAHPEDADA 1970
TmCht1   2042 DLDWEYPKCWQVDCNKGPASDKSSFAEFVKELHEAFTPKGWLLSAAVSPSRRVVDAGYDVPTLSRYLDWIAVMCYDYHGQWDKITGHVAPMYAHPDDVDA 2141
MaCht2   2045 DLDWEYPKCWQVDCNKGPSSDKPAFAELVKELHDAFQPKGWLLSSAVSPSKRVIDAGYDVPALSKYLDWIAVMCYDYHGQWDKVTGHVAPMYAHPEDIDD 2144
Clustal       ***************** *** :**: *.**::** *:* ***:*****: *:* **** .**. ******* ********* ******** * :* *

RpCht1   1969 TFNANFSLFYWQNQGADPKKIVMGMPMYGQSFSLADNSNDGLNAPTYGGGEAGEGTRARGFLAYHEICDRVLKKGWTLVTDESGAMGPHAINKDQWVGFD 2068
MaCht1   2225 TFNTNFTIHYWIEKGADRRKLVMGMPMYGQSFSLADNSNNGLNVATYGGGEAGEETRARGFLAYYEICTNIMKKGWQVIRDRKGRLGPYAYLRDQWVSFD 2324
TcCht6   2056 TFNTNFTIHYWIEKGADRRKLVMGMPMYGQSFSLADNNQNGLNAATYGGGEAGEETRARGFLAYYEICTNVIKKGWKVVRDRRGRIGPYAYLRDQWVSFD 2155
TcCht10  1971 TFNTNFTIHYWIEKGADRRKLVMGMPMYGQSFSLADNNQNGLNAATYGGGEAGEETRARGFLAYYEICTNVIKKGWKVVRDRRGRIGPYAYLRDQWVSFD 2070
TmCht1   2142 TFNTNFTIHYWIEKGADRKKLVMGMPMYGQSFSLADNNQNGVNAPTYGGGEAGEETRARGFLSYYEICTNVINKGWTVVRDRRGRIGPYAHLRDQWVSFD 2241
MaCht2   2145 TFNTNFTIHYWIEKGADRRKLVMGMPMYGQSFSLADNSNNGLNVATYGGGEAGEETRARGFLAYYEICTNIMKKGWQVIRDRKGRLGPYAYLRDQWVSFD 2244
Clustal       ***:**::.** ::*** :*:***************.::*:*..********* ******:*:*** .:::*** :: *. * :**:* :****.**

RpCht1   2069 DAAMIKHKSEFVKKHGFGGAMIWALDLDDFRNVCGCEEYPLLRTINRVLRSYP-SGPLCSLSKELKHAITTES-------------------SESDLRSE 2148
MaCht1   2325 DIGMIRHKSEYIKAMGLGGGMIWALDLDDFKNICDCEEYPLLRTINRVLRNYAKPAPKCILGKPQ-KPTLKPT-----------TKKPTTPSTIQTQKPS 2412
TcCht6   2156 DIGMIRHKSEFIKAMGLGGGMIWALDLDDFKNLCGCEEYPLLRTINRVLRGYAKPDPKCILGRDNDKTKPKPT-----------TMKPT---TTIGYEPQ 2241
TcCht10  2071 DIGMIRHKSEFIKAMGLGGGMIWALDLDDFKNLCGCEEYPLLRTINRVLRGYAKPDPKCILGRDNDKTKPKPT-----------TMKPT---TTIGYEPQ 2156
TmCht1   2242 DIGMIRHKSEFIRAMGLGGGMIWALDLDDFRNLCGCEEYPLLRTINRVLRDYAKPDPKCILGKASSKPTQKPTPKPTQKPTPKPTQKPTPKPSTPPYEPQ 2341
MaCht2   2245 DIGMIRHKSEYIKAMGLGGGMIWALDLDDFKNICDCEEYPLLRTINRVLRNYAKPAPKCILGKPQ-KPTLKPT-----------TKKPTTPSTIQTQKPS 2332
Clustal       * .**:****:::  *:**.**********:*:*.**************.*. . * * *.:   :.  . :                  :    ...
```

```
RpCht1    2149 ----------------EITPCPQITGLLNLIEWRDKSDCTKFYTCQFGVLIQQACPLGLYFNQKALMCDWPENAGCKTDQNEIPDDNDESTPSS------ 2226
MaCht1    2413 ---------------HPVQTQPPPKSCDGRLFVSHDTNCNQYYLCNQGQLQLQSCPSGLFWNND--HCDWPENTQCHPDGSTTAPIDEVTDSNEPASEGT 2495
TcCht6    2242 ---------------EPVLP-AEVVPCQGRLFVADDTNCAQYYLCNQGQLQLQVCPNGLFWNKD--HCDWPENTECHPDASSTMAPSTTPMVPD------ 2317
TcCht10   2157 ---------------EPVLP-AEVVPCQGRLFVADDTNCAQYYLCNQGQLQLQVCPNGLFWNKD--HCDWPENTECHPDASSTMAPSTTPMVPD------ 2232
TmCht1    2342 KPSTQKPSYGTTESPEPVMP-PDSVPCRGRLFVADEKNCNQYYLCNQGELQLQVCPNGLFWNRD--HCDWPENTECHPDGTTTAAPSTTTQTLEVEVP-- 2436
MaCht2    2333 ---------------HPVQTQPPPKSCDGRLFVSHDTNCNQYYLCNQGQLQLQSCPSGLFWNND--HCDWPENTQCHPDGSTTAPIDEVTDSNEPASEGT 2415
Clustal                         : . .       . :   ...:* ::* *: * *  * ** **::*..    ******: *:.* .     .  .   .

RpCht1    2227 ----MEESTSTEMSEIESTTSANEIIDSRYKVVCYFTNWAWYRQGTGKFLPEDIDTTLCTHIIYAFAVLDSEKLEIKPHDSWADIDNEFYTKVSSLAKKG 2322
MaCht1    2496 TQETYLPPIETTSRPSYPGTEVGRGGD-DYKVVCYFTNWAWYRQGDGKYLPSDIDPELCTHINYGFAVLDSTSLTIKPHDSWADIDNEFYKKVTEYKTKG 2594
TcCht6    2318 ------KPVTTTTSR-PIGTDTSD----DYKVVCYFTNWAWYRQGDGKYLPSDIDPSLCTHINYGFAVLDSSSMTLKPHDSWADIDNEFYKKVVSYKSRG 2406
TcCht10   2233 ------KPVTTTTSR-PIGTDTSD----DYKVVCYFTNWAWYRQGDGKYLPSDIDPSLCTHINYGFAVLDSSSMTLKPHDSWADIDNEFYKKVVSYKSRG 2322
TmCht1    2437 -EVEVPEPVTTPAAP-VGGTDVTEG---AYKVVCYFTNWAWYRQGDGKYLPQDIDASLCTHINYGFAVLDGSTMTLKPHDSWADIDNEFYKKVVAFKSRG 2532
MaCht     2416 TQETYLPPIETTSRPSYPGTEVGRGGD-DYKVVCYFTNWAWYRQGDGKYLPSDIDPELCTHINYGFAVLDSTSLTIKPHDSWADIDNEFYKKVTEYKTKG 2514
Clustal                  .  *        *..         *************** **:**.***. ***** *.*****. .: :*************.**    .:*

RpCht1    2323 LKVLLGLGGWNDSLGDKYSRLVNSKEARKRFVRQAVNFIEKYKFQGLDLDWEYPKCWQVDCNKGPDSDKEGFSSLVKELSAMFKPKGWLLSAAVSPSKVV 2422
MaCht1    2595 IKVLIAIGGWNDSLGNKYSRLVNDPQARARFITNVIQFIEKWDFDGLDLDWEYPKCWQVDCNKGPDSDKQNFAALVRELSAAFKPKGLLLSSAVSPSKAV 2694
TcCht6    2407 VKVLIALGGWNDSAGNKYSRLVNDPQARAAFIAHVLAFIEEWNFDGLDLDWEYPKCWQVDCNKGPDSDKEAFAAFVRELSAAFKPKNLLLSAAVSPSKAV 2506
TcCht10   2323 VKVLIALGGWNDSAGNKYSRLVNDPQARAAFIAHVLAFIEEWNFDGLDLDWEYPKCWQVDCNKGPDSDKEAFAAFVRELSAAFKPKNLLLSAAVSPSKAV 2422
TmCht1    2533 IKVLIALGGWNDSAGSKY-RLVNDPQARARFVAHALEFIEKWGFDGLDLDWEYPKCWQVDCNKGPDSDKENFANLVRELSLAFKPKGLLLSAAVSPSKAV 2631
MaCht2    2515 IKVLIAIGGWNDSLGNKYSRLVNDPQARARFITNVIQFIEKWDFDGLDLDWEYPKCWQVDCNKGPDSDKQNFAALVRELSAAFKPKGLLLSSAVSPSKAV 2614
Clustal        :***:.:****** *.** ****. :**   *: :.: ***:: :**********************: *: :*:***  ****. ***:******.*

RpCht1    2423 VDQGYDVPVLGQYLDWISLMAYDYHGHWDGRTGHVAPLYFNQGDTFSYFNSNFSIHYWLDEGTPKEKLILGMPMYGQSFTLVNESDNGLNAPSSGPGMAG 2522
MaCht1    2695 IDAGYDVPVLSKYFDWIAVMTYDFHGHWDKQTGHVAPLYYYPGDLYDYFNANFSINYWIEKGAPPKKLVMGMPLYGQSFSLADSGKRGLNEKSYGPGEAG 2794
TcCht6    2507 IDAGYDVPALSQYLDWIAVMTYDFHGHWDKQTGHVAPLYYYPGDVYDYFNANFSLNYWIEKGADPGKIVMGMPLYGQSFSLADAADRGLNAKSYGPGEAG 2606
TcCht10   2423 MDAGYDVPALSQYLDWIAVMTYDFHGHWDKQTGHVAPLYYYPGDVYDYFNANFSLNYWIEKGADPGKIVMGMPLYGQSFSLADAADRGLNAKSYGPGEAG 2522
TmCht1    2632 IDAGYDVPTLSRYFDWIAVMTYDFHGHWDKQTGHVAPLYYYPGDAYDYFNANFSLNYWIEKGASPSKLVMGMPLYGQSFSLADSGDRGLNANPT-PGEAG 2730
MaCht2    2615 IDAGYDVPVLSKYFDWIAVMTYDFHGHWDKQTGHVAPLYYYPGDLYDYFNANFSINYWIEKGAPPKKLVMGMPLYGQSFSLADSGKRGLNEKSYGPGEAG 2714
Clustal        :* *****.*.:*:***::*:**:***** :********:  ** :.***:***::**:::*:   *:::***:*****:*.: ...***  .  ** **

RpCht1    2523 QFTRAGGFLAYYEICDTISGANWKVIRHPTGAMGPTAYKGNQWISYDDADDIRRKSKFIKDLGIGGAMIWALDSDDFRNICGCGKYPLLSTINQELRNRN 2622
MaCht1    2795 DFTRAGGFLAFYEICERVKKKGWTVTRDPEGRIGPYAYMGNQWVSYDDISEIKRKSRLVKELGLGGGMIWALDLDDFRDRCGCGRHPLLKTMNKELRGIP 2894
TcCht6    2607 EFTRAGGFLAYYEICERVKKRGWTVTRDPQGRIGPYAYLSNQWVSYDDIAEIRRKSRLVKALKLGGGMVWALDLDDFKNRCGCGKHPLLKTLNNELRGTP 2706
TcCht10   2523 EFTRAGGFLAYYEICERVKKRGWTVTRDPQGRIGPYAYLSNQWVSYDDIAEIRRKSRLVKALKLGGGMVWALDLDDFKNRCGCGKHPLLKTLNNELRGTP 2622
TmCht1    2731 EFTRAGGFLSLYEICERVKKRGWTVTRDPHGRIGPYAYMSNQWVSYDDVAEIRRKSRLIKDLHLGGGMIWALDLDDFRNRCGCGKHPLLKTMNNELRGTP 2830
MaCht2    2715 DFTRAGGFLAFYEICERVKKKGWTVTRDPEGRIGPYAYMGNQWVSYDDISEIKRKSRLVKELGLGGGMIWALDLDDFRDRCGCGRHPLLKTMNKELRGIP 2814
Clustal        :********: ****: :.  .*.* *.* * :** ** .***:****   :*:***:::* * :**.*:**** ***::  ****::***.*:*:***.

RpCht1    2623 GNTNNCT-- 2629
MaCht1    2895 SDIMFML-- 2901
TcCht6    2707 SDISVTNCT 2715
TcCht10   2623 SDISVTNCT 2631
TmCht1    2831 SDISVLNCT 2839
MaCht2    2815 SDIMFML-- 2821
Clustal        .:
```

B

```
RpCht2          1 -----MRFHQTLLFLALLCPSKERKFVLSSQLLKRDASQAEQKRVVCYYTNWSVYRPGTAKFNPQNINPYLCTHLIYAFGGLDRENGLRPYDKYQDLEQG 95
AcypiCht1       1 -----MQFLPWLLIFGSIIISD-----AEHDIQKRES--SNQKKVVCYYTNWSVYRPGTAKFSPQNINPYLCTHLIYAFGGLDKENGLRPYDKYQDIEQG 88
DpCht1          1 --------------------------------MLTKLISDSKEMRIVCYYTNWSVYRPGQAKFSPQNINPYLCTHLVYAFGGFTKENTLKPFDKYQDIEKG 69
TcCht1          1 ---------------------------------MNVSQKDSKEIRVVCYYTNWSVYRPGTAKFSPQNINPYLCTHLIYAFGGFTKENTLKPFDKYQDIEKG 68
DgCht1          1 MFWPFKKNFHFEANLILRPGAAWQTLFLLCALAYCINVASSEGRVVCYYTNWSVYRPGTAKFNPQNINPYLCTHLVYAFGGFTKDNQMKPFDKYQDIEQG 100
DaCht1          1 ------------------------MLFLLCALAYCINEASSEGRVVCYYTNWSVYRPGTAKFNPQNINPYLCTHLVYAFGGFTKDNQMKPFDKYQDIEQG 76
Clustal                                                  :.: ::************* ***.************:*****: ::* ::*:*****:*:*

RpCht2         95 RGYAKFTGLKTYNKALKTMLAIGGWNEGSARFSPLVADEDRREFVKNVVKFLRVNHFDGLDLDWEYPAFRDGGKTHDKENYAQLVQVYHRCGGAVITML 195
AcypiCht      189 -GYAKFNGLKTYNKNLKTLLAIGGWNEGSARFSKLVDDEDKRKEFVKNVIKFLRQNNFDGLDLDWEYPAYRDGSKPSDKENYASLVK------------- 171
DpCht1         70 -GYAKFTGLKTYNKDLKTLIAIGGWNEGSTRFSSMVASSERRKELVKNTIKFLRQNHFDGLDLDWEYPTFRDGGKPRDRDNYAQLVK------------- 155
TcCht1         69 -GYAKFTGLKTYNKNLKTMLAIGGWNEGSSRFSPMVANPERRKELIKNAIKFLRQNHFDGLDLDWEYPSFRDGGKSRDKDNYAQLVQ------------- 154
DgCht1        101 -GYAKFTGLKTYNKQLKTMIAIGGWNEASSRFSPLVASSDRRQHFIKNILKFLRQNHFDGIDLDWEYPAHREGGKSRDRDNYAQFVQ------------- 186
DaCht1         77 -GYAKFTGLKTYNKQLKTMIAIGGWNEASSRFSPLVASPDRRQQFIKNILKFLRQNHFDGIDLDWEYPAHREGGKSRDRDNYAQFVQ------------- 162
Clustal             *****.******* ***::*******.*:*** :* . ::*:.::** :**** *:***:*******:.*:*.*. *::***.:*:

                                                         ↓
RpCht2        196 IYLLFRRELREEFERESSKTGRVRLLLTMAVPAGIEYIDKGYDIPKLNKYLDFMNILSYDYHSAFEPSVNHHSPLYSLEEESEYNYDSQLTIDATVKHYL 295
AcypiCht      172 -------ELREEFNRESSKTGRSRLLLTMAVPAGIEYIDKGYDIPELNKHLDFMNLLTYDYHSSFEPAVNHHSPLYPLEEDSEYNFDAKLNIDHTIKHYL 264
DpCht1        156 -------ELREEFERESEKTGRPRLLLTMAVPAGIEYINKGYDVPKLTKYLDWMNILSYDYHSAFEPAVNHHAPLYSLEEDSEYNYDGELNIDYTIKHYL 248
TcCht1        155 -------ELREEFDRESEKTGRPRLLLTMAVPAGIEYINKGYDVPKLTKYLDWMNILSYDYHSAFEPAVNHHAPLYPLEEPSEYNYDTELNIDYTIQHYL 247
DgCht1        187 -------ELRAEFEREAEKTGRTRLLLTMAVPAGIEYIDKGYDVPKLNKYLDWFNVLTYDFHSSHEPSVNHHAPLYSLEDDSEYNYDAELNIDYSIKYYL 279
DaCht1        163 -------ELRAEFEREAEKTGRTRLLLTMAVPAGIEYIDKGYDVPKLNKYLDWFNVLTYDFHSSHEPSVNHHAPLYSLEEDSEYNYDAELNIDYSIKYYL 255
Clustal                   *** **:**:.**** **************:****:*:*.*:**::*:*:**:**:.**:****:***.**: ****:* :*.** ::::**

RpCht2        296 MKGADANKLVLGIPTYGRSYTLFNEASTDLGAPADGPGDQGDATREKGYLAYYEICSKVK--DGWTVVHPKNTAMGPYAYKGNQWVGYDDENIKKLSRRY 393
AcypiCht      265 ASGADKEKLVLGIPTYGRSYTLFNRESKDIGAPSDGPGEKGEATREKGYLAYYEICGNLKKDDSWTIEQPKPKAMGPYAYKDNQWVGYDDEEFVKLKAKY 364
DpCht1        249 KMGADPNKLVLGIPTYGRSYTLFNPEAYEIGSPADGPGDMGEATRENGYLAYYEICENVKNQ-DWEVEEPFPKAMGPIAFKDNQWVGYDDENIVRKKAEY 347
TcCht1        248 KKGADPSKLVLGIPTYGRSYTLFNPDANEIGAPADGPGDMGEATRENGYLAYYEVCEYIKTQ-NWEVVQPNPDAMGPYAFKDNQWVGYDDDKIARKKAEY 346
DgCht1        280 KAGADRDKLVLGIPTYGRSYTLINEESTELGAPSEGPGEQGDATREKGYLAYYEICQTLKDDPEWTVVQPNANIMGPYAYRRNQWVGYDDEAIVRKKAEY 379
DaCht1        256 KAGADRDKLVLGIPTYGRSYTLINEESTELGAPSEGPGEQGDATREKGYLAYYEICQTLKDDPEWTVVQPNANVMGPYAFRRNQWVGYDDEAIVRKKAEY 355
Clusta  l            *** .**************:*  : ::*:*::***: *:****:*******:*   :*      * : .*     *** *:: ********: : : . .*

RpCht2        394 VNEKHLGGIMIWSIDNDDFRGNCHQRAYPLIEAAKEAMLGKAATKQVPSAVRSSK-----------------------------TSSSKSSKLKNQRSS 463
AcypiCht      365 VNENDLGGIMFWSIDNDDFRGSCHSRPYPLIEAGKEALLG--DSKVAPSQGKETK-----------------------------TTLSKSRPKPRPVAR 432
DpCht1        348 VAENSLGGIMFWAIDNDDFRGNCHGKPYPLIEAGKEALINAYGLTEDNLISPPAKPTKLPKKQRPRTSRTTTTERSEE-IVEETTTRRRRIKPKIRNDNQ 446
TcCht1        347 VAEKGLGGIMFWSIDNDDFRGNCHGKPYPIIEAAKEALISAYGLTDENLVSPPTKPIKTKTRNRTQSSSKSTDENSEKKKSTSSIVSRRRNRIKTKSEEL 446
DgCht1        380 VVAHGLGGIMFWAIDNDDFRGTCSGKPYPLIEAAKEAMLDAYGLG-INEVAKPSAPQKP-SRSRSRDNEGNSNRNRVSSKPE---ASPRRPSAGRRPSSS 475
DaCht1        356 VVAQGLGGIMFWAIDNDDFRGTCNGKPYPLIEAAKEAMVEALGLG-INEVAKPSGPQKP-SRSRSRDN--GSTRNRLNGKPENTSTSVRPANGARRPAVS 451
Clustal           *  : *****:*:********.*  :.**:***.***::                  :                                   .          .

RpCht2        464 TTTTSPPVSS--------------------TVTTPEPPTTPDPGSDFVCKDEGFFPHPRDCKKYFWCLDSGPSNLGIVAHQFTCPSGTKLVDVSDSCDFA 543
AcypiCht      433 TTTTSAPQEKSD-----------------VSTTTPEPPTTPDTGTDFTCKDEGFFPHPRECKKYFWCLDSGPSNLGIVAHQFTCPSGLVFNKISDSCDYT 515
DpCht1        447 LATPAKKRRNESGRKAAETSTYSSLKIVTPAYTTPEPPATPDMGGAFKCEDEGFFPHPKDCKKYFWCLG-GPGDTGIVAHQFTCPAGLHFNKAADSCDYS 545
TcCht1        447 SNSQSKSHRKES-RRTTEGPVYSSLELVTPSYTTPAPPSTPDLGGGFKCEDEGFYPHPKDCKKYYWCLS-GPGELGIVAHLFTCPAGLYFNKAADSCDYT 544
DgCht1        476 STTTAG-PSTTIRLTEVEG---QSLYIGGRGSTTPPPPTTPDPGSDFKCEEEGFFQHPRDCKKYYWCLDSGPSGLGIVAHQFTCPSGLYFNPAADSCDFA 571
DaCht1        452 RTTQSPPPSTTFKLTDAEG---SSLYIGGRASTTPPPPTTPDPGSDFKCEEEGFFQHPRDCKKYYWCLDSGPSGLGIVAHMFTCPSGLYFNPAADSCDFA 548
Clustal              : :    .                           *** **:*** *   * *::***: **::****:***. **.   ***** ****:*  :   :****::

RpCht2        544 RNVICKKKSTTTTSTTSTTTPRTTTRATT------------------TLRSSLSRVASTTTPFPPPLQDYED----------------DSEEDPQTIKQL 609
AcypiCht      516 RNVVCKDKKTSDTTTTTTTTTTTPKPIKS------------------GIKTTTTTTTSTTQTPEVEDDSEDEF--------------EEEEDPKEIKQL 583
DpCht1        546 QNVFCNKKSKPSTTTTKAGASTSTEASSSAKPTVNSVFSTTRAPSKITAATSKTTINFRTTTTTEAYEDEEYEDEEEE-EPNKR---EESEEDPQVIKEL 641
```

```
TcCht1       545 RNVLCNKKLSKATTTTTT--TTTTEASTLKT-------STARVPPKITAATSRTTV-FRTSTTTEAYDD-EYEYEDDV-EENK-----NSEEDPKVIKEL 628
DgCht1       572 RNVPCKTKKSTTTPPVSSTSTTTTTARPTSSYNRVTAAPVARPVYPRFTSTTSTTTTTARTTTPAEEEVLEYEEDGND-SPNLGKLGSDAEEDPQVIKEL 670
DaCht1       549 RNVPCKTKKSTTVAPLTSTTTTTTTTARS---NRVTAAPTARPVYPRTSSTSTSTTTTT--TTPEPVEDLEYEEDTDEQSPNK---SHDGEEDPQVIKEL 640
Clustal          :** *: * .  ... .:  . :.                          :: :         .   :  *                 : ****: **:*

RpCht2       610 IHLIKKLGGVDELEKQLEGTTG-----------SDVTTP-SISKSLFERILHR----------NGPSGPQNEGLTRETVRAP----YRRDKPQYVTIRRE 683
AcypiCht     584 ITLIKKLGGVAELEKQLNIKDGS----------TEVTTP-AISKSLYNKVLKLPPNRFQSSYINGP-GPQSEGLDKKDDSID----YKKDKPQYVTIRRQ 667
DpCht1       642 IDLIRKAGGIEELEKQLKFGKDFSPSTS----SSDATTPSAISKSLYERVLSKTSKN-----------------------------------TLSALKKT 702
TcCht1       629 LDLIKKAGGIEELEKQLKIHEDGSASVS----NSDTTTPSSISKSLVERVLGKGAKVGGKKNSRGP--QNEGLNTHEDKETT----QDKGRPKYTTITRQ 718
DgCht1       671 IDLIRKVGGVEQLEKHLLRNKDGTITLKENSASGVQTTPSTISKSLYDRVLSRPGTLGTFTRNRFNTFKIQSSGTAESGSTTDESSSQINSSKYSSVLRG 770
DaCht1       641 IDLIRKVGGVEQLEKHLLRNKDGSITLKENSANGAVTTPSTISKSLYDRVLSRPGTLSSLTRNRLK--IAEASGTEATTSTS--VSAGNTYSKYSSVLRG 736
Clustal          : **:* **: :***:*    .              *** :***** :::*                                            :: :

RpCht2       684 RPS------------------TTTEGLN-------LAEYADVDDVVEPDDRDLEEIPKYKGLHYS-SRESDAAE----NSDPPAKSERHFRPKYQSA--- 750
AcypiCht     668 RPSKDVVDNIKESEEEDIPTDATTERFKPTYKTVDRTRFRDLGEVAD-ENSESDSLPKYTTISRTKSSESNPEE----TTSPKYVTLRRQRPTNKED--- 759
DpCht1       703 NRNSGGR---QGNGEEASRGKQSDTRGRPQYKTLTRQRLVRSFF--IPRKILNFIFCVNKVFN------------------------RRT---------- 763
TcCht1       719 RSTNNKN---EDEEEESSDEASKSTKKQPEYVNIRRARVSTTTE--EPEENSKSQLNRNKILGEEDETEDEQPSRKKTSG-PQYVNIRRQRPSTTEETST 812
DgCht1       771 NSRQGP----QNEGLEQLAEFDGFLKERKQYVTINRNRGTATRDDKATDNNSDDDIDEREEPSQPKQQSAETTRRPFSSATPSYTSLRRSRPTTTSP--- 863
DaCht1       737 NSRQGP----QNEGIDKLAEFDGFLKERKQYVTINRQR--------SPNQGDAEEHSQHEEEDEENEQVETTTRRALSSVTPSYTSLRRTRPTTMAP--- 821
Clustal          .                          .         .           .                                     *:

RpCht2       751 --------------------EQFR-PKHPSTEDQEASTKKYSSLVRSRPSTFR----------------------------------------------- 782
AcypiCht     760 --------------------EISDEEITPVAVSSTSQAPRYVSLLRQRSTTTTTTPDDRLIETTTKDNVEEPRENKNTKVSQMTLTTESSIISTILFNTE 839
DpCht1       764 ---------------------------------------------------------------------------------------------------- 763
TcCht1       813 SKYTVIRRGTTTEPAEPEEDETTKSNVVSTSTEKIDLKSRYSILRRGSTTEATTTESGSISSTSSPKRRGTTLPSVPERKRTRGTLAPGTTPSSEDTTTT 912
DgCht1       864 ------------VPIES-VTELEEEQVAPVAIKEQTQTKHYATLSR--TRGRTTERPESTASDTAGVLVSSTTNR------------------------- 923
DaCht1       822 ------------LVGESSKQEEEEEEVVEKNSRSQQQVKSYATLNR--ARERTTTGPGTTTTEATEAVPSSTTTRYKYFERTRPTKAA-TLEASKDSEEP 906
Clustal

RpCht2       783 ----------------------------------DHTSSTSNTPRILTSTR----------------VVTQIVEGSTQRQ-------------------R 813
AcypiCht     840 PFTETIATTVYVPTFTTAKNSETSKILTLITEKMSDTSSTAKAPVSIDSTINNSISTTKAPTSTVTNVVTSIYEAATERQ-------------------R 920
DpCht1       764 ---------------------------------------------------------------------------------------------------- 763
TcCht1       913 RYKSIKRGSTSEAPTTDKTLPEDSTLKYSTFTRTPASTQAAPAATEPETAVTVNVQLLTTPESLTVKTTSLSSTKQSEQSNIESIGERVYQTQTPLLLEP 1012
DgCht1       924 ---------------------------------------------------------------------------------------------------- 923
DaCht1       907 ADQEDEEEEYEEENITVQRKQSTNTRKYASIGRRSTTTTAT-----PETTTTSGTETAKANNNNIHYNSNNNKNSQAKQNNIIPT--------------T 987
Clustal

RpCht2       814 VPLHRLHELLPD--------------------------------------------------------------------TDNLIKSSTKSELSTQTSKK 881
AcypiCht     921 VRVKNIQNFLLEHKKTEPVTQSVTHTTTPLTTTLSTTMENIAPVEEPTQKSILKGRFGGPVHFRPTLRKLIGIPENNGTTTEKFIEATTEKIVESTTEKK 1020
DpCht1       764 ---------------------------------------------------------------------------------------------------- 763
TcCht1      1013 RPFSKTSTRSPTPVVTESSTRITKVSDSNYNLRLKPKLSQTQTEIISTTTLTTEKPTPFRTRAPSRFKLTTASYDQQATKQANRARKRFSTTTELYSDNY 1112
DgCht1       924 ---------------------------------------------------------------------------------------------------- 923
DaCht1       988 VTEESSTTALPTPTSTNQEPETTTETNTNETTEPNDRSSTTTTTNNPSTTFTSTPTPSLATTTP--------------------------TTTIPGVDSE 1061
Clustal
```

```
RpCht2     882 ----RISNRLIRKRP---------------------------SQLDTFAISASATT--EFPLNK------RNQQIRPPLKERSESQNVT----------- 931
AcypiCht  1021 NRLNKYTNRFIRPASNFSTENISTTSKIFVTSTTESTSESSTRQINRFRQTTSSATDKQLPLTRKFVARYRQRTTELPVINNTEVQEVQRSRFFRSRRPI 1120
DpCht1     764 ---------------------------------------------------------------------------------------------------- 763
TcCht1    1113 EEFDLSRTYRPSEIADLSSLTAVDFVALKELTRNSNSLRQRRPRPESTTPRTSSFKSRRLISNK-------NEQNVEDQVNSSSDKTHRIRGFTRSPPVV 1205
DgCht1     924 ---------------------------------------------------------------------------------------------------- 923
DaCht1    1062 SSADGSPNPLIPESPDLAPPTTTTEPELATTTTTT-------PKTTTPVTTTSTGIHSELNNDI-------DNDNDSDDSEVTKPKTQYKYATTNRRRIT 1147
Clustal

RpCht2     932 ----------------------------------------------------------------------------TLTKKRTQSLVSTAESSNTFIRNR 955
AcypiCht  1121 SSTTTTTEETITELLNVEENADNVRYETTPYTPTTMHVPTTNVEEISTNDEEIKKTTIDSFEPIKYSTTEITTIIQPITNKITKSFRGSVRANDGFQDSN 1220
DpCht1     764 ---------------------------------------------------------------------------------------------------- 763
TcCht1    1206 STTTEGTTRHSLRNRKVVRRLRPTSSSKLLSQNSDNKENVVPFKKRLVRPNTETENLIENSSLNSLFNRRLTRPTEAPNEEQSDDVSAEEGENIKLSESN 1305
DgCht1     924 ---------------------------------------------------------------------------------------------------- 923
DaCht1    1148 NTTATRTTNTTANNNNSEAAN-DASPRNGLSSFNKIRASINTNPGRRPPQTQPEHTTIEPNSSASLSSVSSPRPFGYPRRRTRPSSSIPTTTTNQSDNNN 1246
Clustal

RpCht2     956 FMKTRNPTDDDRE-------------------DGKDP---------------------------LLSREFSRE---SSIIRKPLFRYRSGTTSS------ 1000
AcypiCht  1221 IDKSRSPSLGRQNSRFLKEEQKILFIRVMPSPDGRSQNEFTSNPVKNITRNRGRVRAYDSLELNTLTDELTNEDRPNELFRGSETKFRPTTTSTTTES-- 1318
DpCht1     764 ---------------------------------------------------------------------------------------------------- 763
TcCht1    1306 IRENSNVNNNNNNILSPDDGQKVRRKVLKRIKPKVDDEKIVTEPNIVIRTRKIVRKLTPTESTITSTTETTVPGRKRKIIRRLRPTTELKENSTQKTLYI 1405
DgCht1     924 ---------------------------------------------------------------------------------------------------- 923
DaCht1    1247 STDNDNTNETD-----------AVASVVKKTRPSPGDRPKVSAS-LQTATRSTLHNLHHQD---QQESQVEVAGNGGNSLRHEVVSSSLSQTP------- 1324
Clustal

RpCht2    1001 ----------------------QISKEDSTLSHTTPVSTMNTKNYSEFPRDPNSFILS-EESEKDRYTDEVSEDQVTDAE---------IYGQLEEQVSG 1069
AcypiCht  1319 -----TEEIQKDRIRQRITRPVQRSTTTTTTTEESPPSSSATESYSSKVNRRRKFRPEYTTTSTSSYEEEITTQKTRNPERRFRSRTPLLKNTITTSTTP 1413
DpCht1     764 ---------------------------------------------------------------------------------------------------- 763
TcCht1    1406 RGRPFLAHFNNEESGVDASTIKPDSSKYSTPNRGTTEESEVTEQFIANSENTDSYKGTTEEAEAKKQLISNSENSSQRTTDSYKGTTEEADSSTERTIDR 1505
DgCht1     924 ---------------------------------------------------------------------------------------------------- 923
DaCht1    1325 ----------NHIDDADNGGITQTKTKY-TWRAVRKPTARPRNSLARDDKDSRRFAGKQLNDISLDQDLSSTTKFRSRRLNSAEDESEVAHAAAESDQKA 1413
Clustal

RpCht2    1070 DSKEQSSR-----------------------------LPDLANVAVAAIQSLATSAPS--------------------------------TQSTEKYKST 1108
AcypiCht  1414 ETLEDSDREFFSTSSQPYFDDSSLFAKTRDDRKIGGSTEDLANTAVVAIHTLATAPPSDYE-------------FRPSTHSPQQSVFRETTQNREYFEPQ 1500
DpCht1     764 ---------------------------------------------------------------------------------------------------- 763
TcCht1    1506 SKYSTLNRGTIDSTREESFDKEIITTDRGPSRFRSTTEGTIDRTKYSTLNKGTTEEPEVREHFISNSDTSTERTIDRSKYSTLNRGTTESTKEEKIDEET 1605
DgCht1     924 ---------------------------------------------------------------------------------------------------- 923
DaCht1    1414 RSYQSINHQSGKKI-------------------------------------------------------------------------------------- 1427
Clustal

RpCht2    1109 PHAAVG------------------------------------KSVLSGTVRPSSRPSRPFRPTTRAGVTRVTSSPRPFSKP---TAQP------------ 1158
AcypiCht  1501 APHSIDSFDVVQPARLPEQSLRQSAQPQLPPPPPPSIKLPAVSFQPAPFQPSPFQAGPFQPVPYPGLPFQANNAFAPVARQSPTTALPPATSRQTFILR- 1599
DpCht1     764 ---------------------------------------------------------------------------------------------------- 763
TcCht1    1606 ITTNRGPSRFRNSNTSTEGTIDRSKYSTLNRGTTEEAEVKEQHISSSDASTEGTIDRSKYSTLNRGTTEEAEVKEQHISNSDASTEGTIDRSKYSTLNRG 1705
DgCht1     924 ---------------------------------------------------------------------------------------------------- 923
```

```
DaCht1    1428 ---------------------------------------------------------------------------------------------------- 1427
Clustal

RpCht2    1159 -----------------TTPARGATSPHRTSLIYEDYDADY--YVEP--------VDLPLSGKVRIHADGYIECLDIGNFPHPFSCRKFISCAKMENGRL 1231
AcypiCht  1600 ------------RGPSRTTPLGTAATSAPPQYLNDNTQEQYADYSEPEQASDSSEQRVALKGKVRIHGDGYIECLDMGSFPHPFSCQKFISCAKTEYGAL 1687
DpCht1     764 ---------------------------------------------------------------------------------------------------- 763
TcCht1    1706 TTESAKEEKIDEEIISTNRGPSRFRIYRPTVFHDDDEEEEEDDSLKTRIVEPLFTEEITESNKLSNNATNKTDDLNKEEADEENSTIETNEENSTEEHKD 1805
DgCht1     924 ---------------------------------------------------------------------------------------------------- 923
DaCht1    1428 ---------------------------------------------------------------------------------------------------- 1427
Clustal

RpCht2    1232 LGWEYTCPKGLSFDPIGGICNWNAGLGCKE---------------------------------------------------------------------- 1301
AcypiCht  1688 FGWEYTCPKGLSFDQVGGICNWSSGPVCNN---------------------------------------------------------------------- 1717
DpCht1     764 ---------------------------------------------------------------------------------------------------- 763
TcCht1    1806 EEDNEDIPVTTVKPYINPILNRQKNRPAFQRPKLTTQKPLSSSTTSRNRYKTFGRSTTAPSKETNEEITQEKFVPKNLDRNRKVSTSTTTTEVAPEIEQT 1905
DgCht1     924 ---------------------------------------------------------------------------------------------------- 923
DaCht1    1428 ---------------------------------------------------------------------------------------------------- 1427
Clustal

RpCht2    1302 ---------------------------------------------------------------------------------------------------- 1301
AcypiCht  1718 ---------------------------------------------------------------------------------------------------- 1717
DpCht1     764 ---------------------------------------------------------------------------------------------------- 763
TcCht1    1906 TDSVQINDTESDKTTLESIEVTTLKETEEVVNATTESRTILVETTTLRTTETTPSHLPRSTSRTRPKFNVPKRLTTPESRFSPKLRTQSTTPKAPTSLLK 2005
DgCht1     924 ---------------------------------------------------------------------------------------------------- 923
DaCht1    1428 ---------------------------------------------------------------------------------------------------- 1427
Clustal


RpCht2    1302 ---------------------------------------------------------------------------------------------------- 1301
AcypiCht  1718 ---------------------------------------------------------------------------------------------------- 1717
DpCht1     764 ---------------------------------------------------------------------------------------------------- 763
TcCht1    1906 DKFTRKYTTTERNDFVEEEDIPEEDEVENQSSTQRTTRPKARPSNRPDFRKSTEAPKPTEDLKGIDTDAVKNRNKNLFSKKRKMNTPFAGHPLNQSILTT 2105
DgCht1     924 ---------------------------------------------------------------------------------------------------- 923
DaCht1    1428 ---------------------------------------------------------------------------------------------------- 1427
Clustal

RpCht2    1302 ---------------------------------------------------------------------------------------------------- 1301
AcypiCht  1718 ---------------------------------------------------------------------------------------------------- 1717
DpCht1     764 ---------------------------------------------------------------------------------------------------- 763
TcCht1    2106 TPTISESVTKPSTEPFETTEYLTTLHHIFAETERVTENSLPTTTSSKIEKLIEVNRIVEVHEMNNTEERDNKVVDKVGVINRVTVVKVVDGNFNPEDNEI 2205
DgCht1     924 ---------------------------------------------------------------------------------------------------- 923
DaCht1    1428 ---------------------------------------------------------------------------------------------------- 1427
Clustal

RpCht2    1302 ---------------------------------------------------------------------------------------------------- 1301
AcypiCht  1718 ---------------------------------------------------------------------------------------------------- 1717
```

```
DpCht1     764 ---------------------------------------------------------------------------------------------------- 763
TcCht1    2206 TKSPKLDDFEIATVREIPNREDRRYEQVAESAEIIDGRSNINIITPRPYYSTEASTISLEGLFQTDTPQKLSDKFNLNQLKQGTNDEELLETGNSRYVNV 2305
DgCht1     924 ---------------------------------------------------------------------------------------------------- 923
DaCht1    1428 ---------------------------------------------------------------------------------------------------- 1427
Clustal

RpCht2    1302 ----------------------------------------------------------------- 1301
AcypiCht1 1718 ----------------------------------------------------------------- 1717
DpCht1     764 ----------------------------------------------------------------- 763
TcCht1    2306 RVLKEDDYITMKAEVVEVTPKISKDIKIVPIQVEMSRKLIAPSDFVTKVEQGVPKVTLQILKPQN 2370
DgCht1     924 ----------------------------------------------------------------- 923
DaCht1    1428 ----------------------------------------------------------------- 1427
Clustal
```

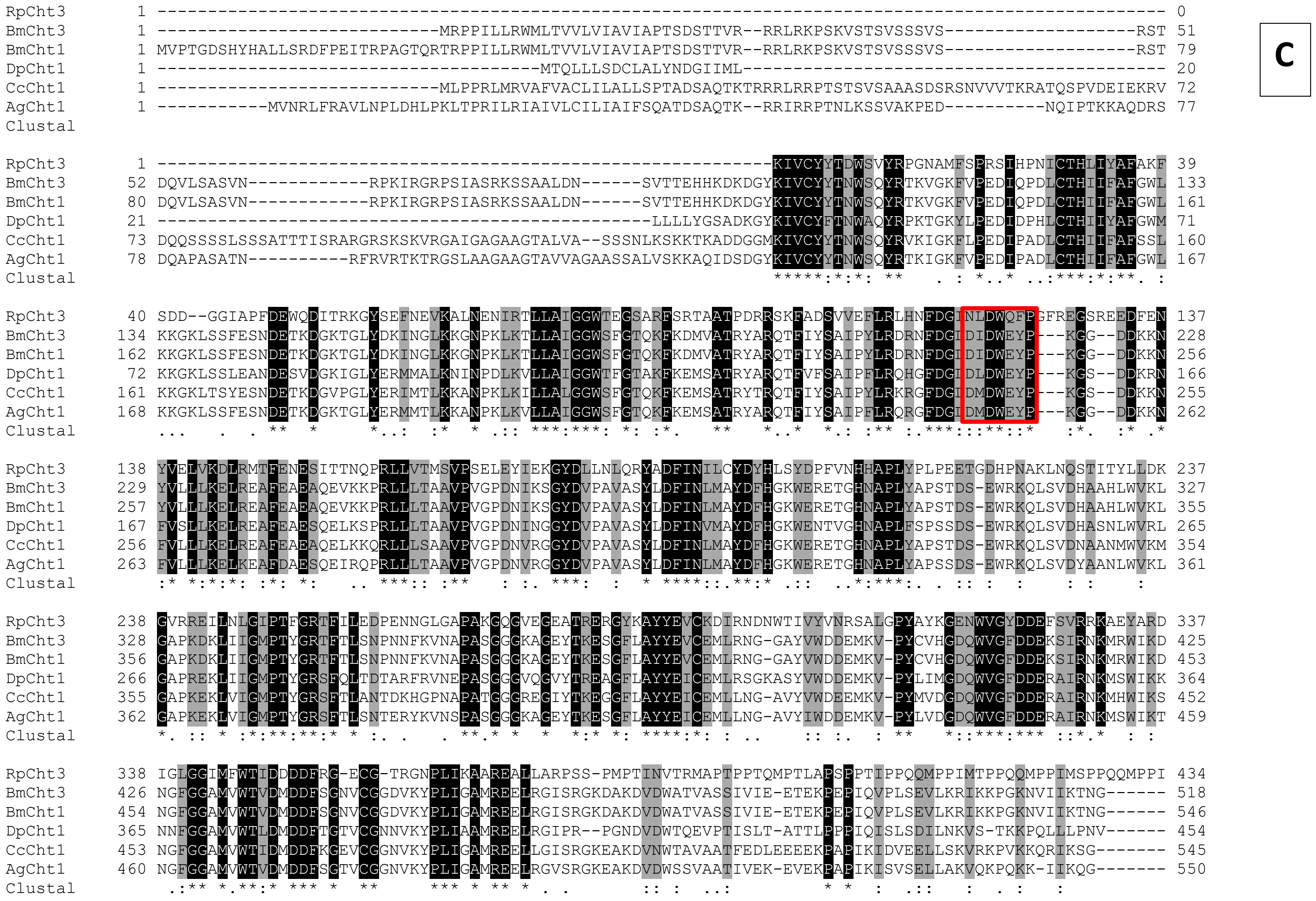
C
RpCht3 1 -- 0
BmCht3 1 ----------------------------MRPPILLRWMLTVVLVIAVIAPTSDSTTVR--RRLRKPSKVSTSVSSSVS-------------------RST 51
BmCht1 1 MVPTGDSHYHALLSRDFPEITRPAGTQRTRPPILLRWMLTVVLVIAVIAPTSDSTTVR--RRLRKPSKVSTSVSSSVS-------------------RST 79
DpCht1 1 -------------------------------------MTQLLLSDCLALYNDGIIML--- 20
CcCht1 1 ----------------------------MLPPRLMRVAFVACLILALLSPTADSAQTKTRRRLRRPTSTSVSAAASDSRSNVVVTKRATQSPVDEIEKRV 72
AgCht1 1 -----------MVNRLFRAVLNPLDHLPKLTPRILRIAIVLCILIAIFSQATDSAQTK--RRIRRPTNLKSSVAKPED----------NQIPTKKAQDRS 77
Clustal
RpCht3 1 ---KIVCYYTDWSVYRPGNAMFSPRSIHPNICTHLIYAFAKF 39
BmCht3 52 DQVLSASVN------------RPKIRGRPSIASRKSSAALDN------SVTTEHHKDKDGYKIVCYYTNWSQYRTKVGKFVPEDIQPDLCTHIIFAFGWL 133
BmCht1 80 DQVLSASVN------------RPKIRGRPSIASRKSSAALDN------SVTTEHHKDKDGYKIVCYYTNWSQYRTKVGKFVPEDIQPDLCTHIIFAFGWL 161
DpCht1 21 --LLLLYGSADKGYKIVCYFTNWAQYRPKTGKYLPEDIDPHLCTHIIYAFGWM 71
CcCht1 73 DQQSSSSLSSSATTTISRARGRSKSKVRGAIGAGAAGTALVA--SSSNLKSKKTKADDGGMKIVCYYTNWSQYRVKIGKFLPEDIPADLCTHIIFAFSSL 160
AgCht1 78 DQAPASATN----------RFRVRTKTRGSLAAGAAGTAVVAGAASSALVSKKAQIDSDGYKIVCYYTNWSQYRTKIGKFVPEDIPADLCTHIIFAFGWL 167
Clustal *****:*:*: ** . : *..* ..:***:*:**. :
RpCht3 40 SDD--GGIAPFDEWQDITRKGYSEFNEVKALNENIRTLLAIGGWTEGSARFSRTAATPDRRSKFADSVVEFLRLHNFDGINLDWQFPGFREGSREEDFEN 137
BmCht3 134 KKGKLSSFESNDETKDGKTGLYDKINGLKKGNPKLKTLLAIGGWSFGTQKFKDMVATRYARQTFIYSAIPYLRDRNFDGIDIDWEYP---KG--DDKKN 228
BmCht1 162 KKGKLSSFESNDETKDGKTGLYDKINGLKKGNPKLKTLLAIGGWSFGTQKFKDMVATRYARQTFIYSAIPYLRDRNFDGIDIDWEYP---KG--DDKKN 256
DpCht1 72 KKGKLSSLEANDESVDGKIGLYERMMALKNINPDLKVLLAIGGWTFGTAKFKEMSATRYARQTFVFSAIPFLRQHGFDGIDLDWEYP---KGS--DDKRN 166
CcCht1 161 KKGKLTSYESNDETKDGVPGLYERIMTLKKANPKLKILLALGGWSFGTQKFKEMSATRYTRQTFIYSAIPYLRKRGFDGIDMDWEYP---KGS--DDKKN 255
AgCht1 168 KKGKLSSFESNDETKDGKTGLYERMMTLKKANPKLKVLLAIGGWSFGTQKFKEMSATRYARQTFIYSAIPFLRQRGFDGIDMDWEYP---KGG--DDKKN 262
Clustal ... . . ** * *..: :* * .:: ***:***: *: :*. ** *..* *.: :** :.***::**::* :*. :* .*
RpCht3 138 YVELVKDLRMTFENESITTNQPRLLVTMSVPSELEYIEKGYDLLNLQRYADFINILCYDYHLSYDPFVNHHAPLYPLPEETGDHPNAKLNQSTITYLLDK 237
BmCht3 229 YVLLLKELREAFEAEAQEVKKPRLLLTAAVPVGPDNIKSGYDVPAVASYLDFINLMAYDFHGKWERETGHNAPLYAPSTDS-EWRKQLSVDHAAHLWVKL 327
BmCht1 257 YVLLLKELREAFEAEAQEVKKPRLLLTAAVPVGPDNIKSGYDVPAVASYLDFINLMAYDFHGKWERETGHNAPLYAPSTDS-EWRKQLSVDHAAHLWVKL 355
DpCht1 167 FVSLLKELREAFEAESQELKSPRLLLTAAVPVGPDNINGGYDVPAVASYLDFINVMAYDFHGKWENTVGHNAPLFSPSSDS-EWRKQLSVDHASNLWVRL 265
CcCht1 256 FVLLLKELREAFEAEAQELKKQRLLLSAAVPVGPDNVRGGYDVPAVASYLDFINLMAYDFHGKWERETGHNAPLYAPSTDS-EWRKQLSVDNAANMWVKM 354
AgCht1 263 FVLLLKELKEAFDAESQEIRQPRLLLTAAVPVGPDNVRGGYDVPAVASYLDFINLMAYDFHGKWERETGHNAPLYAPSSDS-EWRKQLSVDYAANLWVKL 361
Clustal :* *:*:*: :*: *: .. ***:: :** : :. ***: : * ****::.**:* .:: ..*:***:. . :: : : : : :
RpCht3 238 GVRREILNLGIPTFGRTFILEDPENNGLGAPAKGQGVEGEATRERGYKAYYEVCKDIRNDNWTIVYVNRSALGPYAYKGENWVGYDDEFSVRRKAEYARD 337
BmCht3 328 GAPKDKLIIGMPTYGRTFTLSNPNNFKVNAPASGGGKAGEYTKESGFLAYYEVCEMLRNG-GAYVWDDEMKV-PYCVHGDQWVGFDDEKSIRNKMRWIKD 425
BmCht1 356 GAPKDKLIIGMPTYGRTFTLSNPNNFKVNAPASGGGKAGEYTKESGFLAYYEVCEMLRNG-GAYVWDDEMKV-PYCVHGDQWVGFDDEKSIRNKMRWIKD 453
DpCht1 266 GAPREKLIIGMPTYGRSFQLTDTARFRVNEPASGGGVQGVYTREAGFLAYYEICEMLRSGKASYVWDDEMKV-PYLIMGDQWVGFDDERAIRNKMSWIKK 364
CcCht1 355 GAPKEKLVIGMPTYGRSFTLANTDKHGPNAPATGGGREGIYTKEGGFLAYYEICEMLLNG-AVYVWDEEMKV-PYMVDGDQWVGFDDERAIRNKMHWIKS 452
AgCht1 362 GAPKEKLVIGMPTYGRSFTLSNTERYKVNSPASGGGKAGEYTKEGSFLAYYEICEMLLNG-AVYIWDDEMKV-PYLVDGDQWVGFDDERAIRNKMSWIKT 459
Clustal *. :: * :*:**:**:* * :. . . **.* * * *:* *: ****:*: : .. :: :. : ** *::***:*** ::*.* : :
RpCht3 338 IGLGGIMFWTIDDDDFRG-ECG-TRGNPLIKAAREALLARPSS-PMPTINVTRMAPTPPTQMPTLAPSPPTIPPQQMPPIMTPPQQMPPIMSPPQQMPPI 434
BmCht3 426 NGFGGAMVWTVDMDDFSGNVCGGDVKYPLIGAMREELRGISRGKDAKDVDWATVASSIVIE-ETEKPEPIQVPLSEVLKRIKKPGKNVIIKTNG------ 518
BmCht1 454 NGFGGAMVWTVDMDDFSGNVCGGDVKYPLIGAMREELRGISRGKDAKDVDWATVASSIVIE-ETEKPEPIQVPLSEVLKRIKKPGKNVIIKTNG------ 546
DpCht1 365 NNFGGAMVWTLDMDDFTGTVCGNNVKYPLIAAMREELRGIPR--PGNDVDWTQEVPTISLT-ATTLPPPIQISLSDILNKVS-TKKPQLLLPNV------ 454
CcCht1 453 NGFGGAMVWTIDMDDFKGEVCGGNVKYPLIGAMREELLGISRGKEAKDVNWTAVAATFEDLEEEEKPAPIKIDVEELLSKVRKPVKKQRIKSG------- 545
AgCht1 460 NGFGGAMVWTVDMDDFSGTVCGGNVKYPLIGAMREELRGVSRGKEAKDVDWSSVAATIVEK-EVEKPAPIKISVSELLAKVQKPQKK-IIKQG------- 550
Clustal .:** *.**:* *** * ** *** * ** * . . :: : ..: * * : .:: : . : :

```
RpCht3     435 IMKLVTYNLLFSVFVLFTTAQNEQEKSEKRIVCYYTNWSPYRP-VAKFTPASIDPNVCTHLIYAFATFSNNFGLVPTDEYQDITNNFNSLKEKNVNLKTL 533
BmCht3     519 ------------------AALSDKNKREPQVLCYLTSWSAKRPSAGRFTPENVDPKLCTHIIYAFATLKDHKLAEADDKDADMYDKVVALREKNPNLKIL 600
BmCht1     547 ------------------AALSDKNKREPQVLCYLTSWSAKRPSAGRFTPENVDPKLCTHIIYAFATLKDHKLAEADDKDADMYDKVVALREKNPNLKIL 628
DpCht1     455 ------------------NNAAPVNGSNAKTICYYTSWSANRPGLGRFSPEEVNTELCTHLVYAFGALKDYRLSLVGSDELDVHARIQTLREKNPDLKII 536
CcCht1     546 ------------------LVAKEQNTRPAQVFCYLTSWSAKRPGAGKFLPSNIDPKLCTHVVYAFATLKDHKLSEATDDDPENYEQVIALREENPDLQIL 627
AgCht1     551 ------------------LATVEKNSRPAQVFCYLTSWSVKRPGAGKFEPKDVDASLCTHIVYAFATLKDHKLTEANENDPDMYDEVIALREKNPDLQIL 632
Clustal                              :    : .** *.**  **  .:* * .::..:***::***.::.:        ..  :   .. :*:*:* :*: :

RpCht3     534 ISLGGTSEGSARFSKMVESEEKINYFIKSAIEFLRKYNFDGINLDWQFPTIGYGSKKEDMANYVKLTQALKEGFENEAKLTNKTSLLLTICLPSEIEYIE 633
BmCht3     601 LAIGGWAFGSTPFKELTSNVFRMNQFVYEAIEFLRDYQFNGIDVDWEYPRG-----ADDRAAFVSLLKELRLAFEGEAKTSGQPRLLLTAAVPASFEAIA 695
BmCht1     629 LAIGGWAFGSTPFKELTSNVFRMNQFVYEAIEFLRDYQFNGIDVDWEYPRG-----ADDRAAFVSLLKELRLAFEGEAKTSGQPRLLLTAAVPASFEAIA 723
DpCht1     537 LAIGGWAVGSAPFKELTSNIFRMNQFVYDSTEFLRANNFDGIDIDWEYPRG-----ADDRASFLNLIKELRLAFEGESVTNKMPRLLLTAAVPASPEAIA 631
CcCht1     628 LAIGGWAFGSKPFKELTSNVFRMNQFVYEAIDFLRDYKFNGIDVDWEYPRG-----AEDRAAYVNLLRELRVAFEGEAKSSGQPRLLLTAAVPASFEAIA 722
AgCht1     633 LAIGGWAFGSTPFKELTSNVFRMNQFVYEAIEFLREYQFNGIDVDWEYPRG-----ADDRKAYVDLLRELRVAFEGEAKTSGQPRLLLTAAVPASFEAIA 727
Clustal        :::** : **  *.::...  ::* *: .: :***  :*:*:::**::*         :*   ::.* : *: .**.*:  .  . **** .:*:. * *

RpCht3     634 QGYDL-NLQNYVDFFNILTYDYHRSNEGNVSHHAPLYPLQQEISANINTNLNADSTIQHLLKKGLKSDKLNLGIPTYGRNYKLDNVDKHDIGAPSKGNQG 732
BmCht3     696 AGYDVPEISKYLDFINVMTYDFHGQWERQVGHNSPLFPLESATSY--QKKLTVDYSAREWVRQGAPKEKLMIGMPTYGRSFTLINETQYDIGAPASEGGE 793
BmCht1     724 AGYDVPEISKYLDFINVMTYDFHGQWERQVGHNSPLFPLESATSY--QKKLTVDYSAREWVRQGAPKEKLMIGMPTYGRSFTLINETQYDIGAPASEGGE 821
DpCht1     632 AGYDVPEVVKYVDFLNVMTYDFHGHWEGQVGHNSPLFPLETAASH--QRKLTVDYGAREWVKLGAPAEKIMIGMPTFGRTFTLADLSKFDIGSPASGGGI 729
CcCht1     723 AGYDVPEISKYLDFINVMTYDFHGQWERTVGHNSPLFPLEATTGY--QKKLTVDFSAREWVKQGAPKEKLLIGMPTYGRSFELVNETQFDIGAPASGGGK 820
AgCht1     728 AGYDVPEISKYLDFINVMTYDFHGQWERQVGHNSPLFPLDSASNY--QKKLTVDYSAREWVKQGAPKEKLLIGMPTYGRSFTLVNQTQFDIGAPASGGGL 825
Clustal         ***: :: :*:**:*::***:*   *  *.*::**:**:   .   : :*..*   :. :: *   :*: :*:**:**.: * :  :.***:*:. .

RpCht3     733 SKEFTITNRIIKTEFQICKTTKEQNWTVVHVNESSIMPYTFKGEDWIGFDDEESVQAKGEYARKHNLGGIMFWTLDYDDFNGECGNGKFPLIN------- 825
BmCht3     794 AGRFTNEAGFMS-YYEICEFLREDNTTLVWDNEQ-MVPFAYRGDQWVGFDDERSLKTKMAWLKEEGFGGIMVWSVDMDDFRGSCGTGKFPLITTMKQELS 891
BmCht1     822 AGRFTNEAGFMS-YYEICEFLREDNTTLVWDNEQ-MVPFAYRGDQWVGFDDERSLKTKMAWLKEEGFGGIMVWSVDMDDFRGSCGTGKFPLITTMKQELS 919
DpCht1     730 AGPYTSEAGFLA-YYEVCDFLQEDNTTLVWDNEQ-QVPFAYRDDQWVGFDDERSLRTKVSWLKEQGFGGVMIYSLDMDDFRGHCGSGRYPLLKAVRQELT 827
CcCht1     821 AGKFTNEAGFLS-YYEVCTFLAAENTTLVWDSEQ-QVPFAYRGNQWVGFDDERSLKTKMEWLKEQGFGGIMVWSIDMDDFSGRCGAGKYPLLNSLNDELK 918
AgCht1     826 PGKFTNEAGFLS-YYEICAFLGVDNTTLVWDSEQ-QVPFAYRKDQWVGFDDERSLKTKMEWLKEEGFGGIMVWSIDMDDFSGRCGSGKYPLLNVLNSELK 923
Clustal        .  :*    ::   :::*     :* *:*  .*.  :*:::: ::*:*****.*:::*  : ::..:**:*.:::* *** * ** *::**:.

RpCht3     826 ---------------------------------------------------------------------------------------------------- 825
BmCht3     892 --DYKVKLEYDGPYETVLT---SGQYTTKDPTEVTCEEEDGHISYHKDQADCTMYYMCEGERKHHMPCPSNLVFNPNENVCDWPENVEGCAHHTQAPPAR 986
BmCht1     920 --DYKVKLEYDGPYETVLT---SGQYTTKDPTEVTCEEEDGHISYHKDQADCTMYYMCEGERKHHMPCPSNLVFNPNENVCDWPENVEGCAHHTQAPPAR 1014
DpCht1     828 ADNYQVQFVYDGPYERSASSLITGKPAPKDPNVVTCDEEEGHISYHPDKANCAMYYMCEGERKHHMPCPVQLVFNPSQNVCDWPENVPGCETHFTAGTGR 927
CcCht1     919 --DYKVELEYEGPYESHGP---RGAYTTKDPHDVTCEEEDGHISYHKDWNDCTHYYMCEGERKHHMPCPANLVFNPQENVCDWPENVEGCHEPTEAPA-- 1011
AgCht1     924 --DYKVALEYDGPYESFGP---RGAYTTKDPNEVTCAEEDGHISYHPDKADCTHYFMCEGERKHHMPCPANLVFNPNENVCDWPENVEGCQHHTQAPAA- 1017
Clustal

RpCht3     826 - 825
BmCht3     987 R 987
BmCht1    1015 R 1015
DpCht1     928 R 928
CcCht1    1012 - 1011
AgCht1    1018 - 1017
Clustal
```

D

```
RpCht4     1 ------MLPSKLWCLLFVFVLAANYITGSNKKTVLCYISSWAVYRRSQGQFTISDVDVNLCTHLVYAFLGINETTNTVVSRDSWADYVTNGRGFGQLKDL 94
TcCht3     1 MVSLKNFCAVFVIFVVAFMDEVKALTGPSHGKVVVCYLGTWSVYRPGRGSFTIEHLDPALCTHIVYSFAGLDEKTDSIKSLDPFQD-LADDYGKEGYQKL 99
PhCht1     1 -------------------MKKKTRLKPIHDKHVVCYIGTWSVYRPGKGSFKIENIDGNLCTHIVYAFVGLNATTNTLRSIDPYYD-LEENYGKGSFKKM 80
DpCht3     1 ---------------------MAFLASPSHGKVVVCYIGTWAVYRPARGSFTVENLEPSLCTHVIYAFAGLNPQEDSIKSLDPWQD-LTEHYGKGGYQRI 78
DpCht4     1 --MLSIWSGLCVIVIVCGCCIEAQNTGPSHGKVVVCYIGTWAAYRPARGSFTVENLEPSLCTHVIYAFAGLNPQEDSIQSLDPWQD-LTEHYGKGGYQRI 97
MrCht2     1 -------------------MSISSLIRSTRDKVVTCYVASWAIYRPQNGKFGIDDLNPNLCTHLVYAFAGLNDTTWTIRSLDPYMD-IEN--GIGNYKKM 78
Clustal                                   . * * **:.:*: **  .*.* :..::  ****::*:* *::    :: * *.: * : :  *    : :

RpCht4    95 VKLKDEAPNLKVLISVGGWSEGSEKFSNMAASTVKRIAFVESVFQFLRRNQLDGVDICWMFPTDRGGIAEDKRNFATLLKELRTRLNEDNRILTVLMTGK 194
TcCht3   100 TRLKHRYPHLKVTLAIGGWNEGSLKYSELAKHANRRSRFVSSVVDFLRKYDFDGLDLDWEFPGKRGGAPEDKLNFLILVKELKAAFSKYNLLLTAAFGAG 199
PhCht1    81 TQLKLKYPNLKVTLAVGGWNEGSANYSNMALVPENRRKFINSVMEYVTKYNFDGFDLDWEFPAARGGRPEDKQNFALLVKELKQELGKKNLILTAALGAA 180
DpCht3    79 TTLKLRYPHLKVSLAIGGWNEGSKNYSQLVAEPPRRSKFVASAVDFIRKYNFDGLDLDWEFPGKRGGAPEDKLNFLLLVKELKSAFRKHNLLLTAAFGAG 178
DpCht4    98 TTLKLRYPHLKVSLAIGGWNEGSKNYSQLVAEPPRRSKFVASAVDFIRKYNFDGLDLDWEFPGKRGGAPEDKLNFLLLVKELKSAFRKHNLLLTAAFGAG 197
MrCht2    79 TQLRHKYPDLNVLLAIGGWNEGSKNYSELASSPERRSRFIDSVVYYLRKYDFNGFDLDWEFPGSRGGVPADKENFVSLVKELKEAFQKSNYLLTAAITAN 178
Clustal      . *: . *.*:* :::***.*** ::*::.  . .*  *: *.. :: : :::*.*: * **  *** . ** **  *:***:  : : * :**. : .

RpCht4   195 RYIVDPGYDVRTISQSVDYINVMSYDFNGPWERKTGVNAPLISENEFNV-------------------EFSIDYLIKKGATPSKILVGIPTFGHSFTMKN 275
TcCht3   200 KDTIDAAYDVKGLSLYLDFIHMMCYDYHGAWDQKTGANAPLQSSDVLNV-------------------EFSINYMLKLGAPAKKLVLGVPLYGRTFLLPE 280
PhCht1   181 INTINTAYDVPEISKHLDLLHFMCYDYHGPWDKTVGANAPLTSKDSLDL-------------------ESSITHLLQLGAPPHKLVVGIPAYGHTFLTKN 261
DpCht3   179 KDTIDVAYDVEGLGVYLDFIHMMCYDYHGAWDQKTGANAPLRSSDVLNVVCTGFPQSAEFPRNGVSLQEYTINYMIKLGAPMHKLVLGVPLYGRTYVLPE 278
DpCht4   198 KDTIDVAYDVEGLGVYLDFIHMMCYDYHGAWDQKTGANAPLRSSDVLNVVCTGFPQSAEFPRNGVSPQEYTINYMIKLGAPMHKLVLGVPLYGRTYVLPE 297
MrCht2   178 KGTIDTAYNIPELSLYLDHIHVMAYDYHGTWDNKVLPNSPLDS------------------------VKDTMNYLLNKGAPANKLVLGLPMYGRTFILAS 254
Clustal         :: .*::  :.  :* ::.*.**::*.*:...  *:** *                         : :: :::: **.  *:::*:* :*:::   .

RpCht4   276 VPD-----NGNYLKVPSDNGYLSQWTREKGIIGYNEVCYELST-TSGWKEMWDDASSTPYAVNHKKFVSYDNQRSILEKVKLVNLKGLGGAMVYTLDTDD 370
TcCht3   281 PYDPESGRKPKLGAVARNIGFQGPFTRENGFMGYNEICLEVKNKSQNWAKFWDSESKTPYAVSGNKVLTYDDVQSIEEKVKFAIEKDLGGIMVWSIDTDD 380
PhCht1   262 VTN------PKMGTPITGPGPEGVFTKQQGFQGYNEICTELLKDDEKWSKHWDDESSTPYAVNGNHVIAFDDEKSIGEKVNLGLKYKVGGFMIWSIDTDD 355
DpCht3   279 ALHASSKRKPKLGIVSTSVGFQGPITREPGFMGYNEICLELKNKTEPWMKFWDSESATPFAIRNTQVITYDDDKSIYEKVKFAMEKKLAGIMVWSVDTDD 378
DpCht4   298 ALHASSKRKPKLGIVSTSVGFQGPITREPGFMGYNEICLELKNKTEPWMKFWDSESATPFAIRNSQVITYDDDKSIYEKVKFAMEKKLAGIMVWSVDTDD 397
MrCht2   255 KLN-SSDESP-IGQRSLSEGFNGSYTGQNGFMGYNEICEELVT-SNNWRIGWDDSSNTPYAIKDDYVIVYDNPRSLKAKVEYAKSLNLAGVMVWSIDTDD 351
Clustal        .              . *  .  * : *: ****:* *: .  . *   **. * **:*:    .: :*: :*:  **:      :.* *::::****

RpCht4   371 FRGLCQG------DR-------YPLVTAVRDHLNK-----------------------DRPITISNVSIVSHNYG----------------- 409
TcCht3   381 FHGDCAD----VGEDGHFEN--FPLMRAINKAIPK---------SIEDRRNSVQ---WTG-VNTSGAGSSSSLLFLVAMAIALFIKF----- 448
PhCht1   356 FHGDCDKERLKVKES-AQDKYTFPLLRAVHTAIQQYKLQDDDNNDIPEVGTNDIDDPKENNVVPSFQSSITANFVVIFTSGAFLLILNYIVK 446
DpCht3   379 FQGDCAN----ADDD-SPQN--FPLMRAIDKSIET---------SLEEIKNSIIKGKFTPRRNSSCRLSTSSNMLVFITALFVLGRYSL--- 451
DpCht4   398 FQGDCAN----ADDD-SPQN--FPLMRAIDKSIET---------SLEEIKNSIIKGKFTPRRNSSCRLSTSSNMLVFITALFVLGRYSL--- 470
MrCht2   352 FKGKCAS----LKDSLEQENPTYPLMRSINVALYS---------DSNKSKLPTP----SPTPSTAERIYSSANFRENSGQGEGI-------- 418
Clustal      *:* *        :        :**: ::   :                               :     :
```

E

```
RpCht5     1 --------MLTVTAGRG-------------GKVVVCYVGTWAAYRPGRGAFSIDEIEPDLCTHIVYSFAGLNTTTWTITSLDPYMDLEDDYGKGMYKKMT 79
BmCht2     1 --MAVVLCLLLIFALMAPSLDGQILGGPMHGKAVVCYVASWAAYRPNDGQFTLDNLQPALCTHLVYSFAGLNETTFTIKSLDPWQDLEKDYGKAGYKRIV 98
TcCht3     1 MVSLKNFCAVFVIFVVAFMDEVKALTGPSHGKVVVCYLGTWSVYRPGRGSFTIEHLDPALCTHIVYSFAGLDEKTDSIKSLDPFQDLADDYGKEGYQKLT 100
PhCht1     1 --------------------MKKKTRLKPIHDKHVVCYIGTWSVYRPGKGSFKIENIDGNLCTHIVYAFVGLNATTNTLRSIDPYYDLEENYGKGSFKKMT 81
DpCht3     1 ----------------------MAFLASPSHGKVVVCYIGTWAVYRPARGSFTVENLEPSLCTHVIYAFAGLNPQEDSIKSLDPWQDLTEHYGKGGYQRIT 79
DpCht4     1 --MLSIWSGLCVIVIVCGCCIEAQNTGPSHGKVVVCYIGTWAAYRPARGSFTVENLEPSLCTHVIYAFAGLNPQEDSIQSLDPWQDLTEHYGKGGYQRIT 98
Clustal                                     .* ****:.:*:.***  * *.::.::  ****::*:*.**:    ::  *:**: ** ..***  ::::.

RpCht5    80 SLKKSHPGLKITLAIGGWNEGSANYSILAADPAKRETFTRSVMNYVRKFQFDGVDLDWEFPTQRGGQPEDKYNFVALIKDLKSSLTAEGYILTAALSASL 179
BmCht2    99 ALKERYPHLKVTIAIGGWNEGSERYSKMASSPETRKAFINSVMIFLNQYKFDGIDLDWEYPSKRGGKPDDKANYVSLVKELKEAFEPKSLILTAALGAGK 198
TcCht3   101 RLKHRYPHLKVTLAIGGWNEGSLKYSELAKHANRRSRFVSSVVDFLRKYDFDGIDLDWEFPGKRGGAPEDKLNFLILVKELKAAFSKYNLLLTAAFGAGK 200
PhCht1    82 QLKLKYPNLKVTLAVGGWNEGSANYSNMALVPENRRKFINSVMEYVTKYNFDGFDLDWEFPAARGGRPEDKQNFALLVKELKQELGKKNLILTAALGAAI 181
DpCht3    80 TLKLRYPHLKVSLAIGGWNEGSKNYSQLVAEPPRRSKFVASAVDFIRKYNFDGIDLDWEFPGKRGGAPEDKLNFLLLVKELKSAFRKHNLLLTAAFGAGK 179
DpCht4    99 TLKLRYPHLKVSLAIGGWNEGSKNYSQLVAEPPRRSKFVASAVDFIRKYNFDGIDLDWEFPGKRGGAPEDKLNFLLLVKELKSAFRKHNLLLTAAFGAGK 198
Clustal        **  :* **:::*:******* .** :.  .  *  *  *.: :: ::.***.*****:*  *** *:** *:  *:*:**  :   . :****:.*.

RpCht5   180 DTLKAGYDLLQVYRNLDLIHLMCYDYHGLWETQTGANAPLGPPTDPLTANRCTLNQ-------------------EATVKYVLNHGVKPDKLVLGVPMYG 260
BmCht2   199 ETMESAYDLSKLSRYLDLIHMMCYDYHGTWDGVVGPNAPLRG-----LDNEDVLSV-------------------EYTVKYMISQGVSPYKLVLGLPMYG 274
TcCht3   201 DTIDAAYDVKGLSLYLDFIHMMCYDYHGAWDQKTGANAPLQS--------SDVLNV-------------------EFSINYMLKLGAPAKKLVLGVPLYG 273
PhCht1   182 NTINTAYDVPEISKHLDLLHFMCYDYHGPWDKTVGANAPLTS--------KDSLDL-------------------ESSITHLLQLGAPPHKLVVGIPAYG 254
DpCht3   180 DTIDVAYDVEGLGVYLDFIHMMCYDYHGAWDQKTGANAPLRS--------SDVLNVVCTGFPQSAEFPRNGVSLQEYTINYMIKLGAPMHKLVLGVPLYG 271
DpCht4   199 DTIDVAYDVEGLGVYLDFIHMMCYDYHGAWDQKTGANAPLRS--------SDVLNVVCTGFPQSAEFPRNGVSPQEYTINYMIKLGAPMHKLVLGVPLYG 290
Clustal      :*:. .**:  :   **::*:******* *:  .*.****                *.                  * ::.:::. *.   ***:*:* **

RpCht5   261 RTFLLANAEE----SSGRLGEPSVKSAGFQGPFTRTDGFLGFNEICNELRTGN--WTEHWDQASSTPFANHKDRIISYDNVESIKSKVGF---------- 344
BmCht2   275 RTFVLNDAST----KRLQFGVTPVESAGFKGPYTREAGFMGYNEICAEVSNKSSPWEYHWHEGSATAYLRDGARVISYDDPRAIAAKVKFAVDNNLGGLM 370
TcCht3   274 RTFLLPEPYDPESGRKPKLG-AVARNIGFQGPFTRENGFMGYNEICLEVKNKSQNWAKFWDSESKTPYAVSGNKVLTYDDVQSIEEKVKFAIEKDLGGIM 372
PhCht1   255 HTFLTKNVTN------PKMG-TPITGPGPEGVFTKQQGFQGYNEICTELLKDDEKWSKHWDDESSTPYAVNGNHVIAFDDEKSIGEKVNLGLKYKVGGFM 347
DpCht3   272 RTYVLPEALHASSKRKPKLG-IVSTSVGFQGPITREPGFMGYNEICLELKNKTEPWMKFWDSESATPFAIRNTQVITYDDDKSIYEKVKFAMEKKLAGIM 370
DpCht4   291 RTYVLPEALHASSKRKPKLG-IVSTSVGFQGPITREPGFMGYNEICLELKNKTEPWMKFWDSESATPFAIRNSQVITYDDDKSIYEKVKFAMEKKLAGIM 389
Clustal      :*::  :           ::*     . * :*  *:  ** *:**** *: .     *  .*.. * *.:    :::::*: .:*  ** :

RpCht5   345 ---------------------------------------------------------------------------------------------------- 344
BmCht2   371 VWSIDTDDFRGHCGIDDRTFKDFTDRYNRMVNDPLLKEALKTLKLGDEARNLHRGTAYSLTDGRLELRLPQPQLSNYQLMQTVNAATTLALEEKRILDEM 470
TcCht3   373 VWSIDTDDFHGDCADVGEDG-----------------------------------------------HFENFPLMRAINKAIPKSIEDRR--NSV 418
PhCht1   348 IWSIDTDDFHGDCDKERLKVKES---------------------------------------------AQDKYTFPLLRAVHTAIQQYKLQDDDNNDI 400
DpCht3   371 VWSVDTDDFQGDCANADDD-----------------------------------------------------SPQNFPLMRAIDKSIETSLEEIK--NSI 415
DpCht4   390 VWSVDTDDFQGDCANADDD-----------------------------------------------------SPQNFPLMRAIDKSIETSLEEIK--NSI 434
Clustal

RpCht5   345 -------------------------------------------- 344
BmCht2   471 SRV-------NRDNEIVQTLSGCSRPERSLALVS---LLIYKLFF- 505
TcCht3   419 Q----------WTG-VNTSGAGSSSSLLFLVAMA---IALFIKF-- 448
PhCht1   401 PEVGTNDIDDPKENNVVPSFQSSITANFVVIFTSGAFLLLILNYIVK 446
DpCht3   416 IKG-------KFTPRRNSSCRLSTSSNMLVFITA---LFVLGRYSL 451
DpCht4   435 IKG-------KFTPRRNSSCRLSTSSNMLVFITA---LFVLGRYSL 470
Clustal
```

F

```
RpCht6      1 MSPRLVDLVMWARNMEARLPKGS-FLDGARVVCYFSNWAIYRPGLGRYTIDDIPADMCTHVIYSFVGVSNVTWELLVLDPEVDVEQNGFKNFTLLKQKYS 99
PcCht1      1 MGSWNFWLCGSAVVLLFVATVSSQPTDDGRVVCYFSNWAIYRPGLGRYTIDDIPGEYCTHIIYSFIGVSNVTWEVLVLDPEVDVEQNGYKNFTSLKEKYP 100
TcCht2      1 -----MKLLLAVATIITLLQPYDAT--PARIVCYFSNWAIYRPGIGRYAQEDLPVDLCTHIVYSFIGVNDADWSVLVIDPELDVDQNGFRNFTNLKKTHP 93
TcCht5      1 -----MKLLLAVATIITLLQPYDAT--PARIVCYFSNWAIYRPGIGRYAQEDLPVDLCTHIVYSFIGVNDADWSVLVIDPELDVDQNGFRNFTNLKKTHP 93
NvCht1      1 MNPQAGKLLLSLGALVALFACTATGEQPGRVVCYFSNWAVYRPGIGRYAIDDVPADLCTHLIYSFIGVSNVTWEVLVLDPELDVDQNGYRDFTALKKKFP 100
DpCht2      1 -----MNWIVAILSATLLLQCTEA----AKVVCYWGTWAVYRPGLGSYGMKDVPVDLCTHVIYSFIGVDDKSWEILIIDPEFDIEQTGFRNFTDLKKTHP 91
Clustal                                    .::***:..**:****:* *  .*:* : ***::***:**.:  *.:*::***.*::*.*:::** **:...

RpCht6    100 HLKTSLAVGGWGEGGRKYSALVSARDRRDTFIRSVVDTMHKYEFDGFDLDWEYPGAADRGGSFSDKDRFLYFVEELRRAFDAEGKNWEITMAVPLAKFRL 199
PcCht1    101 GIKTSVAVGGWGEGGRKYSGLVSMKERRDSFIKSVVEFMYKYDFDGFDLDWEYPGAADRGGSFSDKDLFFYFVEELRRAFDKEGKGWEITMAVPLAKFRL 200
TcCht2     94 KVKLQVAVGGWAEGGSKYSAMVAEKSRRDAFIRSVISFMNQYEFDGFDLDWEYPGAADRGGSFSDKDKFFYFVEELRRAFDKEGKNWEITMAVPIAKFRL 190
TcCht5     94 KVKLQVAVGGWAEGGSKYSAMVAEKSRRDAFIRSVISFMNQYEFDGFDLDWEYPGAADRGGSFSDKDKFFYFVGELRRAFDKEGKNWEITMAVPIAKFRL 190
NvCht1    101 NLKTEVAVGGWGEGGRKYSALVAVKQRRDTFIKSVVEFMKKYDFDGFDLDWEYPGAADRGGKFSDKDMFFYFVEELRRAFNRAGRGWEITMAVPLAKFRL 200
DpCht2     92 QVKFQVAVGGWAEGGEKYSAMVADKSRRDTFIRSVVDFMNTYGFDGFDLDWEYPGAADRGGKFSDKNVFLYFVQELRRAFDKEGKGWEITMAVPVAKFRL 191
Clustal         :* .:*****.*** ***.:*: :.***:**:**:. *  * *****************.****: *:*** ******:  *:.********:*****

RpCht6    200 NEGYHVPELCENVDAVHVMAYDLRGNWAGFADVHSPLYKRPHDQYAYEKLNVNDGLQLWVDFGCSPDKLVVGVPFYGRSFTLSQGNNNYGLGTYINKEAG 299
PcCht1    201 NEGYHVPELCELVDAIHVMAYDLRGNWAGFADVHSPLYRRPHDQYAYETLNVNDGLQLWVDYGCSPTKLVVGVPFYGRSFTLSAGNTNYNLGSYINKEAG 300
TcCht2    191 QEGYHVPELCELLDAIHVMSYDLRGNWAGFADSHSPLYKRPHDQWAYEKLNVNDGLQLWVDYGCSPKKLIVGIPFYGRTFTLSNSNTNYNPGTYINKEAG 290
TcCht5    191 QEGYHVPELCELLDAIHVMSYDLRGNWAGFADSHSPLYKRPHDQWAYEKLNVNDGLQLWVDYGCSPKKLIVGIPFYGRTFTLSNSNTNYNPGTYINKEAG 290
NvCht1    201 QEGYHVPELCENLDAIHVMSYDLRGNWAGFADVHSPLYKRPHDQWAYEKLNVNDGLQLWEDMGCPANKLVVGVPFYGRTFTLSASNNNYKPGTYINKEAG 300
DpCht2    192 DEGYHVPELCETLDAIHVMTYDLRGNWAGFADTHSALYKRPHDQWAYEKLNVNDGLLLWVNYGCPPKKLIVGVPFYGRSFTLSQGNNNYNLGTYINKEAG 291
Clustal        :********* :**:***:*********** **.**:*****:***.******* ** : **.. **:**:*****:**** .*.**  *:*******

RpCht6    300 GGAPAKYTNAKGFWAYYE------EKDSQWVQKWDDAGYCPYAYKDTQWVGYENIKSLQIKMDWIKSKGYAGAMTWAIDMDDFQGLCGEKNPLIKVLWNN 393
PcCht1    301 GGAPGNYTKAKGFISYYEICSEIQEEDSGWTIKWDEAGYCPYTYKGTQWIGYENEKSLQIKMDWIKSKGYAGAMNWAIDMDDFNGLCGTKNPLIKVLYKN 400
TcCht2    291 GGSPGPYTNATGFLAYYEICPEILNKDSGWTKKWDEHGKVPYAYKGNQWVGYEDPESVQIKMDWIKSKGYGGAMTWAIDMDDFHGLCGPKNPLINILYAN 390
TcCht5    291 GGSPGPYTNATGFLAYYEICPEILNKDSGWTKKWDEHGKVPYAYKGNQWVGYEDPESVQIKMDWIKSKGYGGAMTWAIDMDDFHGLCGPKNPLINILYAN 390
NvCht1    301 GGAPGKYTGAKGFLAYYEICPEVNDPESGWTKKWDEHGRVPYAYKGTQWVGYEDPESVQIKMDWIKSKGYAGAM WAIDMDDFHGICGRKNDLTLILHEN 400
DpCht2    292 GGSPGPYTNATGMLAYYEICEEVDNKEGSWIKQWDEAGMVPYAYKGTQWVGYEDPESIQIKMDFIKSKGYGGAMTWSVDMDDFQGICGPKNVLLHILHAN 391
Clustal        **:*. ** *.*: :***      : :. *  :**: *  **:**..**:***: :*:*****:******.***.*::*****:*:** ** *  :* *

RpCht6    394 MNNYDVPMPTVTTTPRPEWARPPSTVS--------------APGVKPTKKP---TTTREPLPPAPSTTAS-------------STTTTKTTLHSTTT--- 460
PcCht1    401 MKDYKVPAPTVTTSPRPEWSRPKSTTSTSDDEYQKPTTVRTTPAYKPRPKPKPKPTTVRPETEKPSVEKP-------------VQKPTRKPVKPTTQKPE 487
TcCht2    391 MNSYSVPEPTISTTPRPEWARPPSTPS--------------------ADGSKPVQTTPKPGQWVPEK--------------------STSTTQRTT---- 446
TcCht5    391 MNSYSVPEPTISTTPRPEWARPPSTPS--------------------ADGSKPVQTTPKPGQWVPEK--------------------STSTTQRTT---- 446
NvCht1    401 MKNYIVPKPEYNTTPRPEWSRPKSTTA--------------------NPAEGSYTTPSRETEETTPR--------------------PTRPNRPTAP--- 457
DpCht2    392 MKGYTVPDPTITTSPRPEWARPPSTPS--------------------ADG---AATTQQPGQWSSSKPPTRSTTKAPEAASRPESTSSKAPTRRTTKAP- 467
Clustal        *:.* ** *  .*:*****:** ** :                           *. .     .                     .  . : *:

RpCht6    461 --------QEAP-----KTSEIPDTITEVVEEITEGNKPDENNEEESEVPDNEQVDGLVDCTNDGFRPHKQCDKYYQCVHQMPVVFKCKPKTVWNQDKLI 547
PcCht1    488 ESGGCGCDEESPSKPSATTTIKPIRTTTPKRKTTTTEEVDLESVSESNTGDNE-VPAAVDCAEDGFKPHPACNKYYQCVYGEPLEFTCKLGTVWHQEMTI 586
TcCht2    447 --------------------------------TVSTTEAPPAPAVSNPQNEVDGSVA-PKECKQD-FMPHELCDKYYRCVHGKPTEFACRPGTVFHTQSNI 513
TcCht5    447 --------------------------------TVSTTEAPPAPAVSNPQNEVDGSVA-PKECKQD-FMPHELCDKYYRCVHGKPTEFACRPGTVFHTQSNI 513
NvCht1    458 --------------------------------TQQTKPKPKPTVPPQPPTEIDESHNEIKCGEQD-FMAGKDCTSYYRCVYGKPLKFQCRDGLVYNPEKFI 525
DpCht2    468 -----------------EATSRPQSTSSEAPTRRTTKAPPAEGISPGSGGASNEIDYPKDCKGD-FMPHPDCTKYYRCDHGKPTEFSCQAGLVYHTVSHI 549
Clustal                                                              .          * *  .   * .**:* :  *  * *:  *::   *

RpCht6    548 CDWPQNVKREECKQ--- 561
PcCht1    587 CDWPENSRRPQCKNFKA 603
TcCht2    514 CDWPEHADREKCKKIEQ 530
TcCht5    514 CDWPEHADREKCKKIEQ 530
NvCht1    526 CDWPQNVDRPECQ---- 538
DpCht2    550 CNWPANADRDECRKE-- 564
Clustal        *:** :  * :*:
```

G

```
RpCht7       0 ---------------------------------------------------------------------------------------------------- 0
AmCht1       0 ---------------------------------------------------------------------------------------------------- 0
AdCht1       0 ---------------------------------------------------------------------------------------------------- 0
BiCht1       1 MEQITEPRVNLEGFRPLIIAMEIVGAILIILVAVWCGSYRGGFAWNSNPTLEFNWHPLLMVIGFVFLYANGMLIYRTQRNTRKRRLKLIHAGIMILIVAL 100
CbCht1       1 ---------------------------------------------------------------------------------------------------- 0
BtCht1       1 MEQITEPRVNLEGFRPLIVAMEIVGAILIILVAVWCGSYRGGFAWNSNPTLEFNWHPLLMVIGFVFLYANGMLIYRTQRNTRKRRLKLIHAGIMILIVAL 100
Clustal

RpCht7       1 ---------------------------------------------------------------------------------------------------- 0
AmCht1       1 -MVKQIPVPQAK----------YELLTDVRQS--------------------------------------------------------------RWKT-V 26
AdCht1       1 -MVNQIPVPQAK----------YELLTDVRQS--------------------------------------------------------------RWKTQI 27
BiCht1     101 VVISLVAVFDSHNLQLKPIPNMYSLHSWIGLTSVILFCCQWLAGFLSFLYPGLQLPLRASYMPIHVYFGIAGFVGVIASCLLGLNEKAFFALGPRWRTQV 200
CbCht1       1 -MVNQIPVPQAK----------YELLTDIRQP--------------------------------------------------------------RWRTQV 27
BtCht1     101 VVISLVAVFDSHNLQPKPIPNMYSLHSWVGLTSVILFCCQWLAGFLSFLYPGLQLPLRVSYMPIHVYFGIAGFVGVIASCLLGLNEKAFFALGPRWRTQV 200
Clustal

RpCht7       1 -------------------------------------------MANPFR-----------------------------------IVCYYVTPSNLTRSRD 22
AmCht1      27 LCLVLVSLILGILFVTRAWLGIIILRSSSTETFDTET-IDNAKVATWLRRARMYAESTKENQNANRNNSISSPQHFYTNSSGQIIVCYYTISSDLNTLWE 125
AdCht1      28 LCLVLVSLILGILFVIRAWLGIIILRSSRTETFNTET-TDNAKVATWLRRARMYAESTKENQNADRNNSISSPQHFYTNSSGQIIVCYYTISSDLNTLWE 126
BiCht1     201 LCLALVSLILGVLFVTRAWLGIVILRSPRAKTLDTETLTDNAKIATWLRRARMYAESTKENQNADRNNSISSPQHYNTNSSGQIIVCYYTISSDLNTFWE 300
CbCht1      28 VCLTLIFLIVGVLFITRAWFGILVLRAPKSTDRLAGERIEDAKIAAWLRRARMYAESTKENQNADRNTSVGASEQHYRNTTNQIVVCYYTIPGDLNTSWE 127
BtCht1     201 LCLALVSLILGVLFVTRAWLGIVILRSPGAKALDTETLTDDAKIATWLRRARMYAESTKENQNADRNNSISSPQHNNTNSSGQIIVCYYTISSDLNTFWE 300
Clustal                                                           :*  :*                                   :****. ..:*.   :

RpCht7      23 LPPNSVDPYLCTHIIIGFAIIQNATIQTRSPTDIQIYKTIVNLKNVNPQLKVLISVEDFSSDGE-FAKMVSSDELRTKFATNTLIFLNSTGFDGVDLDWE 121
AmCht1     126 LSPSNIDPNLCTHIIVGFAGVVNCSLNLGN--NSSIYKEVIGLKKFQPELRVMISVGGSNELHLGFSEMVKNHANRKRFIKSVLNVTRTFGFDGLDLDWE 223
AdCht1     127 LSPSNIDPNLCTHIIVGFAGVVNCSLNLGN--NSSIYKEVIALKKFQPELRVMISVGGNNELHLGFSEMVKNHANRKRFIKSVLNVTRTFGFDGLDIDWE 224
BiCht1     301 LSPSHIDPDLCTHIIVGFAGVVNCSLDLGS--NSSIYKEVIALKKLQPQLRVMISAGGSNELHLGFSEMVKNHANRKRFIRSVLNVTKTFGFDGLDLDWE 398
CbCht1     128 LSPSHIDPHICTHIIVGFASVVNSTLQVGN--NARLYEQVVALKNREPKLKVMISAGGINELHDGFPEMVKSHANRKKFIKSVLNVTKTFHIDGFDVDWE 225
BtCht1     301 LSPSHIDPDLCTHIIVGFAGVVNCSLDLGS--NSSIYEEVIALKKLQPQLRVMISAGGSNELHLGFSEMVKNHANRKRFIRSVLNVTKTFGFDGLDLDWE 398
Clustal        *.*. :** :*****:*** : *.:::  .  :  :*: :: **: :*:*:*:**. . ..    *.:**...  *.:*  ..* . .:  :**.*:***

RpCht7     122 FPSWPNADLIQVRNYTLLLKKFRHLINNSYMSSNKDFVLSVAVAAPELIMRQSYEIQQMSEQVEFVNLMSYDYHLYSQYLPLTGPNAPLYQRNAEEGYME 221
AmCht1     224 FPAWLGADEREKIRFIQLLEELR----KEFYRAKETLILSVAVAAPQAIVDQSYMVTEMAKYIDFVNLMSYDYHFYVWYYPITGLNAPLFSRAAESGYLS 319
AdCht1     225 FPAWLGADEREKIRFIQLLEELR----KEFYRAKETLILSVAVAAPQAIIDQSYMVIEMAKYIDFVNLMSYDYHFYVWYYPITGLNAPLFSRAAESGYLS 320
BiCht1     399 FPAWLGADEREKLHFVQLLEELR----REFYRATKTLILSVAVAAPQAIVDQSYMVAEMAKYVDFVNLMSYDYHFYVWYYPITGLNAPLFSHAAESGYLS 494
CbCht1     226 FPAWLGADDREKIHFVQLLQELR----KEFDRSGRKLLLTVAVAAPQAIIDQSYSVPEMAEHVDFINLMSYDYHFYVWYFPVTDLNAPLFPRATETGYLS 321
BtCht1     399 FPAWLGADEREKLHFVQLLEELR----REFYRATKTLILSVAVAAPQAIVDQSYMVAEMAKYVDFVNLMSYDYHFYVWYYPITGLNAPLFSHAAESGYLS 494
Clustal        **:* .**  :  .:  **:::*    ..: : .  ::*:******: *: *** : :*:: ::*:********:*  * *:*. ****: : :* **:.

RpCht7     222 TLNTNWSATHWVEWGMPWTKINVGIPTFGHSFRLINEDNNGWNAPASGIGKEGKGDGFVSYPEACKFIQNPSTTHVFDEEYEVPYAYNGKEWISYDSPVS 321
AmCht1     320 TLNVNFSVHYWLSKGMPGEKLIIGIPTYGHTYRLDNSLNHGLLAPANGFGKLGN-MGFVSYPTVCEFLRN-GAESVFEFESKVPYAYKDKEWISYDDITS 418
AdCht1     321 TLNVNFSVHYWLSKGMPGEKLIIGIPTYGHTYRLDNSLNHGLLAPANGFGKLGN-MGFVSYPTVCEFLRN-GAESVFEFESKVPYAYKDKEWISYDDVTS 419
BiCht1     495 TLNVNYSVHYWLSKGMPREKLIVGIPTYGHNYRLDNPQNHDLLAPANGFGELGK-MGFVSYPTVCEFLQN-GAKSVFKHESKVPYAYKDREWISYDDVTS 593
CbCht1     322 TLNVNFSAQYWLAKGMPREKIVVGVPTYGHSYTLDNPLNHNLQAPASGFGHLGT-MGFVSFPTVCQLLKS-GGASVFNKESRVPYAFKDKEWISYDNEES 420
BtCht1     495 TLNVNYSVHYWLSKGMPREKLIVGIPTYGHNYRLDNPQNHDLLAPANGFGELGK-MGFVSYPTVCEFLQN-GAKSVFEHESKVPYAYKDREWISYDDVKS 593
Clustal        ***.*:*. :*:  ***  *: :*:**:**.: * *  *:.  ***.*:*. *.  ****:* .*::::. .   **. * .****::.:******.  *

RpCht7     322 VELKARYVKNNKFGGAMIYSLNCDDFEGVCS--SIKFPLTRIVSNILMKD--- 369
AmCht1     419 IYYKAEWIRANNFGGAMILSLNVDDWNNTCK-FNESFPLTRTIMKILRYQEN- 469
AdCht1     420 ISYKAKWIRANNFGGAMILSLNVDDWNNTCK-FNESFPLTRTIMKILRYQEN- 470
BiCht1     594 VYYKAEWIRRNNFGGAMILSLNVDDWNNTCK-FNESFPLTRTVSKILRYQAD- 644
CbCht1     421 VYHKSTWIRANGFKGAMILSLNVDDWNGTCYGINETFPLTRAVSKIILQEADK 473
BtCht1     594 VCNKAEWIRANNFGGAMILSLNVDDWNNTCK-FNESFPLTRTVSKILRYQVD- 644
Clustal        :  *: ::: * * **** *** **::..*   . .***** : :*:  :
```

H

```
RpCht8       0 ---------------------------------------------------------------------------------------------------- 0
OnCht1       0 ---------------------------------------------------------------------------------------------------- 0
MbCht1       0 ---------------------------------------------------------------------------------------------------- 0
MrCht1       0 ---------------------------------------------------------------------------------------------------- 0
AmCht2       1 ------------------------------------------------------------------------------------------------MSQR 4
AmCht3       1 MRSHVVTLHIYRSYSINAKEVELLSRYTKRSARITVRVNIQSCTRGINGFSPETVSYRIIDVRSSYPHHFSSSSAGQGIKVSCEQVATNSPLLPFEKSQR 100
Clustal

RpCht8       1 ------MLLRLTACLLLCCVAY----LQAARVVCYWNGKSYWREGTAKVTTEEMKPGLSYCTHLLYGFTAIDDDDYHLEPVDKKLDLEKGKGQYRAVAEL 90
OnCht1       1 ------RLLAVVVSLCAIFATG----RAAGRVVCYWDGKSFWRDGAAKVTAEDIKPGLQYCSHLIYGYAGIHDDDYVVKSIDEDLVLDSGRGQYRAATNL 90
MbCht1       1 ----MNILIVLAGFLAIAGAVG----PSTSKVVCYYNSKSYVRESQARMLPLDLDPALSFCTHLVYGYAGVQPDTYKMVPLNENLDVDRSHANYRAITNF 92
MrCht1       1 ----MKVLLFAAIAVFCVQAVLSVDLHEHNKVVCYWNSTAFERQGPGKFQLDDVRPALSLCTHLIYGFAGIKSETYEVVPLNPNLDTGAGYSYYKLATQL 96
AmCht2       5 MVTKMKVLIFAAVAVFCVQSVFTVDTHEHNKVVCYWNTTAFERQGPGKFQLDDVQSALSLCTHLIYGFAGINAETFEVVPLKPSLDTGVGYSYYKLVTQL 104
AmCht3     101 MVTKMKVLIFAAVAVFCVQSVFTVDTHEHNKVVCYWNTTAFERQGPGKFQLDDVQSALSLCTHLIYGFAGINAETFEVVPLKPSLDTGVGYSYYKLVTQL 200
Clustal               *:  .  :     .         :****:: .:: *:. .:.   ::  ..*. *:**:**::.:. : : : .:. .*    . . *:  :::

RpCht8      91 KRAFPGLSVMLSVGGYEDNDD-PEKYFEVLEKAERRTKFINSVSTTLKQYGFDGIDLAWQFPPQHEKYETTSLSSIWHKIKKTFGA--GVDSKAAEHKDQ 187
OnCht1      91 KTFNSGLKVLLSIGGFGDTDD-LEKYYEVLENIERRTKFINSVHATLKQFNFDGVDLAWRFPEVKEKKDRNIFSSAWQGLKKAVGV--GVDEKWAEHRDQ 187
MbCht1      93 KTKYPGLKVLLSVGGDVDTED-ADKYNRLLESPQARTAFVNSGVLLAEQHGFDGIDLAWQFAKYKPKKIRSTWGSIWHGIKKTFGT-TPVDEKEAEHREG 190
MrCht1      97 KKSFPELKVYLSIGGNADPDDEVHKYLVLTETSEGRSKFINSVNRLFNDYDFDGIDLAWQFPPVKVKKERGTFGSIWHKVKKTFGYGKFKDDKEQEHRDG 196
AmCht2     105 KRTFPNLKIYLGIGGNADPDDETHKYLVLTETSQSRSKFINSVNRLLNDYDFDGIDLAWQFPPAKVKKERGTFGSIWHGIKKTFGYGKFKDDKEVEHRDG 204
AmCht3     201 KRTFPNLKIYLGIGGNADPDDETHKYLVLTETSQSRSKFINSVNRLLNDYDFDGIDLAWQFPPAKVKKERGTFGSIWHGIKKTFGYGKFKDDKEVEHRDG 300
Clustal        *   . *.: *.:**  * :*  .**  : *. : *: *:**     ::..***:****:*.  : *      .* *: :**:.*     *.*  **::

RpCht8     188 FVALVRELKAVLRAD-GKLLSIALLPHVNSTAYMDVRQLMPYVDMVNLWTTDYRTPERSPEKADFATPLYFEYPRLDFQNTDYTVRYWLEHGADGQKLNL 286
OnCht1     188 FVNLVRELKAALRTD-SKLLSLGVLPHINATAYYDVRNLAPQVDFVNLWTFDFRTPKRSKVQADYSAPLYYMYDRNPQQNADAHVTWWISNGCDPSKLNL 286
MbCht1     191 FTALVREMKQALNVKPNMQLAVTVLPNVNSTVYYDVPAIINLIDIVNIGAYDFETPERNPKEADYATPLYTPQNRNPLLNADAVVTYWLQGGAPAQKLIL 290
MrCht1     197 FTIMVRDLKAQLRPR-MKALTVTVLPHVNNSVYFDARLLAPNIDAVHLFTFDQKTPERNPQEADYPAPIYDSYGRVPQDNVDVPTRYWLENGTPGSKIVV 295
AmCht2     205 FTILVRDLKAQLRPR-LKDLTIGVLPHVNSTVYYDARLLAPNIDAVHLFTFDQKTPERNPREGDYPAPIYESYGRVPQDNVDSTARYWLEHGTPGSKIVV 303
AmCht3     301 FTILVRDLKAQLRPR-LKDLTIGVLPHVNSTVYYDARLLAPNIDAVHLFTFDQKTPERNPREGDYPAPIYESYGRVPQDNVDSTARYWLEHGTPGSKIVV 399
Clustal        *. :**::*  *.      *:: :**::* :.* *.  :   :* *:: : * .**:*.  :.*:.:*:*    *    *.*  . :*:. *   .*: :

RpCht8     287 GIATWGRSWKLTTDSGLSGVPPLVADGPGPAGPHTKTDGLLSYYETCVKLVSPTNPSAPAGMIRRMTDPSKRLGTYAFRLPDRRAGESEGLWVSFVEPET 386
OnCht1     287 GIPTFGRSWELTTDSPISGVPPLLADGAGDEGTYSKIKGTLAYYETCTRVVSPTNARAPSTLLRRVTDPTKRLGTYAYRLPQKDKDEEEGFWLSYEEPET 386
MbCht1     291 AIPTAARTWKLDADSEIAGVPPIHADGPGEAGPYTKTEGILSYPEVCAKLINPNHQKGMRPHLRKVTDPSKRFGTYAFRLPDEN--GDPGLWVSYEDPDT 388
MrCht1     296 GIPTYARTWKLTADSQISGVPPLVTDGPGAEGPHTNIPGLLSYPEVCARLTESAVGR-----LRRVGDPSKKYGSYAYQPYDEST-GADGIWVGYEDPDT 389
AmCht2     304 GIPTYARTWKLTSESQISGVPPIVTDGPGAEGPHTNTPGLLSYAEVCSRLTESAVGR-----LRRVGDPSKKYGSYAYQPYNENT-GADGIWVGYEDPDT 397
AmCht3     400 GIPTYARTWKLTSESQISGVPPIVTDGPGAEGPHTNTPGLLSYAEVCSRLTESAVGR-----LRRVGDPSKKYGSYAYQPYNENT-GADGIWVGYEDPDT 493
Clustal        .*.* .*:*:* ::* ::****: :**.*  *.:::  * *:* *.* :: ..         :*:: **:*: *:**::  :.      *:*:.: :*:*

RpCht8     387 AGYHAAYSKAKGLGGVALLDLALDDSRGICDGSKFPITRAAKLNL   431
OnCht1     387 AAYKAGYAKAKGLGGVVLVDLSLDDARGACDGTKFPILRSAKMNL   431
MbCht1     389 AGQRASYVKAKNLGGVAIIDLSMDDFRGLCTGDKYPILRAAKYRL   433
MrCht1     390 AGNKAAYVKAKGLGGVAIYDLSLDDFRGVCTGDKFPIVRGAKYKL   434
AmCht2     398 AGNKAAYAKAKGLGGVAIYDLSLDDFRGVCTGDKYPIIRAAKYKL   442
AmCht3     494 AGNKAAYAKAKGLGGVAIYDLSLDDFRGVCTGDKYPIIRAAKYKL   538
Clustal        *. :*.* ***.****.: **::** ** * * *:** *.** .*
```

I

```
RpCht9         1 MPPSISGDELLDVSYAMANKGLPSMALAANPDSLYKILFQMGRISEQLVHVRENFTRCNTSSCPETKQFMLIMERNIRRLFHFFLLCTLIAVVLTDSSSS 100
PxCht1         1 ----------------------------MWPPSLLRWSAAILLVLAVLTPISDSSG---------------AVRRRLRKP----------TNKVSTSVTS 47
AcypiCht2      1 ---------------------------MTNRKYKLCFHFKLLLIVTIVATLCSYTNSE-------------TIRRRLRRPTGNKLQQ--ETTAVSDSSAA 58
OfCht1         1 ----------------------------MWPPRLLSCIFTIILIVAVIAPTSDST----------------TVRRRLRK-----------PSK-SVSVTS 44
BmCht1         1 MVPTGDSHYHALLSRDFPEITRPAGTQRTRPPILLRWMLTVVLVIAVIAPTSDST----------------TVRRRLRK-----------PSKVSTSVSS 73
BmCht2         1 ----------------------------MRPPILLRWMLTVVLVIAVIAPTSDST----------------TVRRRLRK-----------PSKVSTSVSS 45
Clustal                                                  :  :   :.   .                   :.*.:*:           .   : * ::

RpCht9       101 TRRRLRRPSGASRVRDQETSASVNRFKVRNRVSQNSTPIISRKSSNEGSS--SSSNDGYKVVCYYTNWSQYRQKIGKFLPEDIDPDLCTHIIFAFGWLKK 198
PxCht1        48 S---------VSRSSDQIISASVNRPKIRGRPSVASRKSSAAIDNSVNTE-DHKDKDGYKVVCYYTNWSQYRTKIGKFVPEDISPDLCTHMIFAFGWLKK 137
AcypiCht2     59 A---------TSTVVDMETSASVNKFKIRNRTATRKSGTDASTEKHVTTAKDNQDEGGYKIVCYYTNWSQYRPKTGKYLPEDIDPELCTHVIFAFGWLKK 149
OfCht1        45 S---------VSRSTDQVISASVNRPKIRGRPSVASRKSSAALDNSVGTD-DHKDKDGYKIVCYYTNWSQYRTKIGKFMPEDIQPELCTHIIFAFGWLKK 134
BmCht1        74 S---------VSRSTDQVLSASVNRPKIRGRPSIASRKSSAALDNSVTTE-HHKDKDGYKIVCYYTNWSQYRTKVGKFVPEDIQPDLCTHIIFAFGWLKK 163
BmCht2        46 S---------VSRSTDQVLSASVNRPKIRGRPSIASRKSSAALDNSVTTE-HHKDKDGYKIVCYYTNWSQYRTKVGKFVPEDIQPDLCTHIIFAFGWLKK 135
Clustal          :         .*   *   *****: *:*.* :  .    :  ..   :    ..:.***:*********** * **::****.*:****:*********

RpCht9       199 GKLSSFESNDETKDGKIGLYERIRNLKKANPKLKTLLAIG------------------------YAIPFLRQRHFDGLDVDWEYPKGGDDKKNFVLLLKE 274
PxCht1       138 GKLSSFESNDETKDGKTGLYDRINALKKGNPKLKTLLAIGGWSFGTQKFKEMSATRYARQTFIYSSIPYLRDRNFDGLDIDWEYPKGGDDKKNYVLLLKE 237
AcypiCht2    150 GRLSSFESNDESKDGKVGLYDRIKNLKKANPKLKTLLALGGWSFGTQKFKDMSATRYARQTFIYSAIPFLRARSFDGLDLDWEYPKGQDDKKNFVHLLKE 249
OfCht1       135 GKLSSFESNDETKDGKTGLYDRINALKKANPKLKTLLAIGGWSFGTQKFKEMSATRYARQTFIYSAIPYLRDRNFDGLDIDWEYPKGGDDKKNFVLLLKE 234
BmCht1       164 GKLSSFESNDETKDGKTGLYDKINGLKKGNPKLKTLLAIGGWSFGTQKFKDMVATRYARQTFIYSAIPYLRDRNFDGLDIDWEYPKGGDDKKNYVLLLKE 263
BmCht2       136 GKLSSFESNDETKDGKTGLYDKINGLKKGNPKLKTLLAIGGWSFGTQKFKDMVATRYARQTFIYSAIPYLRDRNFDGLDIDWEYPKGGDDKKNYVLLLKE 235
Clustal          *:*********:**** ***::*. ***.*********:*                         :**:** * *****:******* *****:* ****

RpCht9       275 LREAFEAEAQEVKKPRLLLSAAVPVGPDNVRGGYDVPAVAGYLDFINLMAYDFHGKWERETGHNAPLYAPSTDSEWRKQLSVSAAAEMWTRLGAPKEKLV 374
PxCht1       238 LREAFEAEAQEVKKPRLLLTAAVPVGPDNIKSGYDVPAVASYLDFINLMAYDFHGKWERETGHNAPLYAPSTDSEWRKQLSVDHAAHLWVKLGAPKEKLI 337
AcypiCht2    250 LREAFEAEAQEVKKPRLLLTAAVPVGPDNIKGGYDVPAVSSYLDFINVMAYDFHGKWERETGHNAPLYSPSSDSEWRKQLSVDNAASIWTKMGAPKEKLI 349
OfCht1       235 LREAFEAEAQEVKKPRLLLTAAVPVGPDNIKSGYDVPAVASYLDFINLMAYDFHGKWERETGHNAPLYAPSSDSEWRKQLSVDHAAHLWVKLGAPKEKLI 334
BmCht1       264 LREAFEAEAQEVKKPRLLLTAAVPVGPDNIKSGYDVPAVASYLDFINLMAYDFHGKWERETGHNAPLYAPSTDSEWRKQLSVDHAAHLWVKLGAPKDKLI 363
BmCht2       236 LREAFEAEAQEVKKPRLLLTAAVPVGPDNIKSGYDVPAVASYLDFINLMAYDFHGKWERETGHNAPLYAPSTDSEWRKQLSVDHAAHLWVKLGAPKDKLI 335
Clustal          *******************:*********::.*******:.******:********************:**:**********. ** :*.::****:**:

RpCht9       375 IGMPTYGRTFTLTSTQRIGVNSPASGGGKAGEYTKEGGFLAYYEICEMLRNGASYVWDDEMKVPYAIQGDQWVGFDDEKSIRYKMKWLKENGYAGAMVWT 474
PxCht1       338 IGMPTYGRTFTLSNQNNFKVNSPASGGGKAGEYTKESGFLAYYEVCEMLRNGATYVWDDEMKVPYAISGDQWVGFDDEKSIRHKMRWIKDNGFGGAMVWT 437
AcypiCht2    350 IGMPTYGRTFTLTNTANYKVNSPASGGGKAGEYTKEAGFLAYYEICEILKNGATYVWDDEMKVPYAISGDQWIGFDDERSIRNKMKWIKDNGYGGAMVWT 449
OfCht1       335 IGMPTYGRTFTLSNPNNFKVNSPASGGGKAGEYTKESGFLAYYEVCEILRNGGAYVWDDEMKVPYAIHGDQWVGFDDEKSIRNKMRWIKDNSFGGAMVWT 434
BmCht1       364 IGMPTYGRTFTLSNPNNFKVNAPASGGGKAGEYTKESGFLAYYEVCEMLRNGGAYVWDDEMKVPYCVHGDQWVGFDDEKSIRNKMRWIKDNGFGGAMVWT 463
BmCht2       336 IGMPTYGRTFTLSNPNNFKVNAPASGGGKAGEYTKESGFLAYYEVCEMLRNGGAYVWDDEMKVPYCVHGDQWVGFDDEKSIRNKMRWIKDNGFGGAMVWT 435
Clustal          ************:.  .  **:**************.*******:**:*:**.:***********.: ****:*****:*** **:*:*:*.:.******

RpCht9       475 IDMDDFNGTVCGNGVKYPLIGAIREELRGIKRGPNAQDVDWSKVAGSVSTTQLAKPVAIKIPVSDVLTRLN---KVKPTASNAIIPIQDLNSREAQVFCY 571
PxCht1       438 VDMDDFSGTVCGGDVKYPLIGAMREELRGISRGKDAQDVDWASVANSVIVEVTEKPEPIKISLSDVISRAKKPTKTLVIKNNN--AALDKNKREPQVLCY 535
AcypiCht2    450 VDMDDFTGTFCS-DVKYPLIGAMREELRGIKRGK--EDIDWSKVANTIIAETITKPSPIKISVSEVLSKTK-PSK-IQASTAIQTDVVDMNVRAPQVMCY 544
OfCht1       435 VDMDDFSGGVCGGNVKYPLIGAMREELRGISRGKDAKDVDWASVAASVVIEVTEKPQPIKISLSDVLSRVKKPTKKLVITNNNNAAILDKNKREPQVLCY 534
BmCht1       464 VDMDDFSGNVCGGDVKYPLIGAMREELRGISRGKDAKDVDWATVASSIVIEETEKPEPIQVPLSEVLKRIKKPGKNVIIKTNG-AALSDKNKREPQVLCY 562
BmCht2       436 VDMDDFSGNVCGGDVKYPLIGAMREELRGISRGKDAKDVDWATVASSIVIEETEKPEPIQVPLSEVLKRIKKPGKNVIIKTNG-AALSDKNKREPQVLCY 534
Clustal          :*****.* .*. .********:*******.**   :*:**:.** ::      ** .*::.:*:*:.: :   *     .       * * * .**:**
```

```
RpCht9     572 LTSWSSKRPGAGRFSPSDLQPTLCTHIIYAFATLTDHKLASASG-TEDQYHKIVSLREKNPNLKILLAIGGWAFGSTPFKELTSNVFRMNQFVYEAIEFL 670
PxCht1     536 LTSWSSKRPSSGRFTPENVDPMLCTHLIYAFATLKDHKLTEGDEKDADMYDKVVALREKNPNLKILLAIGGWAFGSTPFKELTSNVFRMNQFVYEAIEFL 635
AcypiCht2  545 MTSWSIKRPGAGKFTPDNIDPSLCTHVIYAFGSLKDFKLTFVDEKDTEQYKEMMALREKNANLKVLLAIGGWAFGSTPFKELTGNVFRMNQFVYEAIEFL 644
OfCht1     535 LTSWSSKRPSAGRFMPENVDPTLCTHVIYAFATLKDHKLTEADEKDADMYDKVVALREKNPNLKILLAIGGWAFGSTPFKELTSNVFRMNQFVYEAIEFL 634
BmCht1     563 LTSWSAKRPSAGRFTPENVDPKLCTHIIYAFATLKDHKLAEADDKDADMYDKVVALREKNPNLKILLAIGGWAFGSTPFKELTSNVFRMNQFVYEAIEFL 662
BmCht2     535 LTSWSAKRPSAGRFTPENVDPKLCTHIIYAFATLKDHKLAEADDKDADMYDKVVALREKNPNLKILLAIGGWAFGSTPFKELTSNVFRMNQFVYEAIEFL 634
Clustal        :**** ***.:*:* *.:::* ****:****.:*.*.**:  .     : *.::::*****.***:*****************.***************

RpCht9     671 REYQFNGLDVDWEYPRGIDDRASYVNLLRELRTAFEGEAKSSGQPRLMLTAAVPASFEAIAAGYDVPEIAKYLDFINVMTYDFHGQWERQVGHNSPLYPL 770
PxCht1     636 RDYQFNGLDVDWEYPRGADDRAAFVNLLKELRLAFEGEAKSSGQPRLLLTAAVPASFEAIAAGYDVPEISKYLDFINVMTYDFHGQWERQVGHNSPLFPL 735
AcypiCht2  645 REHKFNGLDVDWEYPRGLDDKAAYVNLLKELRLAFEGESKSSGLPRLLLTAAVPASFEAIAAGYDVVEISKYLDFINVMTYDFHGQWERQVGHNSPLYPL 744
OfCht1     635 RDYQFNGLDVDWEYPRGADDRAAFVSLLKELRLAFEGEAKTSGQPRLLLTAAVPASFEAIAAGYDVPEISKYLDFINVMTYDFHGQWERQVGHNSPLFPL 734
BmCht1     662 RDYQFNGLDVDWEYPRGADDRAAFVSLLKELRLAFEGEAKTSGQPRLLLTAAVPASFEAIAAGYDVPEISKYLDFINVMTYDFHGQWERQVGHNSPLFPL 762
BmCht2     635 RDYQFNGLDVDWEYPRGADDRAAFVSLLKELRLAFEGEAKTSGQPRLLLTAAVPASFEAIAAGYDVPEISKYLDFINVMTYDFHGQWERQVGHNSPLFPL 734
Clustal        *:::************ **:*::*.**:***  *****:*:** ***:***************** **:*************************:**

RpCht9     771 ESATSYQKKLTVDFSAREWVKQGAPKEKLMIGMPTYGRSFTLVDEAKFDIGAPASGGGKPGNYTAESGFMAYYEICDFLHEDNTTLVWDNEQQVPFAYRG 870
PxCht1     736 ESATSYQKKLTVDYSAREWVRQGAPKEKLMIGMPTYGRSFTLINETQFDIGAPASGGGQAGRYTNEAGFMSYYEICEFLREDNTTLVWDNEQMVPFAYRD 835
AcypiCht2  745 ESATSYQKKLTVDYSAREWVKQGAPKEKLMIGMPTYGRSFTLVDPAKFDIGAPASGGGTAGKYTSEAGFMAYYEVCDFLHVENTTLVWDNEQQVPFAYRK 844
OfCht1     735 ESATSYQKKLTVDYSAREWVRQGAPKEKLMIGMPTYGRSFTLINDTQFDIGAPASGGGQAGRFTNEAGFMSYYEICEFLREDNTTLVWDNEQMVPFAYRE 834
BmCht1     763 ESATSYQKKLTVDYSAREWVRQGAPKEKLMIGMPTYGRSFTLINETQYDIGAPASEGGEAGRFTNEAGFMSYYEICEFLREDNTTLVWDNEQMVPFAYRG 862
BmCht2     735 ESATSYQKKLTVDYSAREWVRQGAPKEKLMIGMPTYGRSFTLINETQYDIGAPASEGGEAGRFTNEAGFMSYYEICEFLREDNTTLVWDNEQMVPFAYRG 834
Clustal        *************:******:******************:: :::******* ** .*.:* *:***:***:*:**: :********** ******

RpCht9     871 NQWVGFDDERSLKTKMGWLKEEGFGGIMIWSIDMDDFRGVCGNGKFPLVNAMKQELEDYTIKLEYQGPYEGTGGGGAYTTKDPNAVTCEEEDGHISYHPD 970
PxCht1     836 DQWVGFDDERSLKTKMAWLKEEGFGGIMVWSIDMDDFRGSCGTGKYPLITAMKQELSGYKVKLEFDGPYESSNPNGQYTTKDPNEVTCEEEDGHISYHPD 935
AcypiCht2  845 DQWIGFDDERSLKTKMSWLKEEGFGGVMIWSVDMDDYRGVCGTGKYPLINAMKQELAGYTVKLEYDGPYEATGRNTVYTTKDPTSVTCEEEDGHISYHPD 944
OfCht1     835 DQWVGFDDERSLKTKMAWLKEEGFGGIMVWSVDMDDFRGSCGTGKYPLITAMKQELSGYKVKLEYDGPYESSSPTGQYTTKDPHEVTCEEEDGHISYHKD 934
BmCht1     863 DQWVGFDDERSLKTKMAWLKEEGFGGIMVWSVDMDDFRGSCGTGKFPLITTMKQELSDYKVKLEYDGPYETVLTSGQYTTKDPTEVTCEEEDGHISYHKD 962
BmCht2     835 DQWVGFDDERSLKTKMAWLKEEGFGGIMVWSVDMDDFRGSCGTGKFPLITTMKQELSDYKVKLEYDGPYETVLTSGQYTTKDPTEVTCEEEDGHISYHKD 934
Clustal        :**:************.*********:*:**:****:** **.**:**:.:***** .*.:***::****       ******  ************ *

RpCht9     971 KADCTMYYMCEGERKHHMPCPSNLVFNPNENVCDWPENVEGCSQHTQAPPS--- 1021
PxCht1     936 KADCTMYYMCEGERKHHMPCPSNLVFNPNENVCDWPENVEGCQHHTQAPAAKR- 988
AcypiCht2  945 KADCTMYYMCEGERKHHMPCPSNLVFNPKENVCDWPENVEGCMQHTPAPPTSRR 998
OfCht1     935 HADCTMYYMCEGERKHHMPCPSNLVFNPNENVCDWPENVEGCQQHTQAPAAKR- 987
BmCht1     963 QADCTMYYMCEGERKHHMPCPSNLVFNPNENVCDWPENVEGCAHHTQAPPARR- 1015
BmCht2     935 QADCTMYYMCEGERKHHMPCPSNLVFNPNENVCDWPENVEGCAHHTQAPPARR- 987
Clustal        :**************************:************* :** **.:
```
