# Supplementary material for: Structure and expression of Rhodnius prolixus GH18 chitinases and chitinase-like proteins: Characterization of the physiological role of RpCht7, a gene from subgroup VIII, in vector fitness and reproduction
Source: Front Physiol. 2022 Oct 3;13:861620. doi: 10.3389/fphys.2022.861620 (PMC9574080; doi:10.3389/fphys.2022.861620)
Supplement: Supplementary file 4 [file Table3.DOCX]

| Number of exons from chitinase sequences | | | |
| --- | --- | --- | --- |
| RpCht1 - 46 | PhCht4 - 34 | AgCht10 - 9 | AaCht10 - 9 |
| RpCht2 - 18 | PhCht5 - 18 |  | AaCht6 - 9 |
| RpCht4 - 7 |  | AgCht12 - 5 | AaCht12 - 5 |
| RpCht5 - 7 | PhCht3 - 7 | AgCht2 - 4 | AaCht2 - 6 |
| RpCht6 - 9 | PhCht7 - 9 | AgCht5-5 | AaCht5-1 -6 |
| RpCht7 - 7 | PhCht6 - 6 | Ag Cht11 - 5 | AaCht11 – 4 (range 4-7) |
| RpCht8 - 7 |  | AgIDGF - 3 | AaIDGF - 3 |
| RpCht9 - 17 | PhCht2 - 16 | AgCht7 - 8 | AaCht7 - 8 |
