# Supplementary material for: Structure and expression of Rhodnius prolixus GH18 chitinases and chitinase-like proteins: Characterization of the physiological role of RpCht7, a gene from subgroup VIII, in vector fitness and reproduction
Source: Front Physiol. 2022 Oct 3;13:861620. doi: 10.3389/fphys.2022.861620 (PMC9574080; doi:10.3389/fphys.2022.861620)
Supplement: Supplementary file 5 [file Table4.DOCX]

| RpCht | MW(KDa) RpCht | Group | MW(KDa) Group |
| --- | --- | --- | --- |
| 6 | 64 | I | 60 - 70 |
| 1 | 297 | II | 300 |
| 9 | 118 | III | 120 |
| 4 | 49 | IV | 50 |
| 8 | 48 | V | 50 |
| 2 | 138 | VI | 200-500 |
| 5 | 38 | VII | 40-50 |
| 7 | 50 | VIII | 30-60 |
